# Metabolism-driven in vitro/in vivo disconnect of an oral ERɑ VHL-PROTAC

**DOI:** 10.1038/s42003-024-06238-x

**Published:** 2024-05-13

**Authors:** Thomas G. Hayhow, Beth Williamson, Mandy Lawson, Natalie Cureton, Erin L. Braybrooke, Andrew Campbell, Rodrigo J. Carbajo, Azadeh Cheraghchi-Bashi, Elisabetta Chiarparin, Coura R. Diène, Charlene Fallan, David I. Fisher, Frederick W. Goldberg, Lorna Hopcroft, Philip Hopcroft, Anne Jackson, Jason G. Kettle, Teresa Klinowska, Ulrike Künzel, Gillian Lamont, Hilary J. Lewis, Gareth Maglennon, Scott Martin, Pablo Morentin Gutierrez, Christopher J. Morrow, Myria Nikolaou, J. Willem M. Nissink, Patrick O’Shea, Radoslaw Polanski, Markus Schade, James S. Scott, Aaron Smith, Judith Weber, Joanne Wilson, Bin Yang, Claire Crafter

**Affiliations:** 1 Oncology R&D, AstraZeneca, Cambridge, UK; 2 Pharmaceutical Sciences, AstraZeneca, Cambridge, UK; 3 Discovery Sciences, R&D, AstraZeneca, Cambridge, UK; 4 Oncology R&D, AstraZeneca, Waltham, MA USA

**Keywords:** Lead optimization, Small molecules

## Abstract

Targeting the estrogen receptor alpha (ERα) pathway is validated in the clinic as an effective means to treat ER+ breast cancers. Here we present the development of a VHL-targeting and orally bioavailable proteolysis-targeting chimera (PROTAC) degrader of ERα. In vitro studies with this PROTAC demonstrate excellent ERα degradation and ER antagonism in ER+ breast cancer cell lines. However, upon dosing the compound in vivo we observe an in vitro*-*in vivo disconnect. ERα degradation is lower in vivo than expected based on the in vitro data. Investigation into potential causes for the reduced maximal degradation reveals that metabolic instability of the PROTAC linker generates metabolites that compete for binding to ERα with the full PROTAC, limiting degradation. This observation highlights the requirement for metabolically stable PROTACs to ensure maximal efficacy and thus optimisation of the linker should be a key consideration when designing PROTACs.

## Abstract (web summary)

An orally available VHL-ERα PROTAC was developed that showed excellent degradation in vitro. When dosing in vivo, the degradation of ERα was lower than expected, due to competitive binding at the ERα binding site between the PROTAC and a linker metabolite.

## Introduction

Female breast cancer is now the most commonly diagnosed cancer and accounts for 1 in 4 cases in women. In 2020, there were over 2.2 million new cases of breast cancer diagnosed and 685,000 deaths worldwide^1^. Approximately 70% of breast cancer patients are estrogen receptor positive (ER+) and their tumours depend on activation of the receptor for growth and survival^2–5^. As such, multiple modulators of the ER pathway have been developed. These include aromatase inhibitors, e.g. anastrozole, that act indirectly to reduce the production of estrogen, or selective estrogen receptor modulators, e.g. tamoxifen, and selective ER degraders (SERDs), e.g. fulvestrant, that bind and antagonise ERα and in the latter case lead to its degradation through the 26S proteasome^6,7^. Fulvestrant, first approved in 2002, has shown superior efficacy to anastrozole in clinical studies and is now a common treatment option for patients with ER+ locally advanced or metastatic disease in the first-line setting^8^. However, the physicochemical properties of fulvestrant and administration via monthly intramuscular injections limit its exposure in the clinic. This has fuelled interest in the development of orally bioavailable SERDs with the belief that these could achieve improved exposure and therefore greater clinical response^9^.

An alternative new and exciting approach to achieve protein degradation is through the use of proteolysis targeting chimeras (PROTACs)^10,11^. These heterobifunctional small molecules consist of two ligands, one which binds to the protein of interest (POI) and one which binds to an E3 ubiquitin ligase (E3 ligase), connected by a linker. This allows the POI to be brought in close proximity to the E3 ligase, leading to polyubiquitination and subsequent proteasome-mediated degradation^12^.

Degrading ERα is a clinically validated approach but there is still a need to develop agents with improved exposure that deliver improved efficacy. PROTACs, with their unique mechanism of action, may provide an opportunity to achieve this. Several groups have reported ERα-targeting PROTACs using a range of E3 ligases including Skp1-Cullin-F-box, VHL, CRBN and cIAP1. These have employed a range of ERα-binders and demonstrated the effective degradation of ERα in vitro^13,14^. However, the majority of these ERα PROTACs represent in vitro tools rather than compounds for future clinical development. The exception is ARV-471, which uses an IMiD binder to recruit cereblon E3 ligase and has shown oral bioavailability both preclinically and more recently in clinical trials^15^.

Our group had access to a range of novel ERα binding motifs to use as POI ligand start points through prior discovery of first-generation SERDs, such as AZD9496 and next-generation SERD camizestrant (AZD9833)^16,17^. Here we describe the discovery of AZ’6421 (**5**), a potent and orally bioavailable ERα PROTAC that degrades and antagonises ERα, and uncover an unexpected disconnect between the in vitro and in vivo degradation efficacy. We highlight the importance of understanding metabolic liabilities and the impact that metabolites can have on PROTAC activity in vivo.

## Results

### AZ’6421, an ERα-targeting bifunctional degrader that binds to VHL

**Fig. 1:**
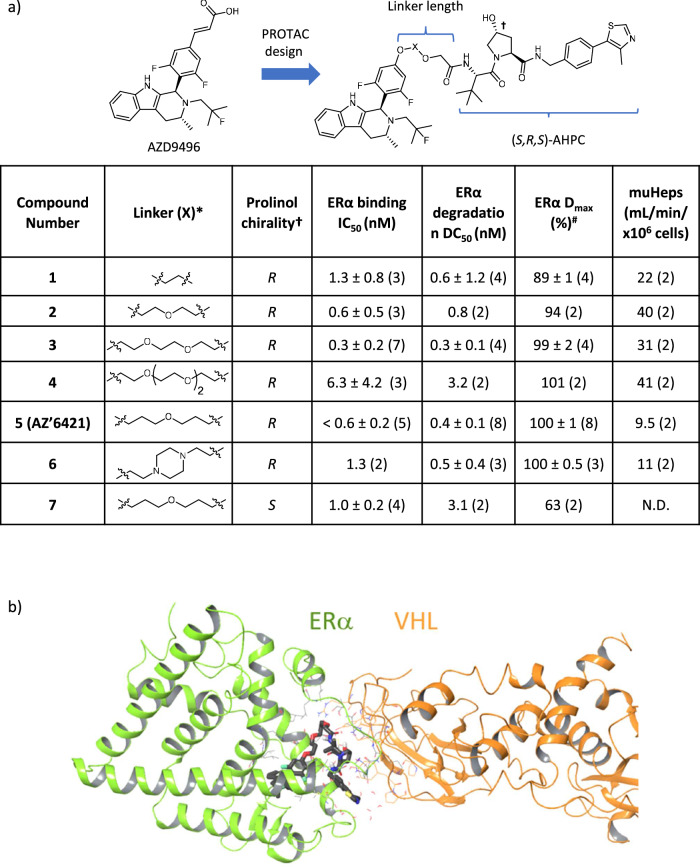
Data for compounds discussed in the manuscript. **a** Redesign of AZD9496 to VHL-PROTACs. Linker length is measured from the oxygen of the phenyl ether to the carbonyl of the amide. # Maximum level of degradation observed for the compound where 0% is vehicle control and 100% is maximal concentration observed for fulvestrant. Data presented is the mean across replicates with standard deviation noted as the ± value. Numerical values in brackets are the number of replicates. ND not determined. muHeps mouse hepatocytes. **b** Approximate ternary complex of estrogen receptor, PROTAC, and VHL; snapshot from molecular dynamics (see Supplementary Note for details).

We based our ERα PROTACs on the AZD9496 core, removing the acrylic acid and replacing it with ether-linked PEG chains to the VHL binding group (*S*,*R*,*S*)-AHPC (Fig. 1a). By changing the number of PEG units in the linker a range of linker lengths were assessed with compounds **1**-**4**. It was established that compound **3** with a linker consisting of 3 PEG monomers offered the highest degradation potency (DC_50_) of 0.3 nM and degradation efficiency (D_max_) of 99% compared to fulvestrant in our imaging degradation assay (Fig. 1a). Hepatic clearance of all PEG-containing compounds in mouse hepatocytes proved to be too high to enable progression into mouse pharmacokinetic (PK) studies. We therefore modified the linker, adjusting the composition while keeping approximately the same length between the VHL and ERα binding moieties. The optimisation found linear linkers had improved potency and degradation compared to compounds with conformational constraint in the linker (selected examples in Table S4). From this investigation compounds that displayed ≥100% degradation in the assay, equalling fulvestrant, and a sub-nanomolar DC_50_ were profiled in mouse hepatocytes. Compound **5**, which contained 2 propyl ethers, and piperazine containing **6**, a constrained linker that unusually displayed full degradation of ERα, demonstrated improved clearance, compared to **3** in mouse hepatocytes of 9 and 11 mL/min/10^6^ cells respectively (Fig. 1a). To further differentiate the compounds we assayed them in the nanoluciferase degradation assay, which showed more dynamic range than the imaging assay. Of the two compounds, **5** showed greater potency in the nanoluciferase assay and lower mouse hepatic clearance than **6** (Table 1 and Fig. 1a). Therefore, we identified **5**, referred to as AZ’6421 henceforth, for further study as it had excellent potency, degradation and hepatic clearance suitable for PK and pharmacodynamic (PD) studies in mouse.

**Table. 1:** Comparison of imaging and nano-luciferase degradation assays

Compound Number	Linker (X)	ERα imaging degradation DC_50_ (nM)	ERα imaging D_max_ (%)^#^	ERα nano-luciferase degradation DC_50_ (nM)	ERα nano-luciferase D_max_ (%)^#^
**1**		0.6 ± 1.2 (4)	89 ± 1 (4)	ND	ND
**2**		0.8 (2)	94 (2)	ND	ND
**3**		0.3 ± 0.1 (4)	99 ± 2 (4)	3.1 (2)	124 (2)
**4**		3.2 (2)	101 (2)	ND	ND
**5 (AZ’6421)**		0.4 ± 0.1 (8)	100 ± 1 (8)	15 ± 9 (8)	121 ± 15 (8)
**6**		0.5 ± 0.4 (3)	100 ± 0.5 (3)	46 ± 27 (3)	88 ± 1 (3)
7*		3.1 (2)	63 (2)	>3000 (1)	NV (1)

^#^Maximum level of degradation observed for the compound where 0% is vehicle control and 100% is maximal concentration observed for fulvestrant. * (*S*)-chirality at prolinol (**†**) Data presented is the mean across replicates with standard deviation noted as the ± value. Numerical values in brackets are the number of replicates. *NV* no value. *ND* not determined.

The lipophilicity of AZ’6421 was determined to be 6.6 by a chromatographic LogD (chromLogD) assay^18^, which lies well beyond the LogD range of 1-3 that is more typically observed for oral drug-like small molecules^19^. An experimental PSA (ePSA) value of 98 Å^2^ was determined using a chromatographic method^20^, reflecting a lower level of polarity than suggested by its calculated topological PSA (TPSA) of 158 Å^2^ ^21^. A folded conformation has been observed in the crystal structures of ternary complexes with VHL-targeting PROTAC molecules, which may result in similar effects^22–24^. We were interested in determining whether a collapsed conformation could form in solution. Solution NMR spectroscopy was used to study the 3D conformational behaviour and intramolecular H-bond (IMHB) properties of AZ’6421 (Supplementary Note 1, Figs S1–S4, Tables S1-S3).

We identified the linker ether oxygen proximal to the VHL warhead as a hydrogen-bond acceptor (HBA) for the shielded hydrogen-bond donor (HBD) of AZ’6421 (Figs 1b and S1). The IMHB between the amide joining the linker to the (*S,R,S*)-AHPC and the adjacent linker oxygen was previously observed in the crystal structure of the ternary complex between PROTAC MZ1 and cognate proteins Brd4 and VHL^25^. Long-range NOEs between the *tert*-butyl group and the benzyl-amide in (*S,R,S*)-AHPC, specific intramolecular hydrogen-bonds and ^1^H-^13^C long-range correlations were observed indicating that AZ’6421 adopts a conformation that is partially collapsed around the *tert*-butyl, which is facilitated by hydrophobic contacts (Figs S1 and S2). Taken together, the conformations seen in the solution are not incompatible with the folded conformations of VHL PROTACs that have been observed in ternary-complex crystal structures of SMARCA and Brd4 proteins^25,26^.

Next, we constructed a ternary-complex model to establish whether the ensemble of conformers observed in the solution would be compatible with the ligand conformations needed to form a ternary complex (Supplementary Note 2). No ternary structures have been reported for ERα with VHL, so we built initial models using known ternary complex structures of Brd4 with VHL^25^. Only one resulting model of ER and VHL tethered by AZ’6421 was deemed suitable and was used to run a 200 ns molecular dynamics (MD) simulation (Figs S5-S8). The results suggested an initial rearrangement of the position of ERα relative to VHL and subsequent further relaxation of the ternary complex over the course of the simulation. An example ternary structure snapshot shows the complex (Fig. 1b). Inspection of conformations of the PROTAC bound in its ternary complex suggested that NOE-derived distances seen for the folded PROTAC in solution are also present in the ternary-bound, folded conformations of the PROTAC.

**Fig. 2:**
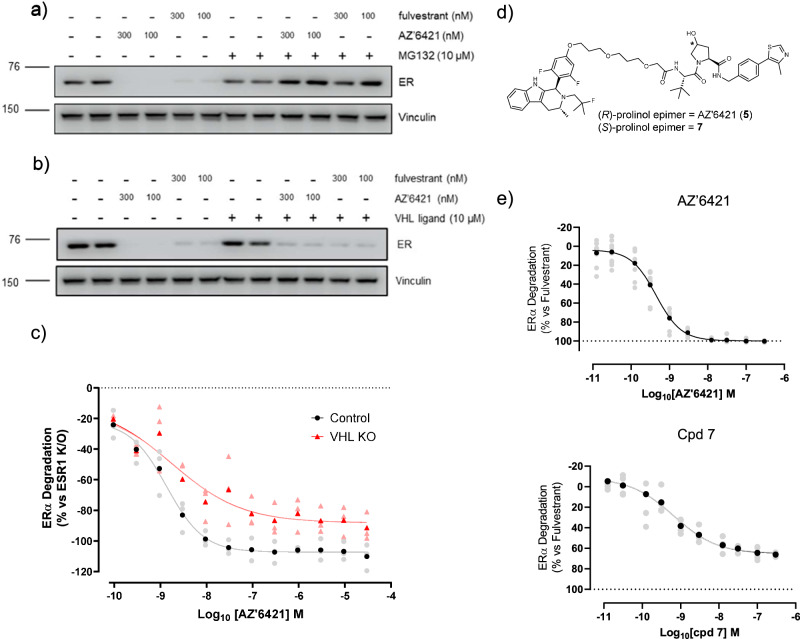
AZ’6421 degrades ERα through a PROTAC mechanism of action. **a** Immunoblots for ERα and vinculin loading control. MCF7 cells were pre-treated with 10 µM MG132 or DMSO control for 1 h before being exposed to DMSO, AZ’6421, or fulvestrant, at the concentrations indicated, for a further 7 h. A representative immunoblot from two biological replicates is shown. **b** As in **a** but cells were pre-treated with 10 µM VHL ligand (Ac-(*S,R,S*)-AHPC) prior to addition of DMSO, AZ’6421 or fulvestrant. **c** MCF7 cells expressing BFP-Cas9 were reverse transfected with the crRNA:tracrRNA pool targeting VHL for 72 h prior to treatment with increasing concentrations of AZ’6421 for a further 24 h. Cells were then fixed, stained, and imaged to assess ERα and Cas9 expression. ERα levels in Cas9 positive cells were normalised to unedited cells (0) and ESR1 KO cells (-100). Data points are from three independent experiments, points in bold represent the mean. **d** Structure of AZ’6421 and the (*S*)-prolinol epimer, **7**. **e** Concentration-response curves for AZ’6421 and 7. ERα levels in MCF7 cells were assessed using an immunofluorescence endpoint after 24 h exposure to compound. Individual data points were normalised to vehicle control (0% inhibition) and 100 nM fulvestrant (100% inhibition). Data points are from 5 (AZ'6421) or 2 (Cpd **7**) independent experiments with technical duplicates performed for each experiment, bold points represent the mean. All concentration-response curves were plotted using nonlinear regression analysis in GraphPad Prism.

The model indicated that AZ’6421 could bind to both ERα and VHL with relatively limited rearrangement of its ensemble in solution. Together with the NMR conformation, this suggested that AZ’6421 had the potential to form a ternary complex with ERα and VHL and could therefore degrade ERα through a PROTAC-induced mechanism. However, since the ERα binding portion was based on a moiety with known SERD activity we wanted to confirm that the molecule was indeed behaving as a PROTAC. Pre-treatment with MG132, a proteasome inhibitor, attenuated ERα degradation in response to AZ’6421 and fulvestrant, confirming that both agents degrade ERα via the proteasome (Fig. 2a). In contrast, pre-treatment with excess acetylated-(*S,R,S*)-AHPC, which competes with the PROTAC for binding to VHL, partially reduced ERα degradation in response to AZ’6421 but had no effect on fulvestrant-induced degradation (Fig. 2b). An alternative way to prevent recruitment of VHL to ERα is to knockout the VHL gene using CRISPR technology. Consistent with the competition studies, knockout of VHL partially reduced AZ’6421-induced ER degradation (Fig. 2c). Finally, we tested the less active, epimeric (*S,S,S*)-AHPC analogue **7** (Fig. 2d), which has drastically reduced binding to VHL as a result of inversion of the key hydroxyl group^27,28^. Despite AZ’6421 and compound **7** having similar ERα binding IC_50_ values (<0.6 nM and 1.0 nM, respectively) (Fig. 1a), compound **7** was unable to fully degrade ERα and resulted in a lower D_max_ of approx. 65% compared to 100% for AZ’6421 (Fig. 2e). Taken together these experiments suggest that whilst fulvestrant is not dependent on the VHL E3 ligase for degradation, AZ’6421 required the recruitment of VHL for maximal ERα degradation, as expected for a VHL-based PROTAC. We noted however that the PROTAC was still able to partially degrade ERα even when binding to VHL was compromised. We attributed this to the PROTAC carrying some innate “SERD-like” activity i.e. degradation that is induced by the binding of an ER ligand to ERα and not a result of VHL recruitment through the PROTAC mode of action. Such partial SERD-like activity is precedented and has for example been reported for ERα-binding analogues of AZD9833 and GDC-9545, showing that subtle changes can fundamentally change the activity of a compound^17,29^.

### AZ’6421 is a potent and effective degrader of ERα in multiple cell lines in vitro

**Fig. 3:**
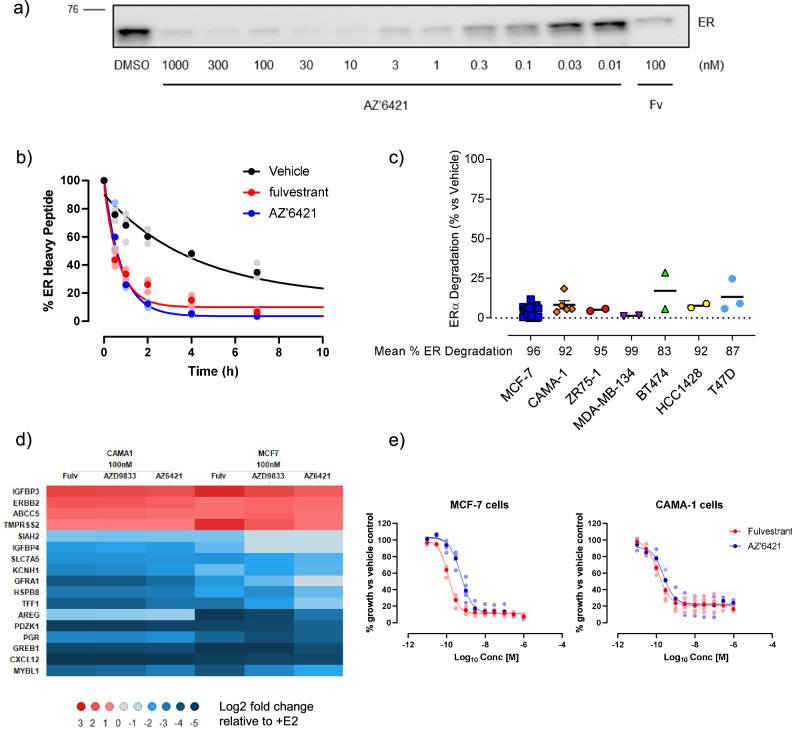
AZ’6421 potently degrades ERα and inhibits ER-regulated gene transcription. **a** MCF7 cells were treated with increasing concentrations of AZ’6421 for 24 h and probed for ERα by immunoblot analysis. **b** SILAC experiment to measure ERα peptide turnover in MCF7 cells. Cells cultured in the presence of heavy L-arginine were switched at time T = 0 to media containing light L-arginine plus 0.1% DMSO, 1 µM AZ’6421, or 1 µM fulvestrant. Cells were collected at the indicated timepoints and the proportion of heavy-labelled ERα assessed by mass spectrometry. % ERα heavy peptide is plotted relative to DMSO control. Data points show the mean of 3 independent experiments with points in bold representing the mean. **c** A panel of ER+ cells were treated with 100 nM AZ’6421 for 48 h. ERα was normalised to vinculin and expressed as % of vehicle control. Data points represent an independent experiment for each cell line, error bars indicate ± s.e.m. **d** Heatmap showing changes in mRNA expression of a panel of ER-regulated genes. MCF7 and CAMA1 cells were stimulated with 0.1 nM estradiol in the presence of DMSO, 100 nM fulvestrant (Fulv), 100 nM AZD9833, or 100 nM AZ’6421 for 24 hours. Data points show 3 independent experiments. **e** Growth inhibition curves for MCF7 and CAMA1 cells, treated with increasing concentrations of AZ’6421 for 6 days. Data points show 4–5 independent experiments, bold points represent the mean.

We next evaluated the dose and time dependence of ERα degradation by AZ’6421 in the MCF7 ER+ breast cancer cell line. Cells were treated for 24 hours with AZ’6421 and ERα levels were assessed by western blot. AZ’6421 reduced ERα protein expression in a dose-dependent manner, with maximal degradation achieved at concentrations of 10 nM and above (Fig. 3a), consistent with the data obtained in our high-throughput imaging assay (Fig. 2e). To explore the kinetics of degradation a pulse-chase experiment, using stable isotope labelling with amino acids in cell culture (SILAC) followed by mass spectrometry, was performed to track ERα protein levels over time (Fig. 3b). Addition of 1 µM AZ’6421 increased the rate of degradation of the existing, heavy isotope-labelled ERα, compared to vehicle control, with the half-life of ERα decreasing from 2.9 ± 0.2 hours in the presence of DMSO to 0.5 ± 0.9 hours in the presence of 1 µM AZ’6421. We further assessed the ability of 100 nM AZ’6421 to reduce ERα levels in a panel of ER+ breast and endometrial cancer cell lines. In all cell lines tested, AZ’6421 reduced ERα protein abundance to less than 20% of the vehicle, demonstrating that AZ’6421 was an effective ERα degrader in multiple cell lines (Fig. 3c).

To confirm that AZ’6421 treatment affected ER-regulated gene expression we assessed the expression of a panel of ER-regulated genes by qPCR. MCF7 and CAMA1 cells were starved of serum for 24 hours and then treated with 0.1 nM estradiol for a further 24 hours in the presence of DMSO, 100 nM AZ’6421, 100 nM AZD9833 or 100 nM fulvestrant. As shown in the heatmap (Fig. 3d), AZ’6421 effectively supressed the expression of a range of ER-regulated genes to a similar extent as fulvestrant and next-generation SERD AZD9833. Conversely, those genes that were inhibited by estradiol were induced by all three ERα degraders. Consistent with its ability to inhibit ER signalling, AZ’6421 potently inhibited the growth of MCF7 and CAMA1 cells with an IC_50_ of 0.5 nM and 0.2 nM, respectively (Fig. 3e).

These data confirmed that AZ’6421 is a potent ER PROTAC that can effectively degrade ERα in vitro across multiple cell lines, leading to inhibition of gene transcription and reduced cell growth.

### Formulation improved the oral exposure of AZ’6421

**Fig. 4:**
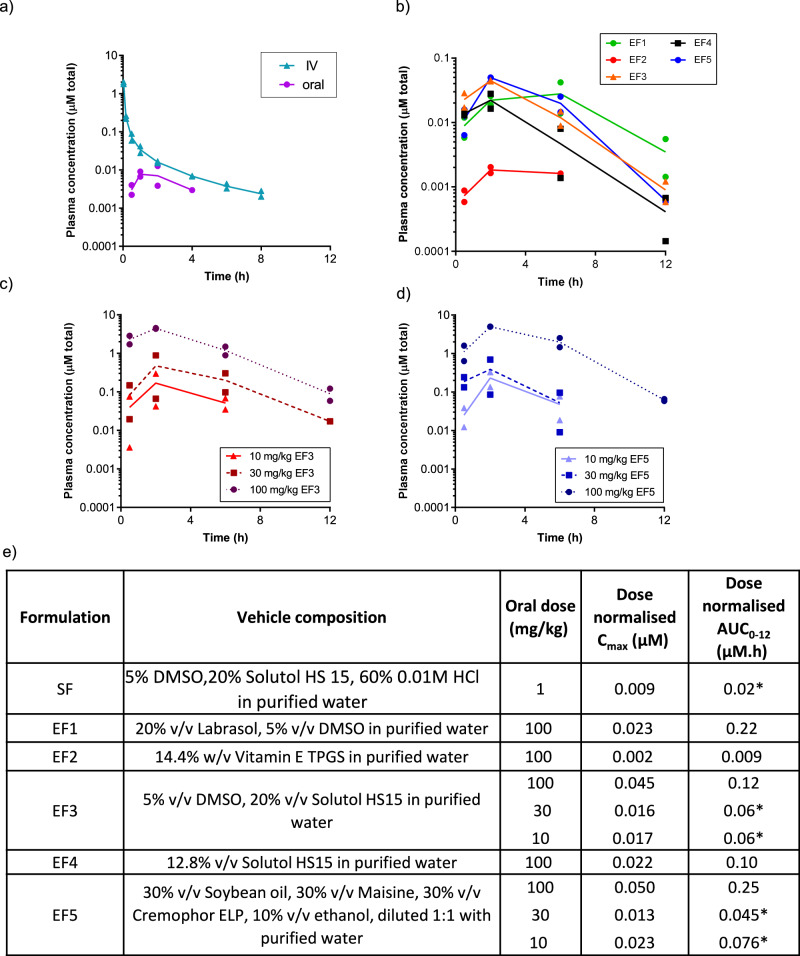
Oral exposure of AZ’6421 in enabling formulations. **a** PK experiment using 0.5 mg/kg AZ’6421 in 95% SBE-β-CD (30% w/v) in purified water as i.v. leg and 1 mg/kg AZ’6421 in SF as p.o. leg. **b** Plasma exposure of AZ’6421 at 100 mg/kg in a range of enabling formulations (EF) in mice. **c** Dose linearity investigation of EF3 showing 28% relative bioavailability at a 100 mg/kg dose in mice. **d** Dose linearity investigation of EF5 showing 33% relative bioavailability at a 100 mg/kg dose in mice. **e** Dose normalised exposure (C_max_ and AUC) of AZ’6421 in a range of enabling formulations. (*) AUC_0-6_ (data BLQ beyond 6 hours).

AZ’6421 in vitro was metabolically stable (mouse liver microsomes and hepatocytes, 22 µL/min/mg protein and 9 µL/min/x10^6^ cells, respectively), demonstrated low mouse in vivo clearance (22 mL/min/kg), and had an oral exposure (area under the concentration time curve (AUC)) of 0.02 µM.h following a 1 mg/kg dose (Fig. 4a). This exposure was achieved with a Solutol HS 15 solution formulation (SF; 5% DMSO,20% Solutol HS 15, 60% 0.01 M HCl in purified water) and was encouraging given that the physicochemical properties of AZ’6421 are beyond the rule of 5 property space^30–35^.

Enabling formulations (EF1 to EF5) were investigated to assess whether the oral exposure of AZ’6421 in mice could be increased (Fig. 4b–e) to enable PK/PD/efficacy understanding and confirm proof of mechanism. Self-emulsifying amphiphilic agents namely, Vitamin E TPGS, Labrasol, Solutol HS15, and Cremophor EL, were found to increase the solubility of AZ’6421 in pre-formulation screens and maintain supersaturation in biorelevant fluids used to mimic dilution and dispersion in the gastrointestinal tract (GIT). Furthermore, these solubilizers are reported to inhibit efflux transporters such as P-gp and potentially enhance drug absorption. DMSO and/or ethanol were included as water-miscible co-solvents in EF1, EF3, and EF5 to further aid dispersion and maintain solvent capacity in the GIT. Maisine (long-chain glycerides) and soybean oil were included in EF5 to promote chylomicron secretion and potentially increase bioavailability via intestinal lymphatic drug absorption.

EF3 and EF5 displayed maximum concentration (C_max_) and exposure (AUC) approximately 2-fold higher than all other formulations. Further investigations, including ascending doses of the two dosing formulations (EF3 and EF5), were completed (Fig. 4b, c). In mice, a dose linear increase in exposure (C_max_ and AUC) from 10 to 30 mg/kg (within 2-fold) and a supra-proportional increase in exposure from 30 to 100 mg/kg (>2-fold) was observed using EF5. In comparison, a dose linear increase in exposure from 10 to 100 mg/kg (within 2-fold) was observed using EF3. The relative bioavailability achieved following a 100 mg/kg dose was 28% with EF3 and 33% for EF5 (Fig. 4b, c). With dose linear increases in exposure up to 100 mg/kg, EF3 was selected as the dosing formulation for future studies.

### Pharmacodynamic studies highlighted sub-optimal degradation in vivo

To explore the ability of AZ’6421 to induce ERα degradation in vivo, a PD study was performed in the CTC174 patient-derived xenograft (PDX) model, using AZD9833 as a positive control. The CTC174 cell line harbours a D538G mutation close to the ligand binding domain mutation that makes ERα constitutively active, and tumours can therefore grow in the absence of estrogen^36^.

**Fig. 5:**
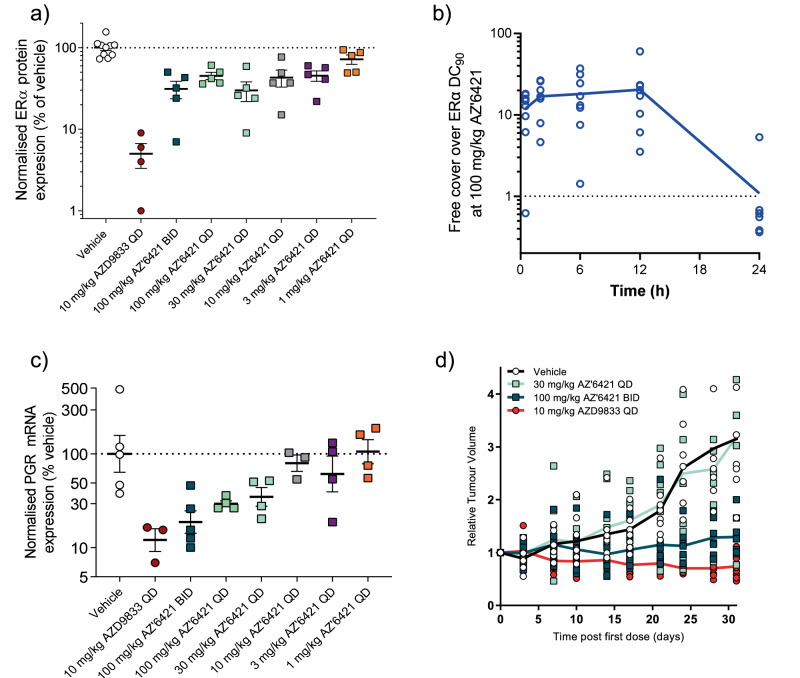
In vivo PK/PD/efficacy of AZ’6421 in CTC174 PDX model. **a**–**c** CTC174 xenografts grown in female NSG mice were dosed once or twice daily for 4 days with vehicle, AZ’6421, or AZD9833 at the doses shown. **a** Tumour tissues were harvested 6 h after the last dose for analysis of ERα protein levels by immunoblotting. ERα levels were normalised to vinculin control and expressed as a % of the mean vehicle control. **b** Free plasma exposure for AZ’6421 over the ERα DC_90_ achieved at 100 mg/kg. **c** Tumour tissues were harvested 6 hours after the last dose for analysis of PGR mRNA expression levels by qPCR. PGR expression was normalised to beta-actin control and expressed as a percentage (%) of the mean vehicle control. **d** CTC174 xenografts grown in female NSG mice were dosed once or twice daily with the compounds as shown. Tumour growth was measured by caliper at regular intervals, individual tumour volumes plotted and the mean indicated with lines for each dosed group. All error bars indicate ± s.e.m.

Increasing doses of AZ’6421 demonstrated a dose-dependent reduction in ERα levels, with approximately 70% degradation achieved at 30 mg/kg (Fig. 5a). However, further increasing the dose to 100 mg/kg, either once or twice daily, had no additional effect and the levels of ERα appeared to plateau at 70% ERα degradation. This was in contrast to the next-generation SERD, AZD9833, which resulted in approximately 90% degradation at 10 mg/kg, as has been consistently observed in previous studies^37^. We speculated that the 100 mg/kg dose of AZ’6421 had delivered insufficient exposure in the mouse to cause maximal, 90% ERα degradation. However, assessment of plasma drug concentration demonstrated that the 100 mg/kg dose achieved sufficient cover for target engagement (free plasma exposure > free DC_90_) (Fig. 5b), with measured free levels of 0.02% for AZ’6421 in mouse plasma (Table 2). This was further supported by two additional pieces of data. Firstly, assessment of tumour progesterone receptor (PGR) mRNA levels, a well-known ER-regulated gene, showed that PGR mRNA decreased in a dose-dependent manner across the entire dosing range. The 100 mg/kg BID dose achieved a greater reduction in PGR mRNA levels than the 30 mg/kg dose, and approached the extent of inhibition achieved with AZD9833 (approximately 80% inhibition *c.f*. 88% inhibition with AZD9833) (Fig. 5c). Secondly, in an efficacy study, 100 mg/kg BID of AZ’6421 resulted in 86% tumour growth inhibition whereas the lower dose of 30 mg/kg QD had no significant effect on tumour growth (Fig. 5d). Taken together, these data suggest that the lower than expected ERα degradation observed in vivo was not due to poor plasma exposure and a subsequent lack of ERα binding, since we still observed reduced PGR expression and tumour growth inhibition statistically equivalent to that achieved with 10 mg/kg AZD9833. Rather, the data suggest that the PROTAC degradation mechanism was perhaps compromised in vivo and therefore unable to reproduce the efficient ERα degradation achieved in vitro.

**Table. 2:** Binding, degradation, lipophilicity, and mouse fraction unbound in plasma

Compound	ERα binding IC_50_ (nM)^a^	ERα DC_50_ (nM)	ERα D_max_ (%)^b^	chrom LogD	Mouse fup
**AZ’6421 (5)**	<0.6 ± 0.2 (5)	0.4 ± 0.1 (8)	100 ± 1 (8)	6.6 ± 0.4 (3)	0.0002 (1)
**8**	<0.5 ± 0.0 (4)	0.5 ± 0.1 (4)	86 ± 4 (4)	3.8 (2)	0.005 (1)

a) Mixture of sub-nanomolar values and results below tight binding limit.

b) Maximum level of degradation observed for the compound where 0% is vehicle control and 100% is maximal concentration observed for fulvestrant. Data presented is the mean across replicates with standard deviation noted as the ± value. Numerical values in brackets are the number of replicates.

### Presence of metabolite resulted in reduced degradation in vivo

**Fig. 6:**
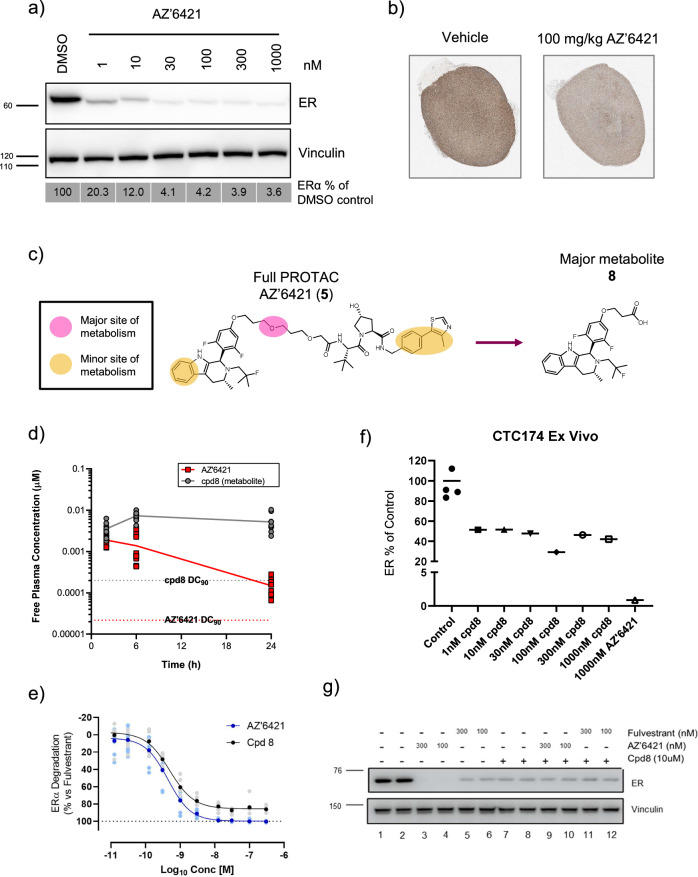
Assessment of the cause of the in vitro/in vivo disconnect. **a** CTC174 tumours were disaggregated into single cells, seeded in 6-well plates and dosed with increasing concentrations of AZ’6421 for 24 hours before being lysed. ERα levels were assessed by immunoblotting. ERɑ levels normalised to vinculin and expressed as % DMSO control are shown underneath the immunoblot. **b** Representative IHC images taken from sections of tumour treated with vehicle control or 100 mg/kg AZ’6421 for 4 days. Sections were stained with an antibody against ERα (Sp1, Ventana). **c** Schematic of potential sites of metabolism **d** Free plasma exposure of AZ’6421 and its metabolite **8** following 100 mg/kg dosing of AZ’6421. **e** Concentration-response curves for AZ’6421 and metabolite **8**. ERα levels in MCF7 cells were assessed using an immunofluorescence endpoint after 24 h exposure to compound. Individual data points were normalised to vehicle control (0% inhibition) and 100 nM fulvestrant (100% inhibition) and a concentration-response curve plotted using nonlinear regression analysis (*n* = 8–10). Points in bold represent the mean. **f** Effect of metabolite **8** on ERα degradation in CTC174 cells cultured ex vivo. Graph shows ERɑ levels normalised to vinculin and expressed as % DMSO control. **g** Immunoblots for ERα and vinculin loading control. MCF7 cells were pre-treated with 10 µM cpd **8** or DMSO control for 1 h before being exposed to DMSO, AZ’6421 or fulvestrant, at the concentrations indicated, for a further 24 h. A representative immunoblot from two biological replicates is shown.

**Fig. 7:**
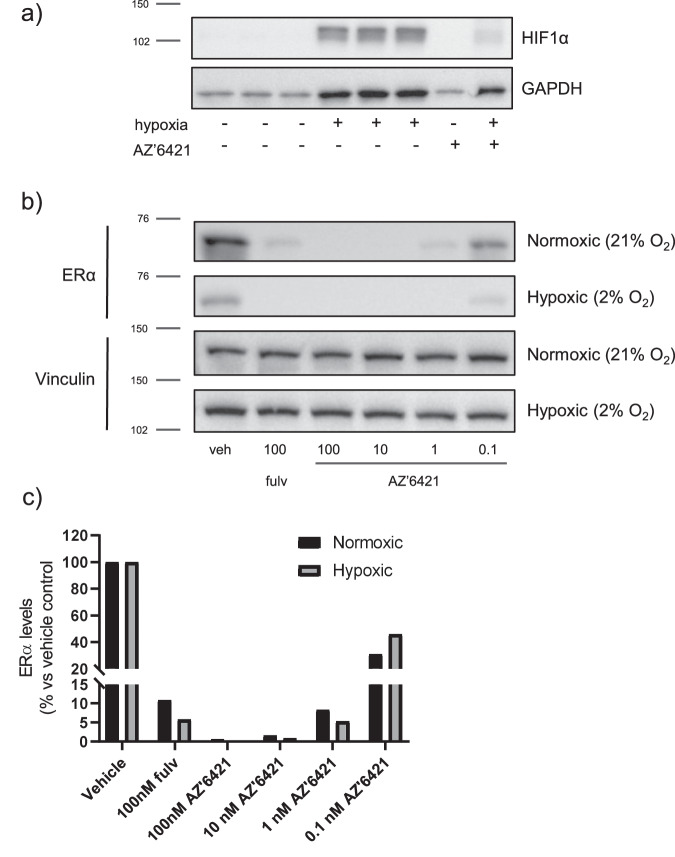
AZ’6421 degrades ERα under hypoxic conditions in vitro. **a** Immunoblots for HIF1α and GAPDH following incubation of MCF7 cells at 2% O_2_ within a Tri-gas incubator to induce hypoxia. **b** Effect of PROTAC and fulvestrant treatment on ERα degradation under normoxic and hypoxic conditions. MCF7 cells were exposed to normoxic or hypoxic conditions and treated with compound or 0.1% DMSO vehicle control for 48 h prior to being lysed. **c** ERα levels were normalised to vinculin and expressed as a percentage (%) of vehicle control. The data shown is representative of two separate experiments.

The data described above led us to believe that ERα degradation was limited by an alternative mechanism which resulted in a lack of complete degradation in the CTC174 PDX model in vivo. One hypothesis was that the VHL PROTAC was unable to achieve complete degradation in this model, perhaps due to low VHL levels, a phenomena that has been observed across different cell/tissue types^38^. We therefore established CTC174 cells ex vivo which allowed us to assess ERα degradation in an in vitro assay system. Incubation of CTC174 cells with increasing concentrations of AZ’6421 for 24 hours resulted in a dose-dependent reduction of ERα levels, with approximately 90% ERα degradation being achieved at doses of 10 nM and above (Fig. 6a). We next ruled out the possibility that ERα degradation was heterogeneous across the tumour, perhaps due to regions of hypoxia, which would reduce the extent of degradation observed in vivo. Sections of tumour were collected and immunohistochemistry analysis was performed to measure ERα levels. A 100 mg/kg dose of AZ’6421 caused a homogenous reduction in ERα levels across the tumour (Fig. 6b). We also tested the ability of AZ’6421 to degrade ERα under hypoxic conditions in vitro. Incubation of MCF7 cells for 48 hours at 2% O_2_ within a Tri-gas incubator caused a marked increase in expression of HIF1α and GAPDH, two known markers of hypoxia (Fig. 7a)^39^. Under these conditions, AZ’6421 was still able to effectively degrade ERα; 10 nM AZ’6421 resulted in 98% and 99% reduction in ERα levels under normoxic and hypoxic conditions, respectively (Fig. 7b, c).

Another hypothesis investigated was the potential for a metabolite of AZ’6421 to compete with the full PROTAC for binding to ERα. In an in vitro mouse hepatocyte metabolite identification study, oxidation of the gamma carbon and cleavage of the formed ester was found to be the major metabolic liability (Fig. 6c), resulting in the formation of a carboxylic acid metabolite **8**, with a chromLogD of 3.8 (*vs* 6.6 for AZ’6421). Determination of plasma protein binding of **8** in mouse plasma demonstrated 0.5% free compared to 0.02% free for AZ’6421 (Table 2). Compound **8** was subsequently quantified in the plasma samples from the AZ’6421 efficacy study and demonstrated free plasma concentrations ~10-fold higher than free AZ’6421 at steady state and >10-fold above the compounds DC_90_ (Fig. 6d). To better understand the impact of the metabolite on ERα degradation we synthesised metabolite **8** and profiled it across a panel of in vitro assays. The binding IC_50_s of compound **8** and AZ’6421 were found to be <0.6 nM, close to the tight binding limit of the assay. In the ERα degradation assay compound **8** had a DC_50_ of 0.5 nM, equivalent to that of AZ’6421 (0.4 nM), although the D_max_ was reduced to 86% compared to 100% for AZ’6421 (Fig. 6e and Table 2). These data suggest the metabolite binds efficiently to ERα but was unable to achieve levels of ERα degradation seen for AZ’6421 or fulvestrant. The reduced ERα degradation of the metabolite was further confirmed in CTC174 cells cultured ex vivo. Whilst 1000 nM AZ’6421 reduced ERα levels to less than 1% of control, compound **8** only decreased ERα levels to 42% of control when dosed up to 1000 nM (Fig. 6f). To understand the potential impact of the metabolite on the ability of the full PROTAC to degrade ERα, a competition experiment was performed. MCF7 cells were treated with DMSO, AZ’6421, or fulvestrant in the presence or absence of 10 µM **8**. Both the 100 and 300 nM dose of AZ’6421 demonstrated near-complete degradation of ERα as seen in previous experiments. However, the addition of an excess of **8** was able to reduce this degradation to levels achieved in the presence of **8** alone (Fig. 6g). Although the metabolite was presumably able to compete with fulvestrant for binding to ERα, the extent of degradation for both of these compounds alone was identical when dosed together (Fig. 6g, lanes 5-8).

**Fig. 8:**
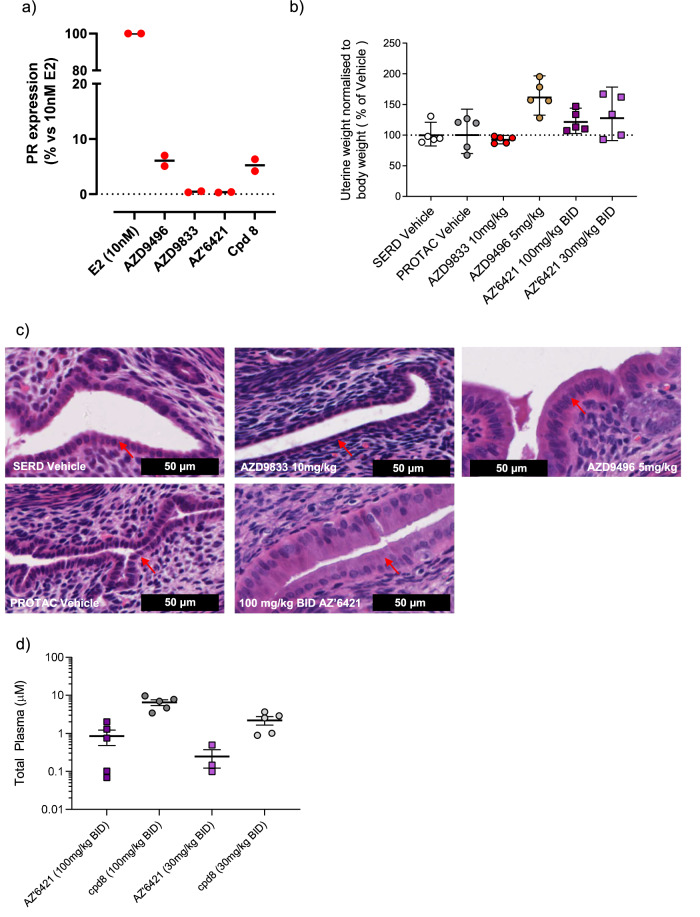
Evidence of ER agonism of AZ’6421 and compound 8 in vitro and in vivo. **a** 100 nM compound or 10 nM estradiol (E2) were incubated with Ishikawa cells for 48 h and lysates measured for PR expression by western blot. **b** Normalised rat uterine weight shown as % change from respective vehicle control after 4 days dosing. **c** Representative H&E stained images taken from sections of uterine horn after 4 days of dosing. **d** Total plasma exposure of AZ’6421 and its metabolite **8** following dosing of 100 mg/kg BID or 30 mg/kg BID AZ’6421. Plasma samples were taken 4 hr after the 4th dose, at the same time as the uterus was weighed and sectioned for H&E staining.

Some endocrine therapies, such as tamoxifen, have been reported to display weak agonistic activity in the endometrium due to altered cofactor recruitment and subsequent activation of gene transcription^40^. We therefore sought to test whether AZ’6421 was a complete antagonist or could act as a partial agonist, in both in vitro and in vivo models. In vitro, 100 nM AZ’6421, a concentration that causes maximal ERα degradation, failed to induce PR expression in the Ishikawa endometrial cell line (Fig. 8a) suggesting that this molecule was a complete antagonist. A similar result was also observed with 100 nM AZD9833, whereas AZD9496, a reported partial agonist in uterine tissue, induced PR expression, as expected^41^. In contrast, when we tested the agonistic properties of AZ’6421 in vivo, using the previously described immature female rat model^42^, we were surprised to find that AZ’6421 behaved more like AZD9496; both the 30 and 100 mg/kg doses of AZ’6421 showed a trend of increased uterine weight, similar to AZD9496 (Fig. 8b).

To further explore whether AZ’6421 may be acting as an agonist in vivo, morphological changes of the uterine were examined: uterine horns were formalin-fixed paraffin-embedded, stained with haematoxylin and eosin and then transverse sections of uterus examined. Rats treated with vehicle were observed to have small cuboidal epithelium, consistent with that of an immature rat uterus (Fig. 8c). Rats treated with AZD9833 were observed to have flattened/cuboidal epithelium, which is indicative of an anti-estrogenic like effect. In contrast, rats treated with AZD9496 were observed to have low columnar uterine epithelium (cells increased in size) with some mitotic figures, consistent with AZD9496 having an estrogenic (agonist) effect. Interestingly, rats treated with AZ’6421 also had increased cell size, with tall columnar uterine epithelium being observed; this taken alongside the increased uterine weight was supportive of AZ’6421 treatment causing an estrogenic (agonist) effect, in vivo.

These changes in endometrial histopathology observed with AZ’6421 were surprising due to the lack of agonism observed in vitro. However, given the mouse in vitro to in vivo disconnect described earlier, we hypothesised that the metabolite may also be contributing to the observed agonism. Similar to mouse, compound **8**, was found at sizable levels in rat plasma (quantified at the same time as the uterine horns) (Fig. 8d). To determine whether the metabolite could act as an agonist we explored PR induction in the Ishikawa cell line in vitro following compound **8** exposure. Unsurprisingly, compound **8** induced PR expression to a similar extent as AZD9496 (Fig. 8a), consistent with compound **8** being a partial agonist. These data led us to conclude that a circulating metabolite of AZ’6421, which is a poor degrader of ERα and an agonist, is the most likely explanation for the increased uterine weight and associated changes in endometrial histopathology observed in vivo.

## Discussion

PROTACs have received much attention over recent years as a new modality that has the potential to offer benefits over traditional small molecule inhibitors. Here we have highlighted an oral ERα-targeting PROTAC that recruits the VHL E3 ligase leading to potent and effective degradation of ERα in vitro.

We demonstrated that AZ’6421 is a potent degrader of ERα in vitro in multiple cell lines. The loss of ERα was rapid, with a half-life of 0.5 hours, and dependent on the proteasome, confirming that the reduction in ERα levels was due to degradation and not transcriptionally mediated. We also showed that degradation was occurring primarily through a PROTAC mechanism since recruitment of VHL was required to achieve maximal ERα degradation. The addition of excess acetylated-(*S,R,S*)-AHPC, VHL knockout or the use of a control AZ’6421 epimer with reduced binding to VHL all gave a reduction in D_max_ compared to AZ’6421 alone. This is in contrast to other reported PROTACs where, for example, loss of VHL recruitment led to complete rescue of AR or Brd4 degradation^43,44^. In both cases, the starting POI ligands, enzalutamide or JQ1, demonstrate no degradation activity. However, many molecules that bind to the ERα ligand binding domain have a SERD mechanism of action, and degrade ERα through a mechanism that is not fully understood but is thought to be a consequence of increased surface hydrophobicity through conformational changes in helix H12 in the ligand binding domain of ERα^45^. While we cannot rule out the possibility that the partial degradation was due to residual VHL recruitment through incomplete knockout of VHL or insufficient acetylated-(*S,R,S*)-AHPC to fully compete out the PROTAC, it was not unexpected that the PROTAC maintained some SERD-like degradation that was independent of VHL recruitment.

While mouse oral bioavailability was low for AZ’6421 using our standard formulation, it was possible to boost exposure of this PROTAC through the use of solubility-enabling formulations, allowing oral dosing in mouse (Fig. 4a–e). However, despite achieving in vivo exposures that were above the in vitro DC_90_ (Fig. 6d), AZ’6421 failed to achieve greater than 70% ERα degradation. The fact that we observed robust antagonism in CTC174 cells through reduced PGR mRNA expression and tumour growth inhibition at 100 mg/kg, approaching the levels achieved for AZD9833^37^, supported our hypothesis that sufficient compound was penetrating the tumour to bind and antagonise ERα. The ability of 100 mg/kg AZ’6421 to maintain good antagonistic activity in the absence of complete degradation is consistent with a previous finding where the low dose of fulvestrant that was unable to degrade ERα was still able to inhibit ER activity and block tumour growth^46^.

We methodically ruled out a series of biological explanations for the reduced degradation such as heterogenous degradation across the tumour and limited degradation capacity in the CTC174 PDX model and highlighted the importance of defining the metabolic profile of AZ’6421. Importantly, although the absolute levels of metabolite **8** were quite low, this molecule is far less lipophilic than the parent PROTAC and therefore present in substantial free quantities in vivo. Based on the binding potencies of AZ’6421 and metabolite **8** being approximately equal, and the difference in the free levels being 25-fold, only 4% of AZ’6421 needs to be metabolised to **8** for 1:1 binding to ERɑ. Examining Fig. 6d we can determine that 7% of AZ’6421 was converted to **8** within 2 hours, resulting in only approximately one-third of the ERɑ receptors being occupied by AZ’6421. The out competition of AZ’6421 is more pronounced over time as **8** has a longer half-life than the parent. However, the loss of ERα degradation was not complete, because as shown in vitro, both in MCF7 and the disaggregated CTC174 cells, compound **8** demonstrated partial SERD activity. These data agree with previous findings that using a metabolically stable small molecule as POI does not correspond to a metabolically stable PROTAC^47^, and suggest PROTACs and specifically their linkers need to be optimised for metabolic stability. This is critically important for PROTACs where metabolites of the bifunctional compound can compete for binding with the PROTAC at the POI, often at much higher free levels compared to the parent PROTAC, resulting in reduced degradation. The effect on efficacy may be minimal if the metabolite is still able to bind and effectively inhibit the POI or indeed, as is the case here, if the metabolite is still able to degrade through a non-PROTAC-based mechanism. However, the impact on overall efficacy may be substantially greater as we begin to develop PROTACs from novel, non-functional ligands where degradation is required to induce downstream pathway inhibition. In addition, metabolites may exhibit their own pharmacology, which may be particularly relevant for antagonists where small changes in the molecule may give rise to agonism^48^. Here we show AZ’6421 is a complete antagonist and causes no agonism in the Ishikawa endometrial cell line in vitro. However, metabolite **8**, which lacks the VHL binding moiety due to cleavage of the linker, is a lesser degrader of ERɑ and acts as an agonist in Ishikawa cells. Although we have not confirmed conclusively that the agonism observed in vivo in the rat uterine model is due to the metabolite, the fact that we detected **8** at high levels in the plasma, showed it was able to compete for binding to ERɑ and is an agonist in vitro is strongly supportive of our hypothesis.

The metabolism of PROTACs is an active topic and the metabolism of AZ’6421 matches with the in vitro findings by Goracci et al., with metabolites forming adjacent to heteroatoms in the PROTAC linker^49^. We identified a carboxylic acid **8** formed from oxidation adjacent to ether in the linker in both the metabolite identification studies in vitro and from the in vivo PDX study. The inclusion of cyclic moieties has been mooted as a strategy to increase metabolic stability^49^. However, we found the inclusion of rings in the linker usually resulted in a loss of ERɑ degradation activity (Table 1 and Table S4). While piperazine containing **6** did match AZ’6421 in our imaging degradation assay, it did not degrade ERα to a sufficient extent in the nanoluciferase degradation assay and showed no improvement in clearance in mouse hepatocytes. We believe that including cycles in the linker rigidifies the linker and precludes the compound from adopting a conformation similar enough to the suggested bioactive conformation (Fig. 1b).

In conclusion, we have shown that AZ’6421 is a potent degrader of ERα in vitro but highlight a disconnect when transitioning to an in vivo setting driven by the presence of a metabolite still capable of binding potently to the POI and expect this observation to be relevant in wider PROTAC development. We believe that optimising PROTACs for metabolic stability and carefully defining their metabolic profile in vivo will be essential to ensure the full potential of PROTACs is reached in the clinic.

## Experimental methods

### Chemical information and simulation

The methodology used for the synthesis of compounds described in this paper is available in Supplementary Note 3.

### Cell lines and reagents

All cell lines were obtained from the American Type Culture Collection (ATCC). Cells were cultured in RPMI1640 (phenol red-free, Sigma) supplemented with 5% foetal bovine serum (FBS, Gibco) and 2 mmol/L glutamine (Gibco) and maintained at 37 °C in a humidified chamber in the presence of 5% CO_2_. All cell lines tested negative for Mycoplasma and were authenticated by short tandem repeat analysis at the time of banking and used for a maximum of 15 cell passages. Compounds and fulvestrant obtained from AstraZeneca compound collection were dissolved in DMSO to a concentration of 10 mmol/L and stored under nitrogen. Antibodies for immunoblotting; anti-ER (clone SP1, Thermo Scientific), anti-progesterone receptor (PR, clone 636, Dako), anti-HIF1α (BD bioscience #610958), anti-Vinculin (1:5000, Sigma V9131) and anti-beta-actin (CST, 4970).

### Binding assays

The ability of compounds to bind to isolated ERα Ligand binding domain (ERα–LBD (GST)) was assessed in competition assays using a LanthaScreenTM Time-Resolved Fluorescence Resonance Energy Transfer (TR-FRET) detection end-point. For the LanthaScreen TR-FRET endpoint, a suitable fluorophore (Fluormone ES2, Product code P2645) and recombinant human ERα ligand binding domain (LBD) (product code PV4543) were purchased from Invitrogen and used to measure compound binding. A terbium-labelled anti-GST antibody (Product code PV3551) is used to indirectly label the receptor by binding to its GST tag, and competitive binding is detected by a test compounds’ ability to displace the fluorescent ligand resulting in a loss of TR-FRET signal between the Tb-anti-GST antibody and the tracer. The assay was performed with all reagent additions carried out using the Beckman Coulter BioRAPTR FRD microfluidic workstation:

### ERα high throughput imaging degradation assay

MCF7 cells were seeded into 384-well plates at 2000 cells/well in phenol red-free Dulbecco’s Modified Eagle Medium (DMEM) containing 5% (v/v) charcoal-stripped foetal bovine serum (FBS). The compound was incubated for 20 to 24 hours at concentrations ranging from 0.3 pM to 3 µM (37 ^o^C, 5% CO_2_). Cells were fixed, permeabilised and stained with anti-ERα (rabbit clone SP1, Thermo) and anti-PR (mouse clone PgR 636, Dako) antibodies followed by secondary AlexaFluor 594 goat anti-rabbit and AlexaFluor 488 goat anti-mouse IgG antibodies. Imaging was using the Thermo Cellinsight scanner and average fluorescence intensity was analysed using Genedata. Wells containing vehicle or 100 nM fulvestrant were used to define the minimum and maximum downregulation/inhibition respectively.

### Western blot analysis

Cells were seeded into 12-well tissue culture-treated plates at 0.5 x 10^6^ cells/well in phenol red-free RPMI containing 5% (v/v) charcoal-stripped FBS (F6765, Sigma) and left to adhere overnight. In experiments to detect agonism or for SILAC experiments, FBS was double stripped using activated charcoal. Cells were treated with compound or 0.1% DMSO vehicle control for the times indicated prior to being lysed in Pierce RIPA buffer (Thermo Scientific), supplemented with protease and phosphatase inhibitors. Expression levels of proteins were determined by standard western blotting techniques (BioRad 4%-12% Bis-Tris gels). Antibodies were diluted in 5% milk-PBS-Tween and protein levels were detected using Pierce WestDura chemiluminescent reagents followed by visualisation and quantification on a Syngene ChemiGenius Imager. Where blots were quantified, protein levels were normalised to beta-actin or vinculin control and expressed as % of vehicle control.

### CRISPR knock out

Chemically synthesised Edit-R crRNAs targeting VHL and ESR1 were purchased from Horizon Discovery (Cambridge, UK). Equal volumes of 4 crRNAs targeting each individual gene were pooled and then duplexed at a 1:1 ratio in 10 mM Tris pH7.4 buffer with tracrRNA (Horizon, U-002000-50) at room temperature. MCF7 cells were engineered to express cas9 under TetON promotor from the endogenous AAVS1 locus as described previously^50^. Doxycycline-inducible Cas9 is linked via the cleavable T2A peptide to BFP enabling the identification of Cas9-positive cells by microscopy. MCF7 cas9 cells were cultured in RPMI phenol red free (Sigma) supplemented with 10% FBS and 1X GlutaMax (both Gibco). 72 h prior to reverse transfection, cas9 expression was induced by the addition 15 ng/mL doxycycline (Sigma) to the growth media.

MCF7 cells were reverse transfected with the crRNA:tracrRNA pools at 25 nM final concentration using Lipofectamine® RNAiMAX transfection reagent (Thermo Fisher): First crRNA:tracrRNA pools were dispensed into 384-well assay plates (Greiner, 781090), and incubated with 10 µL serum-free DMEM (Sigma) supplemented with 1% Lipofectamine RNAiMAX for 20 minutes at room temperature. Then 6,000 cells were seeded into each well in 30 µL assay media (Phenol red-free DMEM supplemented with 5% charcoal-stripped FBS 1x Glutamax, 100U penicillin, and 100 µg streptomycin, and 15 ng/mL Doxycycline). 72 h post-transfection cells were treated with increasing concentrations of AZ’6421 for 24 hours before being fixed with formaldehyde (Sigma, 3.7% final concentration) and stained for ERα (SP1, Thermo Fisher, 1:750 dilution). Nuclei were counterstained with DRAQ5 (1:2000 dilution) and cells were imaged on the Cell Voyager 7000 (Yokogawa) using a 20x long working distance objective as described previously^51^. Following image acquisition, analysis was completed using the Columbus Image Analysis platform (Perkin Elmer) to quantify ESR1 levels in cas9-positive cells. The ESR1 levels were normalized to unedited cells (0) and ESR1 KO cells (-100) and plotted in Prism (GraphPad).

### Stable isotope labeling of amino acids (SILAC) and mass spectrometry

MCF7 cells were grown for a minimum of 2 passages in SILAC media (Pierce, Thermo Scientific) containing ^13^C_6_^15^N_4_ arginine and ^13^C_6_ lysine. The extent of stable isotope incorporation was determined by mass spectrometry. For the assay, cells were plated at 1.2 x 10^6^ cells/well into 6-well TC-treated plates in media containing 5% double charcoal stripped FBS^13^,C_6_^15^N_4_ arginine and ^13^C_6_^15^N_2_ lysine for 24 hours. At time 0, the media containing isotopically heavy arginine and lysine was replaced with media containing unlabelled equivalents plus either AZ’6421, fulvestrant or DMSO control. Samples were collected and lysed over a 24 hour period. Equal concentrations of timecourse lysates were mixed with an internal standard lysate derived from ^13^C_6_^14^N_2_ lysine labelled MCF7 cells. ER protein was immunoprecipitated overnight at 4 °C with an ER antibody (Rabbit clone SP1, Thermo Scientific) and immobilised onto a U-bottomed Nunc MaxiSorp plate (Thermo Scientific). Wells were sequentially washed with each of the following; RIPA buffer, PBS and water. Samples were digested by addition of 30 μL well sequencing grade trypsin (Promega) in 50 mM ammonium bicarbonate at 37 °C for a minimum of 16 hours. The resultant peptide samples were acidified and analysed by selected reaction monitoring (SRM) mass spectrometry. Protein half-life was determined using the one-phase exponential decay function in GraphPad PRISM where Y = (Y0 - Plateau)*exp(-K*X) + Plateau.

### Gene expression analysis

MCF7 and CAMA1 cells were plated at 1.2 x 10^6^ cells/well into 6-well TC-treated plates in phenol red-free RPMI containing 5% charcoal-stripped FBS (F6765, Sigma) and treated with 0.1 nM estradiol in the presence of 100 nM fulvestrant, 100 nM AZD9833, 100 nM AZ’6421 or DMSO control for 24 hours. A non-estradiol-treated control was also included with 3 biological replicates per condition. Samples were harvested in 1 mL of QIAzol Lysis Buffer and snap-frozen. RNA was extracted using the RNeasy 96 QIAcube HT total RNA Cell with DNAse treatment following manufacturer instructions. Samples were randomized over 96-well plates. RNA concentration was quantified using the Qubit RNA BR kit (# Q10213 Invitrogen) and RNA integrity was analyzed on a bioanalyzer using the RNA 6000 Nano Kit (# 5067-1511-Agilent) following the manufacturer’s instructions. Targeted gene expression was performed using a 48 × 48 or 96 × 96 Fluidigm dynamic array (Fluidigm, San Francisco CA, USA) and Taqman primers (Thermo Scientific, Waltham, MA). Following the manufacturer’s instructions, 50 ng of total RNA was reverse transcribed using a high-capacity complementary DNA (cDNA) reverse transcription kit (Thermo Scientific) and pre-amplified with a Taqman PreAmp master mix (Thermo Scientific) for 14 cycles with 45 selected ER target gene primers. The Fluidigm Array was then primed and loaded on an IFC Controller and quantitative PCR (qPCR) experiments were run on the Biomark System, using the standard Default_10 min_HotStart protocol or M96_default protocol for 48 × 48 or 96 × 96 chips, respectively. Data were collected and analysed using the Fluidigm Real-Time PCR Analysis software to generate the Ct values. Gene expression calculations were performed in Jmp®12.0.1, and data was represented in TIBCOTM Spotfire® 6.5.2. The Ct values of target genes were normalised to the average of housekeeping genes. The expression of each individual gene in each drug treatment group was then normalised to its respective expression in the DMSO control group to calculate log2 fold change in gene expression (negddCt):

### Cell proliferation assay

Cell proliferation was assessed by measuring live cell numbers using the Sytox Green assay. MCF7 and CAMA1 cells were seeded in 96-well clear-bottom black microplates (Costar) at 4x10^3^ and 8x10^3^ cells per well, respectively, based on the doubling time of each cell line. Plates were incubated at 37 °C, 5% CO_2_ for 24 hours prior to being treated with DMSO, AZ’6421 or fulvestrant in a 10-point concentration range, with a final DMSO concentration of 0.1%. A single plate was left untreated, designated a Day 0 plate. The number of live cells was determined at Day 0 and Day 7 using the Sytox green assay. Briefly, 5 mM Sytox green nucleic acid dye (Invitrogen) was diluted 1:2500 in 500 mM tris-buffered saline (TBS) with ethylenediaminetetraacetic acid (EDTA) and 7 μL was added per well. Plates were then incubated in the dark at room temperature for 1 hour and the number of green cells in each well (dead cells) was measured using an Acumen Explorer high-throughput cell imager (TTP Labtech). Next, 14 μL of 0.25% w/v Saponin in TBS/EDTA solution was added overnight to permeabilize the cells, permitting a total cell count. The number of live cells was calculated by subtracting the dead cell count from the total cell count. The data obtained were then used to perform curve fitting analysis using GraphPad Prism. Average cell counts from the Day 0 plate were used to determine 0% cell growth. A dose-response curve was plotted using non-linear regression analysis to determine the half-maximal inhibitory concentration (IC_50_).

### Hypoxia studies

MCF7 cells were seeded into 12-well tissue culture-treated plates at 0.5x10^6^ cells/well in phenol red-free RPMI media containing 5% (volume/volume [v/v]) charcoal-stripped FBS (F6765, Sigma) and left to adhere overnight. The hypoxia-treated plates were transferred to a Thermo Heracell 150 Tri-gas Incubator set at 2% O2 and allowed to equilibrate to the lower oxygen level overnight. Cells were treated with compound or 0.1% DMSO vehicle control for 48 hours prior to being lysed in Pierce RIPA buffer (Thermo Scientific), supplemented with protease and phosphatase inhibitors. Lysis was carried out immediately after removal from the Tri-gas incubator to prevent the rapid degradation of HIF1α on re-exposure to the higher oxygen levels. Protein expression was determined by standard western blotting procedures as described above.

### In vivo xenograft studies

CTC174 (ER/PR positive, HER2-negative patient-derived xenograft (PDX) model) studies were conducted following implantation of a tumour fragment in female NSG mice; established tumours were removed from donor mice and dissected into 3x3x3 mm pieces in RPMI 1640 media. Female NOD scid gamma (NSG) mice were anesthetized using 2% isoflurane, a small incision was made through the skin, 0.5 cm dorsally to the third nipple, and a tumour fragment was inserted between the skin layer and the third mammary fat pad. The skin was closed with surgical glue (VetBond, 3 M).

### PD studies

Mice were randomised into groups of 5 once tumour sizes reached approximately 0.3 to 0.4 cm^3^. A vehicle control or AZ’6421 1, 3, 10, 30, and 100 mg/kg were dosed orally, once daily (QD) or twice daily (BID) in 5% v/v DMSO, 20% v/v Solutol HS15 in purified water formulation. AZD9833 was dosed once daily at 100 mg/kg in 40% TEG/60% (of 12.5%) SBE-β-CD formulation. Mice were dosed for 4 days and tumours were harvested 6 hours after the last dose. Tumour tissue was split into 3 and immediately snap-frozen in liquid nitrogen for subsequent analysis. Plasma samples were also taken at the end of the study by terminal cardiac bleed under isoflurane anaesthesia to measure pharmacokinetics and ensure the expected exposure was reached during the dosing period.

To determine levels of ERα tumours were lysed in Invitrogen Cell Extraction buffer (FNN0011) with added Sigma Phosphatase inhibitors (No.2 [P5726] and No.3 [P0044]; 1:100 dilution), protease inhibitor cocktail (P8340; 1:200 dilution) and Roche complete protease inhibitor (11836145001; 1 tablet per 50 mL). Samples were homogenized for 20 seconds using a T25 Ultra-Turrax homogenizer and then centrifuged at 13,000 rpm for 15 minutes. Approximately 45 μg of protein was run on a 4–12% bis-tris gel using standard methods and ER and beta-actin levels were assessed by immunoblotting as described above. Statistical analysis was performed on values normalized to beta-actin using a one-tailed, unequal variance t-test compared with vehicle control. Individual animal percentage inhibitions were calculated by the following equation:

Individual animal percentage inhibition = ((vehicle control geometic mean – individual animal expression)/vehicle control geometric mean)) × 100%.

To determine levels of PR mRNA, snap-frozen tumours were homogenized and resuspended in QIAzol Lysis Buffer. RNA was extracted using the RNeasy 96 QIAcube HT total RNA Cell with DNAse treatment following the manufacturer’s instructions. Samples were randomized over 96-well plates. RNA concentration was quantified using the Qubit RNA BR kit (# Q10213 Invitrogen) following the manufacturer’s instructions. RNA integrity was analyzed on a bioanalyzer using the RNA 6000 Nano Kit (# 5067-1511-Agilent) following the manufacturer’s instructions.

### Efficacy studies

Mice were randomised into groups of 8-10 once tumour sizes reached approximately 0.2 cm^3^. AZ’6421 was dosed orally at 30 mg/kg QD or 100 mg/kg BID using EF3, alongside a vehicle control. AZD9833 was dosed at 10 mg/kg PO, QD. Tumours were measured twice per week and changes in tumour volume and growth inhibition were determined by bilateral Vernier caliper measurement (length x width), where length was taken to be the longest diameter across the tumour and width the corresponding perpendicular. Tumour volume was calculated using the formula (length x width) x √ (length x width) x π/6). Tumour growth inhibition from the start of the study to the final day of tumour measurement was assessed by comparison of the geometric mean change in tumour volume for the control and treated groups. Tumour regression was calculated as the percentage reduction in tumour volume from baseline (pre-treatment) value (where relative tumour volume [RTV] = geometric mean RTV):

$$\% \,regression=(1-RTV)\times 100\, \%$$

Statistical significance was evaluated using a one-tailed t-test compared to vehicle control at the day of final tumour measures.

### CTC174 ex vivo studies

CTC174 tumours were collected from donor mice, all fat, mouse tissue and necrotic areas removed and the resultant tumour placed in ice cold PBS and kept on ice. Using forceps, the tumour was then minced into small pieces (1-2 mm). Once minced, the tissue was colleced, placed into a gentlMACS C tube with 4.7 mL DMEM/F12 and enzymes H, R and A (Miltenyi Tumour Dissociation Kit), and run on the gentleMACS Octo Dissociator (Program 37C_h_TDK_2). After dissociation, the cell suspension was passed through a 70 µM filter, red blood cell lysis performed using AKB lysis buffer, and the resultant suspension centrifuged, resuspended in RPMI (containing 10% charcoal-stripped FCS) and 1x10^6^ cell added to each well of a 6 well plate. DMSO control, AZ’3372 or AZ’6421 were added to well at a range of concentrations, left to incubate for 24 hours (37^o^C, 5% CO_2_) and then samples prepared for western blot and ERα levels determined as described above.

### IHC methods

Immunohistochemistry was used to evaluate the PD effects induced by increasing doses of AZ’6421 in CTC174 patient-derived xenograft (PDX) model study when compared to untreated and AZD9833 (10 mg/kg) treated tumours. The PDX xenograft tumours were formalin-fixed paraffin-embedded and sectioned at 4 µm. ER expression in tumours was assessed by IHC validated assays using the antibody clone SP1 (Ventana, Tucson, AZ) and detected using the UltraView Universal DAB detection on the Benchmark Ultra (Ventana, Tucson, AZ). Whole digital slide images were obtained using virtual microscopy (Aperio AT2, Leica Biosystems).

### Metabolite identification

CD-1 male mouse hepatocytes (BioreclamationIVT, M005052, lot:LRH) were thawed and warmed to 37 °C using Lebovitz’s L-15 medium and Cell yield/cell density and viability were assessed by a Nexcelom cell counter.

Incubations were conducted on a Hamilton robot, 0.25 mL of the hepatocyte suspension (1 million cells/mL) was transferred for each incubation into Nunc 96-deepwell plate. Substrate (2.5 µL of 0.5 mM in acetonitrile) was added (final concentration of 5 µM) and then incubated for 60 minutes at 37 °C. The samples were quenched with an equal volume of acetonitrile, spun at 3500 rpm for 10 minutes and 0.2 mL of the supernatant was transferred into 0.6 mL of water for analysis by UHPLC-UV-HRAMS.

### Plasma Bioanalysis

Each plasma sample (25 mL) was prepared using an appropriate dilution factor, and compared against an 11-point standard calibration curve (1-10000 nM) prepared in DMSO and spiked into blank plasma. Acetonitrile (100 mL) was added with the internal standard, followed by centrifugation at 3000 rpm for 10 minutes. The supernatant (50 mL) was then diluted in 200 mL acetonitrile:water (75:25) and analyzed via UPLC-MS/MS.

#### UPLC-MS/MS analysis

The MS/MS instrument used was either a Waters XEVO® TQ-S, Waters XEVO® TQ-D, or API 4000 (AB Sciex). The ultra-mass spectrometer used for sample analysis was completed in the MRM mode (MS/MS). Reverse-phase HPLC with a C18 column was used to separate the analytes. A mobile phase of 99% water/0.1% formic acid (solvent A) and a solvent phase of 99% acetonitrile/0.1% formic acid (solvent B) was used. A generic LC gradient elution was used at a flow rate of 0.5 mL/min with 95% solvent A and 5% solvent B for 0.3 min after which the concentration of solvent B was increased to 95% over 0.9 min before restoring it back to 5% for the remaining 0.5 min. Mass spectrometer methods were optimised for each compound.

For all assays, the 100% ice-cold acetonitrile used to quench the samples contained internal standards to ensure efficient extraction of sample, confirm injection into the mass spectrometer, and allow assessment of ionisation variability. Data was accepted if the internal standard peak area coefficient of variation was <20%.

### Chromatographic LogD

An HPLC with UV detection using diode array detection over a wavelength range of 210-320 nm and confirmation by electrospray mass spectrometry (pos/neg ionisation) is used to determine the chromatographic LogD of compounds. Test compounds, calibrants, and QC samples are prepared as 10 mM stocks in DMSO and further diluted to a final concentration of 0.6 mM in DMSO. A Waters Acquity BEH C18, 1.7 µm, 50 x 2.1 mm column is used for the analysis with a mobile phase A of 10 mM ammonium acetate in 5/95 CH3CN/H2O adjusted to pH 7.4 with ammonia and mobile phase B composed of 10 mM ammonium acetate in 95/5 CH3CN/H2O pH adjusted with the same volume of ammonia used to adjust mobile phase A. HPLC Gradient condition is as follows: 0.1% B during 0 to 0.17 min, a linear increase to 99.9% B during 0.17 to 1.5 min, hold at 99.9% B during 1.5 to 1.8 min then back to 0.1% B from 1.81 to 2.2 min with a flow rate of 1.0 mL min^-1^, UV detector λ 220-320 nm and injection volume of 2 µL. The chromatographic system is calibrated using standards with known literature values of octanol-water partition coefficient at pH 7.4 (logD7.4) and retention factors, k’, of calibrants, QCs, and samples calculated using equation: k’=(rt-t0)/t0.

### Plasma protein binding

Plasma protein binding was completed using a RED device. The test compound was prepared to 1 mM in 100% DMSO and further diluted in plasma to achieve a final compound concentration of 5 μM in the incubation. Phosphate buffered solution pH 7.4 (500 μL) was added to the receiver chamber of the RED device and spiked plasma (300 μL) was added to the donor chamber. The plate was covered with a gas-permeable lid and incubated for 18 h at 37 °C with 5% CO_2_ on an orbital shaker at 300 rpm. At the end of incubation, 50 μL of post-dialysis sample from the donor and receiver wells were aliquot into separate wells and matrix-matched with 50 μL of phosphate buffer solution pH 7.4 or blank plasma, respectively. The samples were subsequently quenched separately in 400 μL of ice-cold 100% acetonitrile. Quenched samples were shaken at 1000 rpm for 10 min and centrifuged for 30 min at 4000 rpm to pellet precipitated protein. The supernatant fraction was further diluted 1:1 with deionised water for analysis by LC MS/MS^17^.

### Mouse hepatocyte intrinsic clearance

Livers from CD-1 male mice weighing 30-35 g were digested and perfused using D-Hanks solution containing EDTA or collagenase. Once digested the liver was transferred to a petri dish and the hepatocytes were prepared by washing and centrifuging with a Leibovitz-15 medium containing 50% isotonic percoll. A cell viability >80% was required to progress the cells. The test compound was prepared to 10 mM in 100% DMSO and further diluted to 100 μM in 100% acetonitrile. The hepatocyte incubations were prepared in Leibovitz’s L-15 Medium pH 7.4 containing 1 million hepatocytes/mL and a final compound concentration of 1 µM. Cell viability was determined using a Cellometer Vision and >80% cell viability was required to proceed with the compound incubation. The compound/cell solution (250 µL) was incubated for 2 h at 37 oC and shaken at 900 rpm on an Eppendorf Thermomixer Comfort plate shaker. Samples (20 µL) were taken at 0.5, 5, 15, 30, 45, 60, 80, 100, and 120 min and quenched with 100 μL of 100% ice-cold acetonitrile. Samples were shaken at 800 rpm for 2 min and centrifuged at 4000 rpm for 20 min at 4 °C to pellet precipitated protein. The supernatant fraction was diluted 1:5 with deionised water, shaken at 1000 rpm for 2 min, and further diluted 1:1 with deionised water. Samples were analysed by LC-MS/MS^17^.

### Determination of mouse microsome intrinsic clearance

Mouse liver microsomes (Male CD-1) are obtained from Corning at a concentration of 20 mg/mL protein. The test compound was prepared to 10 mM in 100% DMSO and further diluted to 100 μM in 100% acetonitrile. The microsomal incubations were prepared in phosphate buffered solution pH 7.4 containing 1 mg/mL microsomal protein, 1 mM NADPH and a final compound concentration of 1 μM. Following a pre-incubation with NADPH for 8 min, reactions were initiated through the addition of the test compound (final volume 250 µL) and incubated at 37 °C in a water bath for 30 min. At each time point (0.5, 5, 10, 15, 20, 30 min) 20 μL of incubation mixture was quenched with 100 μL of 100% ice cold acetonitrile. Samples were shaken at 800 rpm for 2 min and centrifuged at 4000 rpm for 20 min at 4 °C to pellet precipitated protein. The supernatant fraction was diluted 1:5 with deionised water, shaken at 1000 rpm for 2 min, and further diluted 1:1 with deionised water. Samples were analysed by LC-MS/MS^17^.

### ERα nanoluciferase degradation assay

MCF7 pTRIPZ Cas9 cells stably transfected with pSMPNeo dual Luc ESR1 vector (containing ESR1-Nanoluciferase fusion protein/reporter and a Firefly luciferase reporter) were seeded into T175 flasks and incubated for 48 hours in RPMI 1640 media containing 10% (v/v) foetal bovine serum (FBS) and 2 mM L-Glutamine. The cells were then seeded at 20000 cells/well into 384-well plates containing compounds at concentrations ranging from 3.125 µM to 15.9 pM. Following 24 hours incubation (37 °C, 5% CO_2_), media was removed and ONE-Glow^TM^ EX luciferase reagent (Promega) was added to each well. Firefly luminescence was measured after 5 minutes using an Envision (PerkinElmer). NanoDLR^TM^ Stop and Glo® reagent (Promega) was added and incubated for ten minutes (room temperature), before measuring Nano luminescence using the Envision. Wells containing vehicle or 3 µM Fulvestrant were used to normalise data, and degradation of ESR1-Nanoluciferase was analysed using Genedata Screener.

### Statistics and reproducibility

Data were analyzed utilizing GraphPad Prism version 9 (GraphPad Prism Software, Inc., CA, USA) or GeneData Screener (Genedata, Switzerland) and expressed as means ± SD. The accompanying figure legends provide comprehensive information on the data processing methodology.

### Reporting summary

Further information on research design is available in the Nature Portfolio Reporting Summary linked to this article.

### Supplementary information

Peer Review File

Supplementary Information

Description of Additional Supplementary Files

Supplementary Data

Reporting Summary

## Data Availability

The authors declare that the data supporting the findings of this study are available within the paper and its Supplementary Information files. Should any raw data files be needed in another format they are available from the corresponding author upon reasonable request. Uncropped Western blots are available in the Supplementary Information, Figure S9. The source data behind the graphs and tables in the paper can be found in the Supplementary Data file.
