# Supplementary material for: Metabolism-driven in vitro/in vivo disconnect of an oral ERɑ VHL-PROTAC
Source: Commun Biol. 2024 May 13;7:563. doi: 10.1038/s42003-024-06238-x (PMC11091220; doi:10.1038/s42003-024-06238-x)
Supplement: Supplementary file 2 — Supplementary Information [file 42003_2024_6238_MOESM2_ESM.pdf]

Supplementary Information

Contents

1. **Supplementary Note 1: 3D Conformer of AZ’6421 (5) free in solution** 2
2. **Supplementary Note 2: Ternary complex modelling of ERα, VHL and AZ’6421 (5)** 6
3. **Supplementary Note 3: Chemistry** 10
   a. **Conformational Analysis by NMR** 10
   b. **Preparation of compounds** 17
      i. .... **General information:** 17
      ii. .... **Abbreviations** 18
      iii. .... **Synthetic procedures** 19
      iv. .... **LCMS chromatograms of final compounds** 64
4. **Table S4: Binding, degradation and mouse hepatocyte data for SAR exploration**. 73
5. **Figure S9: Uncropped Western blotting images** 74
6. **Supplementary References** 77

1. Supplementary Note 1: 3D Conformer of AZ'6421 (5) free in solution

PROTACs are prone to intramolecular interactions that can substantially modify their drug-like properties.[1] Here we used solution NMR spectroscopy to determine the 3D conformer of **5** free in solution. Initially, water/DMSO mixtures were utilised to enhance solubility of these highly lipophilic compounds in a relevant protic solvent. However, The 1D ^{1}H-NMR signals of **5** in a mixture of DMSO and D_2O of (2:1) were observed to be very similar to those in pure DMSO (Figure S3) and therefore pure DMSO was used for simplicity. By two 1D ^{1}H-NMR based methods A-NMR[2] and temperature-dependent chemical shift perturbation in DMSO[3] we found that the N-terminal amide HBD of (*S,R,S*)-AHPC shows an A-NMR value of 0.03 and temperature-dependent perturbation of -2.6 ppb/K. These values are both indicative of HBD shielding, whereas the benzyl-amide NH donor of the VHL binding motif and the indole NH in the ERα binding substructure show values characteristic of solvent exposure. By 2D rotating-frame nuclear Overhauser effect (NOE) and ^{1}H-^{13}C long-range correlation (^{13}C-HSQMBC) NMR spectroscopy we determined distance and tortional angle restraints for 3D structure calculation showing an intra-molecular H-bond to the ether oxygen proximal to the N-terminus of (*S,R,S*)-AHPC. Deletion of this ether oxygen from the N-terminus of (*S,R,S*)-AHPC produces a large chemical change of 0.6 ppm for the NH donor, whereas most other ^{1}H signals remain unchanged (Figure S3). NMR signals for the ERα and VHL protein-binding termini of the PROTAC did not show significant differences when compared to 1D NMR spectra of their small-molecule counterparts (Figure S3). Interestingly, key NOE signals that reflect the intramolecular hydrogen bond (IMHB) and the *tert*-butyl to benzyl contacts (Figure S1) are preserved in $CDCl_3$ and a mixture of DMSO and D_2O of (2:1), which suggests that a collapsed conformational ensemble is present in polar and apolar solvent environments. (Figure S2b).

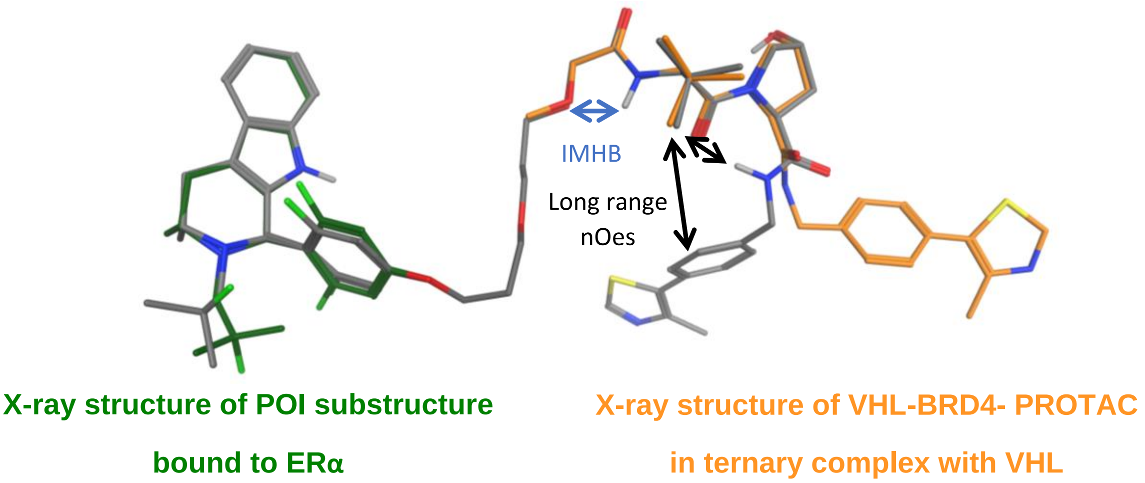

Figure S1. An example NMR conformation satisfying the NOE constraints (grey) overlaid with partial X-ray crystal structures of AZD9496 in ERα (green) and MZ1 in VHL (orange)

Free-in-solution NMR data of AZ'6421 (5)

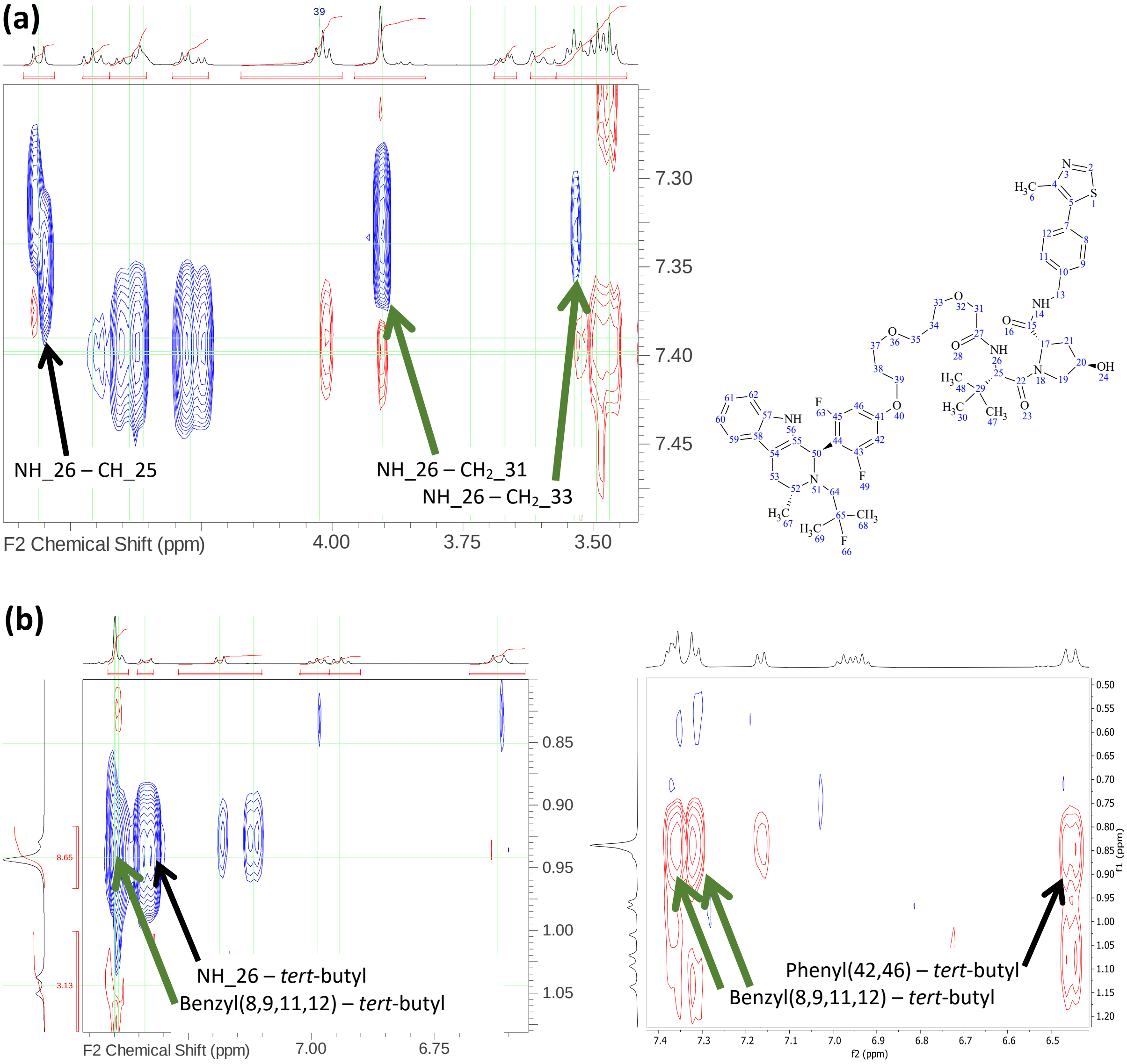

Figure S2. (**a**) 2D ROESY of **5** in 100% DMSO-d6 @ 37C showing the NOEs between the shielded NH_26 and methylenes 31 and 33, which determine the IMHB acceptor atom O_32. (**b**) 2D ROESY of **5** in 100% DMSO-d6 @ 37C (left) and 33% D_2O/67% DMSO-d_6 @ 27C (right) showing the NOEs between the *tert*-Butyl and benzyl hydrogens that determine the hydrophobic folding of **5**.

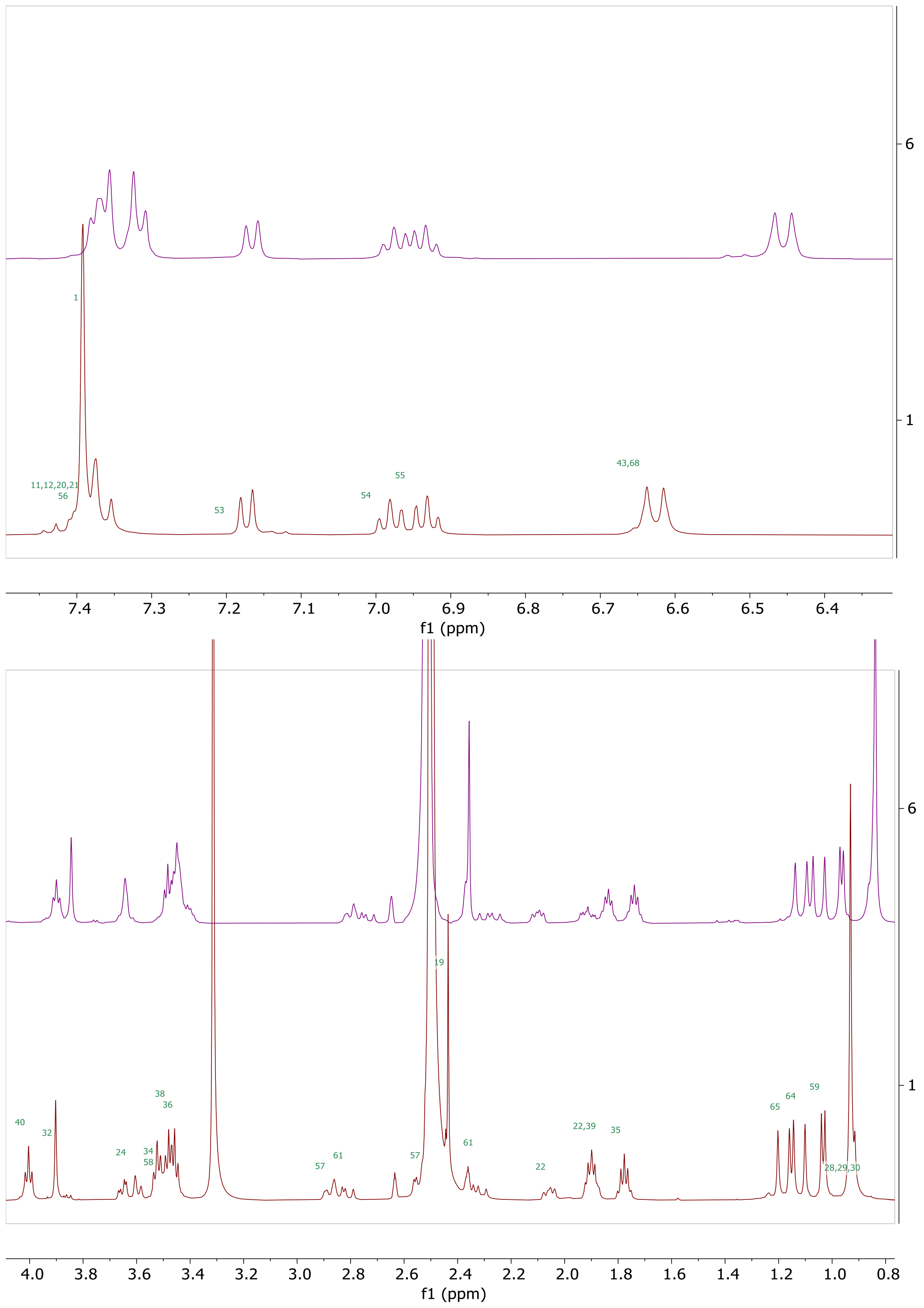

Figure S3. 1D ^{1}H-NMR of **5** in 33% D_2O/67% DMSO-d_6 (upper spectrum) and 100% DMSO-d_6 (lower spectrum) @ 27 ^{o}C showing similar ^{1}H chemical shifts for key aromatic (left) and aliphatic (right) hydrogens.

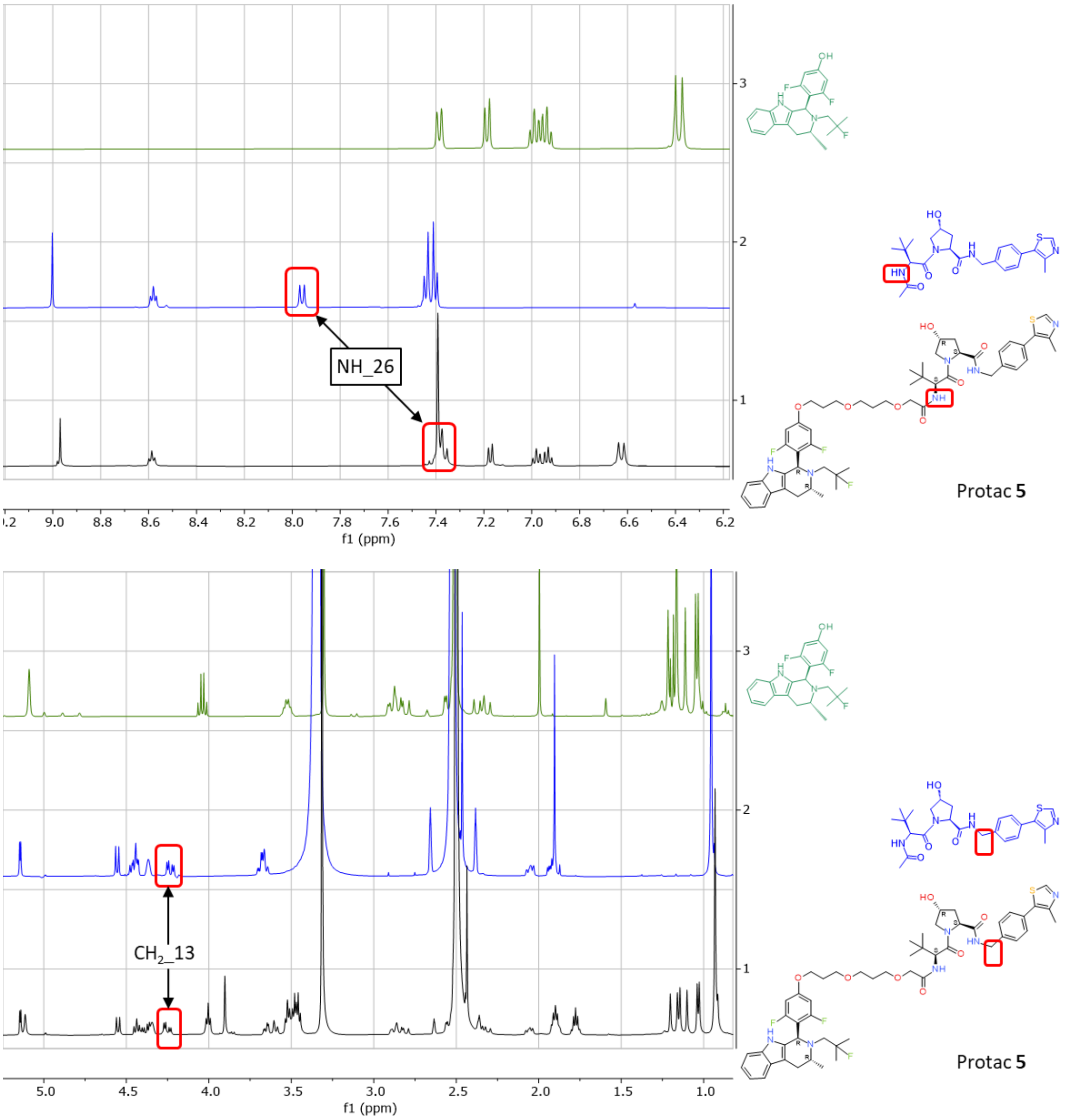

Figure S4. 1D ^{1}H-NMR spectra of **5** and its POI (green) and acetylated-(*S,R,S*)-AHPC (blue) substructures in 100% DMSO-d_6 @ 27 ^{o}C showing similar ^{1}H chemical shifts for most hydrogens, but a large 0.6 ppm upfield chemical shift for NH_26 in the absence of its IMHB acceptor. The benzylic CH_2_13 preserves a magnetically non-equivalent dd coupling pattern in the isolated VHL substructure, suggesting pre-organisation of the amide-benzyl bond similar to **5**.

2. Supplementary Note 2: Ternary complex modelling of ERα, VHL and AZ’6421 (5)

Ternary complex modelling was initiated to assess whether the solution conformer ensemble suggested by NMR would be compatible with the binding mode of **5** when bound in a ternary complex. No ternary complexes of VHL and ER have been reported to date, nor is it a given that there will only be a single ternary complex leading to degradation. Predicting ternary complexes is not routinely done, though approaches have been suggested.[4]

Here, we apply a heuristic approach to establish whether ternary complexes can be formed, and what they might look like. In order to generate a reasonable start orientation for a ternary complex of estrogen receptor and VHL, mediated by **5** (AZ’6421), we started from existing complexes of Brd4 with VHL, i.e. 5T35, 6SIS, and 7KHH.[5-7] Other known complexes of VHL and PROTACs with SMARCA structures were checked as well, but proved less usable as start points (PDB codes 6HAX, 6HAY, 6HR2).

It is notable that the Brd4-PROTAC-VHL orientations from three Brd4 ternary structures are very different in two cases. Structures 6SIS and 5T35 are similar, which is explained by the macrocyclic 6SIS ligand having been modelled to mimic the binding pose seen in 5T35. The difference between 7KHH and 5T35 is apparent from a simple comparison. They contain the largely similar Brd4-BD1 and -BD2 bromodomains. Figure S5 shows orientations of the bromodomains relative to VHL.

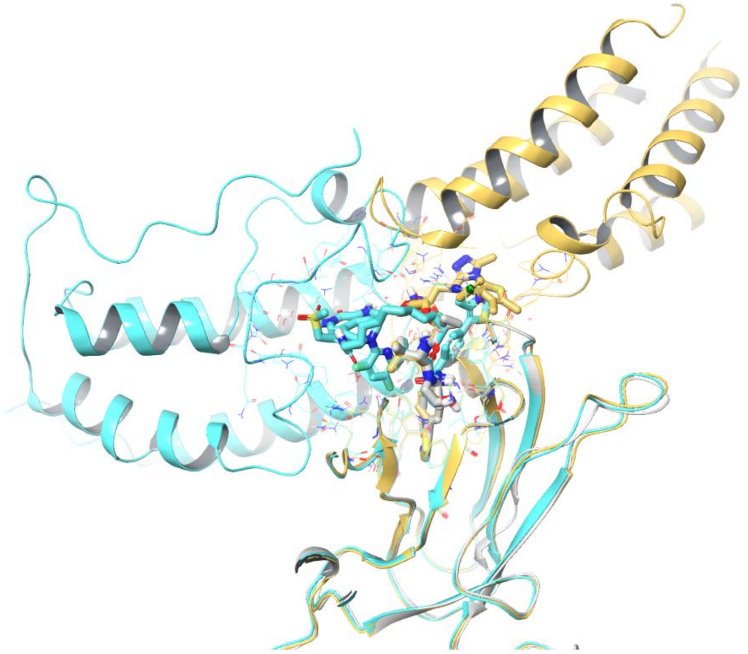

Figure S5. Comparison of PDB structures 5T35 (orange) and 7KHH (cyan), aligned on the VHL portion of their structures (visible centre bottom). A binary VHL structure containing only a VHL ligand shows excellent alignment to the VHL portion of both ternary structures (shown in white ribbons, centre bottom, PDB code 5NVX)

Inspection of the ligands of 5T35 and 7KHH suggested that their linker length was similar to that of AZ'6421. The ligands from the ternary structures were truncated to comprise VHL binder and linker and were subsequently connected to the ERα binder portion to construct VHL-bound versions of AZ'6421. We used the binding pose of AZD9496 in the ERα ligand-binding domain as observed in PDB structure 5ACC,[8] as the 1D-NMR trace of AZD946 had been observed to be very similar to the signals seen in the PROTAC NMR, from which we concluded they occupy the same conformation in both cases.

We connected the ERα binder to the linker with a reasonable starting conformation. As there is a twofold symmetry in the top ring of the ER-binding portion of the ligand, the ERα core was connected to the linker+VHL portion pointing in either direction.

Following this procedure, the ERα and VHL proteins were assembled to bind with the terminal parts of the PROTAC as dictated by the original structures (5T35 and 7KHH for VHL, and 5ACC for ERα), and a local minimisation was applied. It was clear from the initial modelling that the VHL/ERα orientation that was built using the orientation apparent in 7KHH was not productive. In these preliminary ternary models, protein-protein clashes were apparent that appeared unresolvable without major changes in orientation of their constituent proteins orientations, which, in turn, would require large changes in conformation of the PROTAC.

One ternary model based on 5T35 appeared acceptable, and was taken forward. The ERα portion of the complex was replaced by a WT homology model of the ERα ligand-binding domain in the same conformation to rectify the crystallographic residue mutations. This model was minimised and subjected to a molecular dynamics simulation to establish whether the ternary association was at least stable on the timescale of 10s to 100s of nanoseconds (Figure S6). The change in shape of the protein over the time of the simulation can be tracked using the RMSd relative to the start orientation (Figure S6a) and is seen to be variable initially but reasonably stable after approx. 100 ns. Figure S6b shows the stability of the secondary structure of the constituent proteins, and this indicates that secondary structure is largely stable, but that there are variable regions, explaining the variability of the RMSd. Other simulations did not prove as stable and showed disassembly of the binding mode on the VHL side.

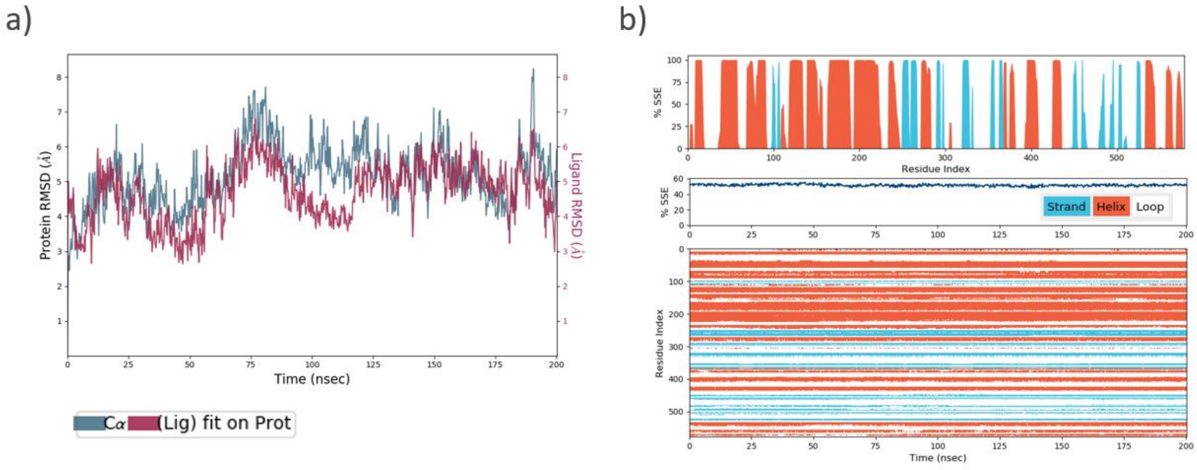

Figure S6. a) RMSd of protein backbone and ligand relative to original protein or ligand shape over the course of the simulation. b) Stability of secondary structure (SSE, secondary structure elements) of both proteins over the time of the trajectory.

Closer inspection of the binding modes suggests that, while the proteins remain roughly in the same orientation, the PROTAC in between is quite variable in placement.

Figures S7a and S7b show an example ternary binding mode and a detailed view of the ligand conformation. Significant shifts can be observed in the orientation of the ERα protein relative to VHL. The ligand binding mode changes accordingly. While the binding mode of the PROTAC itself changes significantly over time, it is clear that a folded-up conformation of the PROTAC is maintained, and its fold suggests distances similar to those observed by solution NMR (Figure S8). This, in turn, confirms that a solution conformational ensemble may be at least in part compatible with the binding modes observed here.

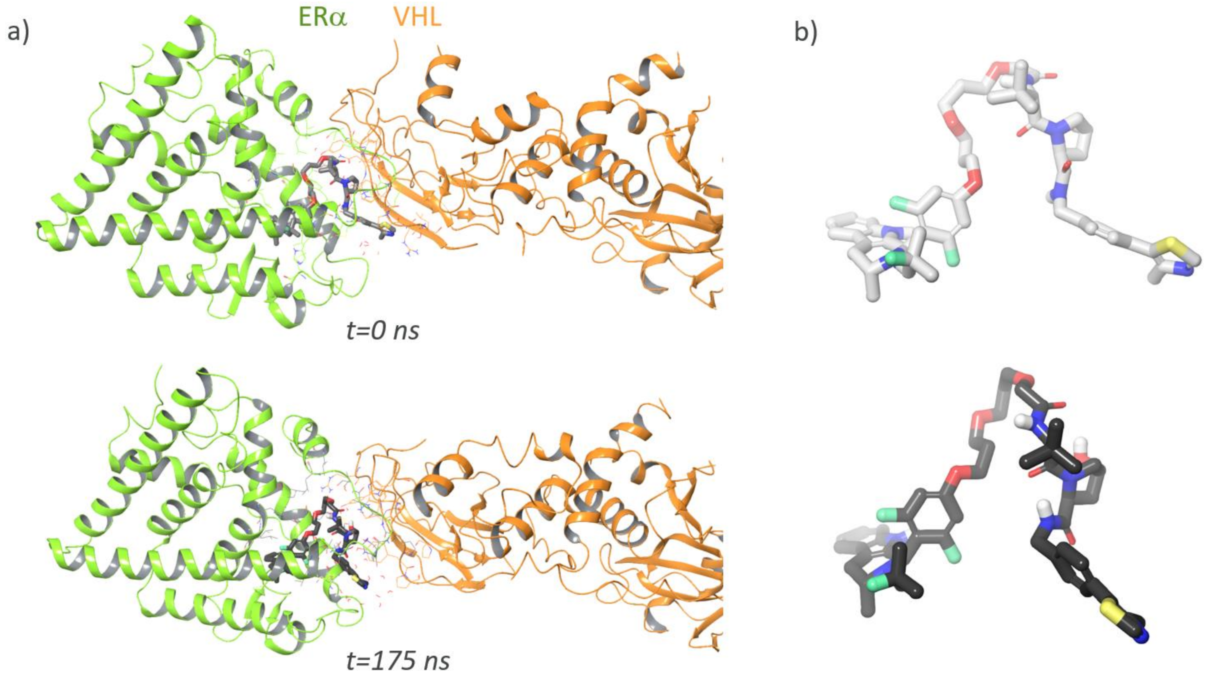

Figure S7 Example of a typical ternary complex binding mode and orientation at t=0 ns (top), t=150 ns (bottom) a) full complex. b) PROTAC conformation.

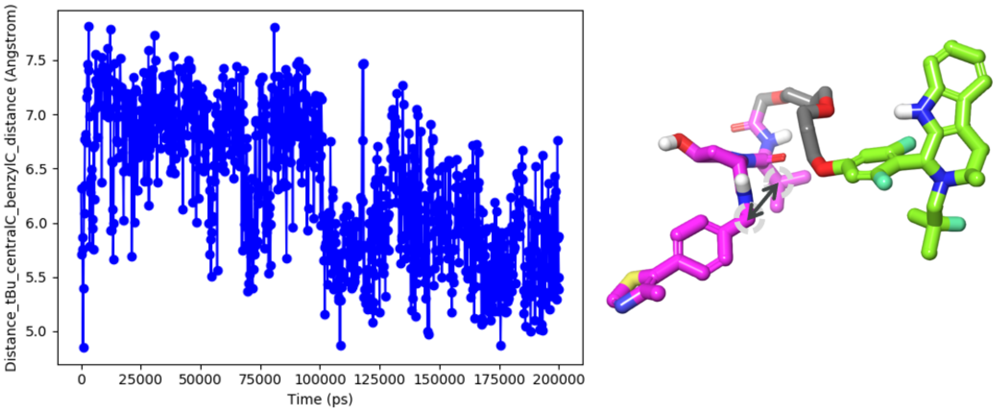

Figure S8. Distance of the central carbon of the tert-butyl of the VHL ligand to the benzyl carbon (arrow), over the course of the trajectory.

3. Supplementary Note 3: Chemistry

a. Conformational Analysis by NMR

All NMR spectra were recorded on a Bruker 500 MHz instrument equipped with a 5 mm QNP cryoprobe. Chemical shifts (δ values) are given in parts per million (ppm) and referenced to the DMSO (2.50 ppm) and $CDCl_3$ (7.26 ppm) residual signals. For the structural assignment of the molecule the following spectra were acquired both in DMSO_d6, DMSO_d6:D2O (2:1) and $CDCl_3$: 1D ^{1}H, 2D COSY, 2D ^{13}C-HSQC and 2D ^{13}C-HMBC at 300 K and 310 K using the standard pulse sequences available in TopSpin 4.0 (Bruker Biospin GmbH). 3D conformational distance restraints were measured by 2D ROESY (mixing time 300 ms; relaxation delay 5 s) and dihedral angle restraints were obtained from 2D ^{13}C-HSQMBC NMR spectra in 100% DMSO_d6 at 310.[9] To acquire NMR data in aqueous media, a titration was carried out on a DMSO sample, adding incremental amounts (10, 15, 25 and 33%) of D_2O till initial precipitation of **5** was observed.

To determine the relative NOE intensities for pairs of spins, F2-slices were extracted from the 2D ROESY and used for the accurate integration of cross-peak and diagonal signals[1]. The PANIC method (Peak Amplitude Normalisation for improved cross-relaxation) was applied,[10,11] where the NOE intensities were normalised relative to the diagonal for each slice. Correction factors were then applied to compensate for the number of spins in each environment (corrected integral). For each molecule the integral for two protons at a known distance was used as a reference to calibrate the other interproton distances in the molecule using the equation below:[12]

$$\frac{\eta_{I1S}}{\eta_{I2S}} = \frac{{r_{I1s}}^{-6}}{{r_{I2s}}^{-6}}$$

where η_{IS} is the intensity of the NOE between spins I and S (S being the inverted spin) and ${r_{Is}}^{-6}$ is the internuclear distance between I and S. The NOEs were therefore converted into distance restraints. For those NOEs that could not be integrated due to signal overlap a detection limit default restraint distance of 5.5 Å was estimated.

The flexible linker of **5** and the adjacent VHL ligand of **5** were further constrained by dihedral angle measurements from 2D ^{13}C-HSQMBC NMR spectra, using the in-phase / anti-phase method for accurate extraction of coupling constants.[9] This method yields experimental three-bond ^{1}H-^{13}C coupling constants which are subsequentially matched against three-bond ^{1}H-^{13}C coupling constants calculated by quantum mechanics for each 3D conformer.

Molecular Dynamics (MD) and Quantum Mechanics (QM) Calculations of **5**

The available 3D conformational space of **5** was computed by molecular dynamics (MD) in an explicit water box with simulated 0.15 M NaCl using the “desmond replica exchange” tool in Maestro (Schrödinger 2020, https://www.schrodinger.com/products/desmond). For this pH=7.0 ± 0.0 protonation states were used, and the OPLS3e force field was customized for **5** with the “force field builder” tool in Maestro. Desmond replica exchange with solute tempering molecular dynamics was run with 16 replicas at temperatures between 300 K and approximately 1300 K for 100 ns, and frames were saved every 0.1 ns, yielding a total of 1000 conformer snapshots.

The numerous rotatable bonds of **5** required to two separate MD runs for thorough conformational sampling of the 3D space. The first run included the POI, the linker and the VHL-ligand N-terminus substructure of **5** all the way to atom group 25, which covered the linker flexibility. The second run included the entire structure of **5**, which covered the conformational space of the VHL ligand, but only partially the space of the linker. For each MD run with 1000 distinct 3D conformers the 20 most diverse representatives, as measured by dissimilarity in coordinate rmsd, were refined by quantum mechanics (QM) calculations using the software MOE (Chemical Computing Group: Molecular Operating Environment (MOE), version 2019.01) and the settings opt=(tight,RecalcFC=5,MaxCycles=5000) and Int=SuperFineGrid, as recommended by the manufacturer. For each refined 3D conformer ^{1}H and ^{13}C chemical shifts and ^{1}H-^{1}H as well as ^{1}H-^{13}C coupling constants were calculated using the software Gaussian version 16 (Revision C.01) with the GIAO DFT method at the B3LYP/6-31G* level with PCM solvent modeling using a dielectric constant of 78.4, which mimicks water. Finally the experimental distance and dihedral angle restraints in Tables S2 and S3 were matched against the calculated values for each conformer using a customized Namfis least-square fitting tool in the software MOE, as described previously.[13] This yielded the best fitting 3D conformer of **5** displayed in Figure S1.

NMR chemical shift assignment and conformational analysis of 5

Table S1. NMR chemical shift assignments of **5** in DMSO_d6 at 310 K

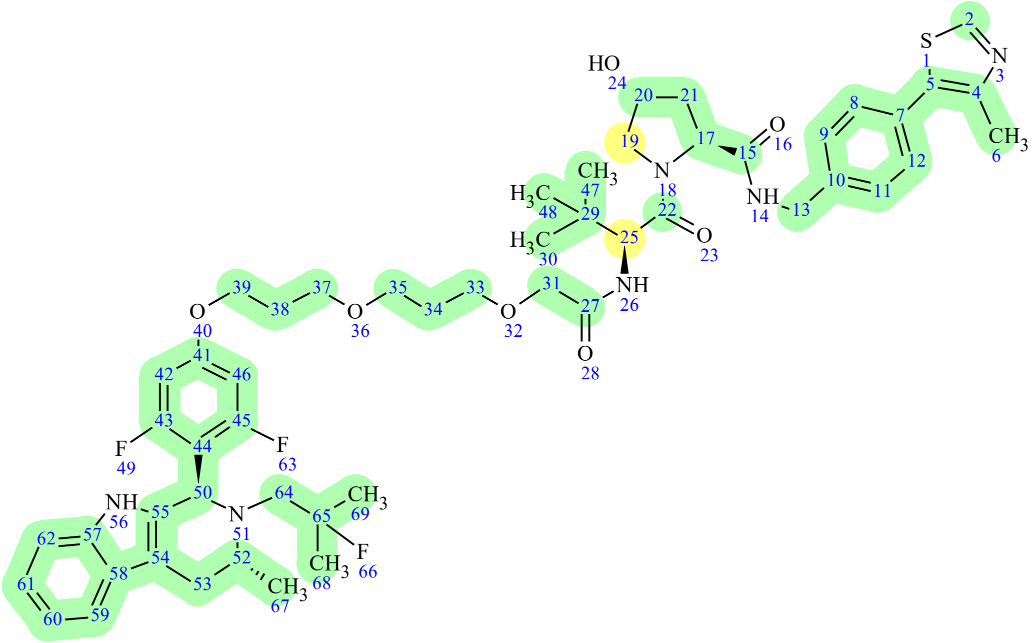

Atom#	C Shift	C Calc Shift (HOSE)	H Shift	H Calc Shift (HOSE)	H Multiplicity	No. of protons
2	151.8	150.3	8.961	8.978	m	1
4	148.2	148.4				
5	131.5	131.6				
6	16.3	16.0	2.445	2.432	m	3
7	130.2	130.9				
8, 12	128.0	127.7	7.400	7.644	m	2
9, 11	129.1	127.7	7.398	7.361	m	2
10	139.8	137.9				
13	42.2	41.2	4.386	4.368	m	1
13	42.2	41.2	4.270	4.248	m	1
14			8.532	8.579	t (5.95, 5.95)	1
15	172.1	171.1				
17	59.2	58.5	4.457	4.507	m	1
19	57.0	57.2	3.669	3.986	m	1
19	57.0	57.2	3.610	3.596	m	1
20	69.3	68.7	4.360	4.335	m	1
21	38.3	37.6	1.922	1.984	m	1
21	38.3	37.6	2.069	1.995	br dd (12.66, 8.09)	1
22	169.6	170.5				
24			5.119	5.151	d (3.51)	1
25	56.1	60.4	4.561	4.615	d (9.61)	1
26			7.337	7.940	m	1
27	168.9	170.6				
29	36.2	34.7				
30, 47, 48	26.6	26.2	0.942	0.916	m	9
31	69.9	68.9	3.902	3.926, 3.984	m	2
33	68.5	67.8	3.537	3.508	m	2
34	29.9	29.0	1.788	1.593	quin (6.37, 6.37, 6.37, 6.37)	2
35	67.4	67.8	3.469	3.395, 3.501	m	2

Atom#	C Shift	C Calc Shift (HOSE)	H Shift	H Calc Shift (HOSE)	H Multiplicity	No. of protons
37	66.9	67.5	3.494	3.395, 3.501	m	2
38	29.2	29.0	1.914	2.000	m	2
39	66.0	63.8	4.023	4.124	m	2
41	160.1	160.8				
42, 46	99.1	99.0	6.623	6.602	m	2
43, 45	161.7	162.0				
44	109.6	108.8				
50			5.129	5.188	s	1
52	51.0	51.1	3.522	3.501	m	1
53	27.1	26.8	2.893	2.826	m	2
53	27.1	26.8	2.544	2.731	m	1
54	106.8	106.2				
55	133.1	132.7				
56			10.465	10.534	s	1
57	136.7	136.3				
58	127.4	126.9				
59	117.9	117.6	7.390	7.532	m	1
60	118.5	118.0	6.942	6.979	m	1
61	120.7	120.4	6.988	7.054	m	1
62	111.3	111.0	7.186	7.310	d (7.93)	1
64	56.6	56.7	2.355	2.347, 2.382	m	1
65	98.0	98.4				
67	13.2	13.2	1.044	1.039	d (6.56)	3
68, 69	25.1	24.9	1.163	1.152	d (5.34)	3

Table S2. Experimental NOE and Inter-proton distances of **5** derived from 2D ROESY NMR spectra in DMSO_d6 at 310 K.

H atom# 1	H atom# 2	Calculated distance (Å)
47,48,30	9,11	5.4
47,48,30	26	4.2
47,48,30	14	7.3
47,48,30	31	6.3
47,48,30	19'	4.4
26	31	2.9
26	34	4.2
26	33	4.0
14	17	2.0
14	21''	3.6
14	13'	2.4
14	13''	2.4
25	19''	2.5
25	19'	2.1
25	47,48,30	3.0
26	31	2.9
26	34	4.2
26	33	4.0

Table S3. Experimental Long-range ^1H-^{13}C coupling constants of **5** derived from 2D ^{13}C-HSQMBC NMR spectra in DMSO_d6 at 310 K.

H atom#	C atom#	Coupling constant (Hz)
31	33	4.2
31	27	4.3
39	41	2.5
39	38	2.8
39	37	4.4
25	29	4.1
25	22	4.2
25	27	3.3
13'	9,11	3.8
13''	9,11	2.5
35	37	2.8
35	33	4.5
37	39	4.8
37	35	3.0
64'	50	5.2
64''	50	4.5

NMR spectra (100% DMSO_d6)

^1H NMR

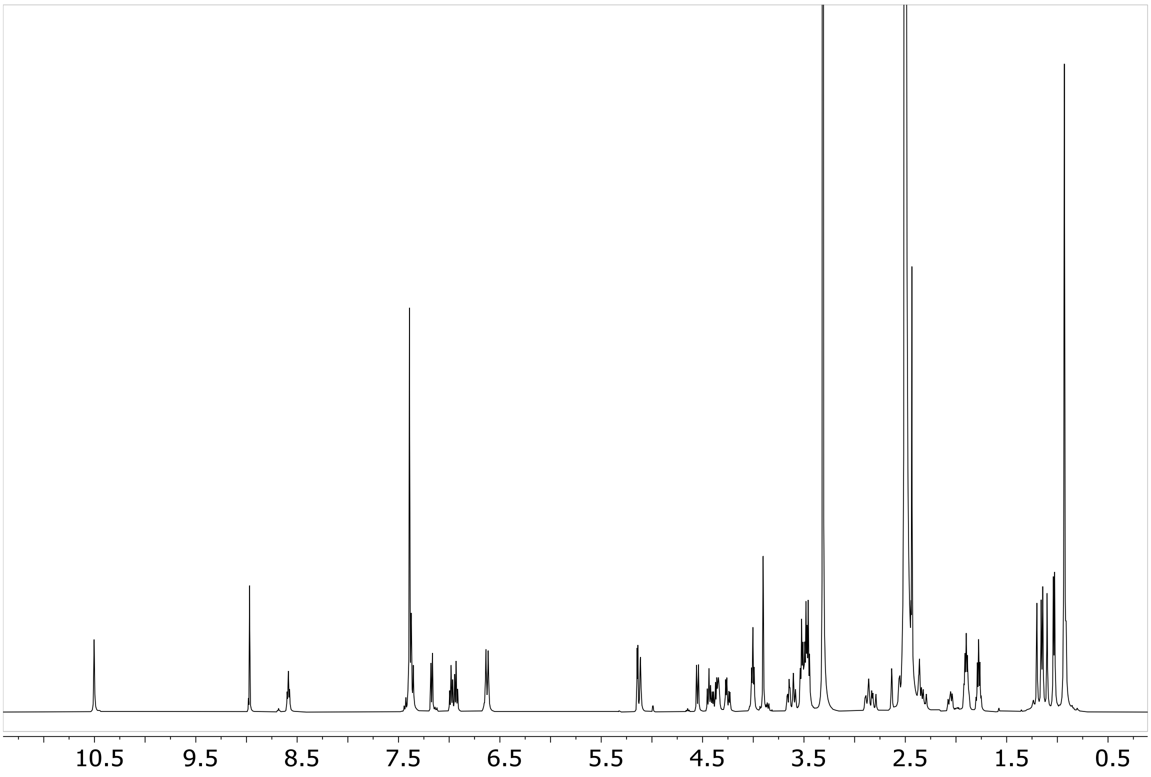

^{1}H COSY

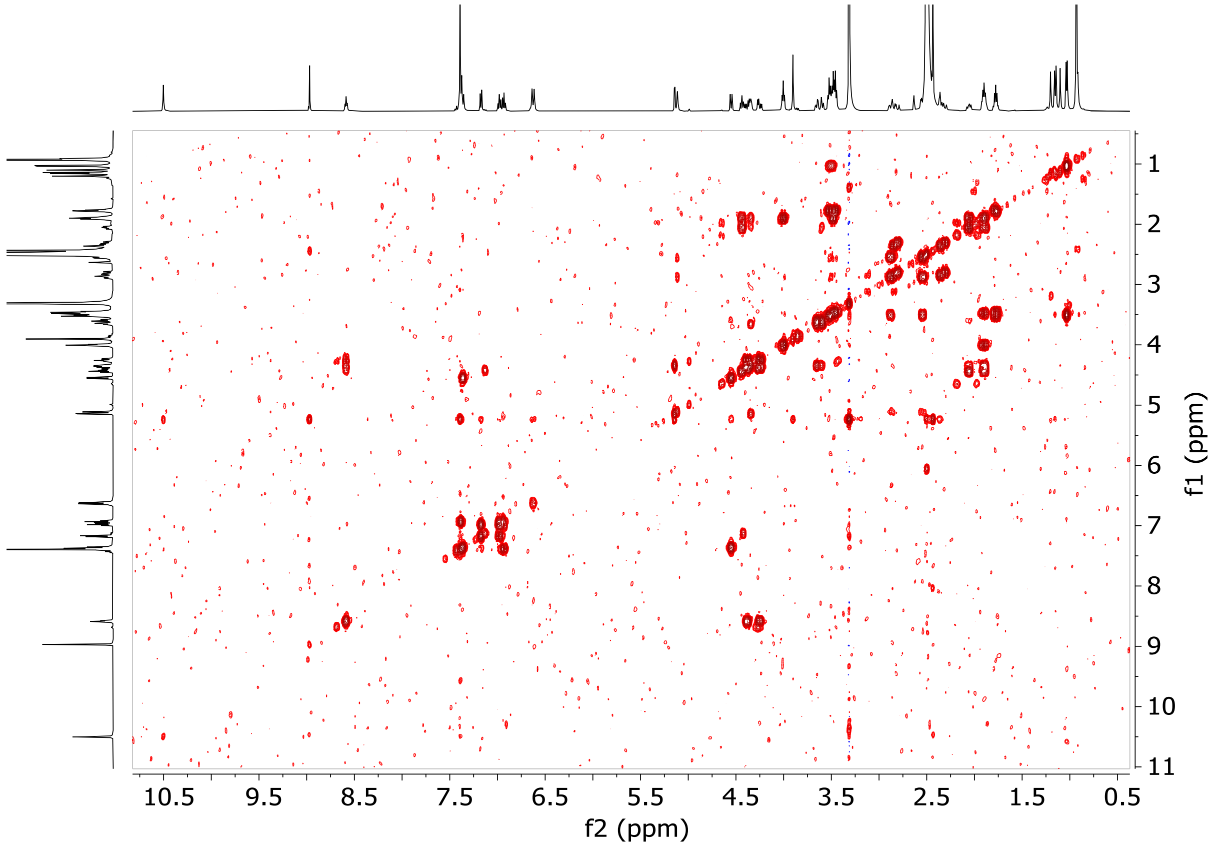

^{13}C HSQC (multiplicity edited)

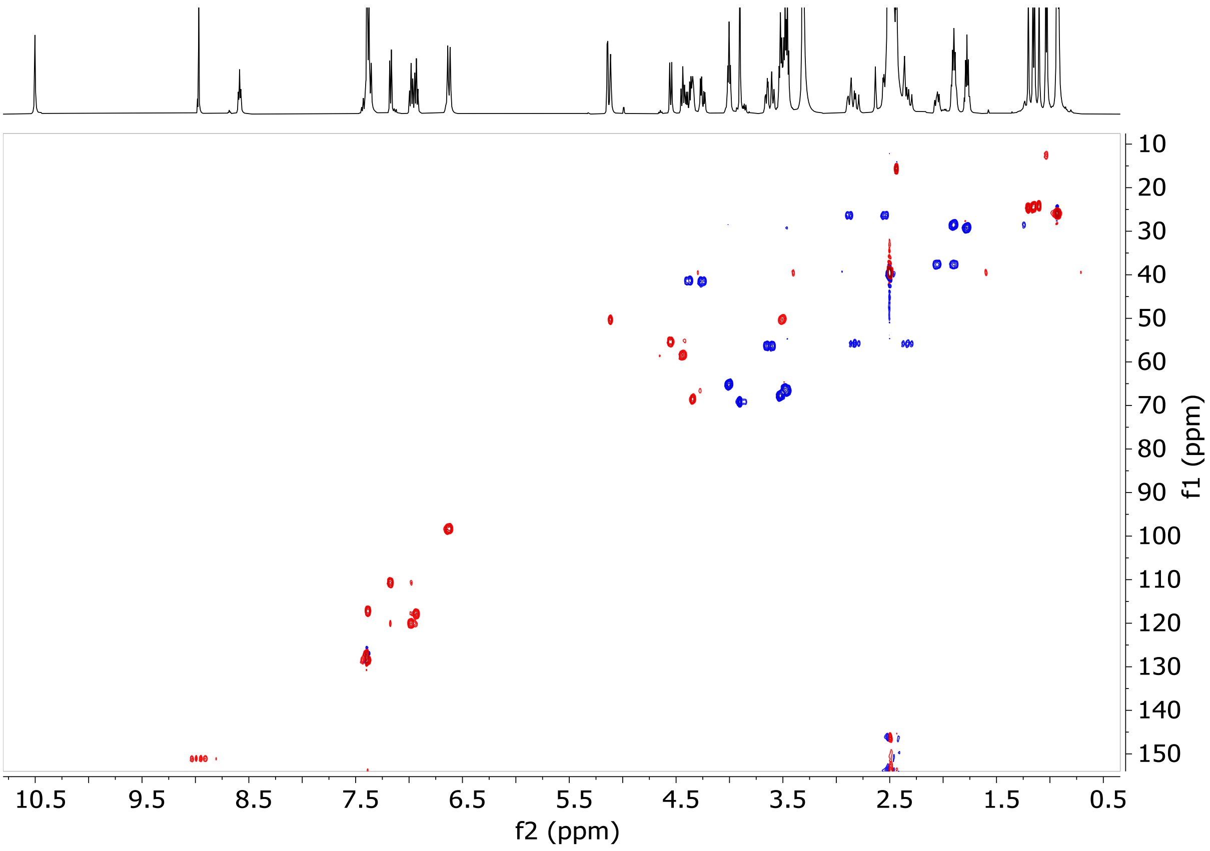

^{1}H, ^{1}H ROESY

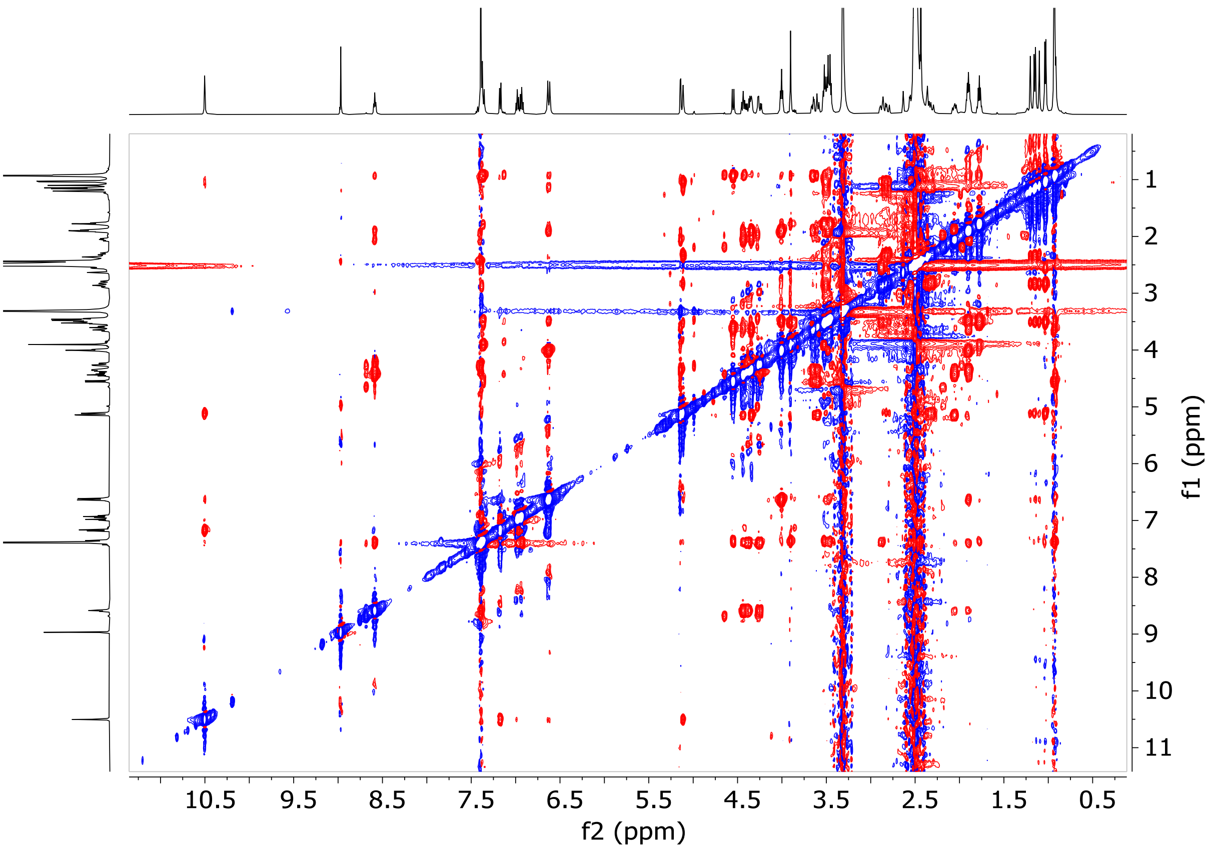

b. Preparation of compounds

i. General information:

All starting materials, reagents and solvents were sourced from commercial suppliers and used as received unless otherwise stated.

All reactions were carried out in round bottom flasks equipped with a magnetic stir bar under a nitrogen atmosphere unless otherwise noted. Reactions were monitored using Ultra Pressure Liquid Chromatography Mass Spectrometry (LCMS). Ultra Pressure Liquid Chromatography mass spectra (m/z: ES+/-) were obtained using a Waters UPLC fitted with a Waters SQD, SQD2 or QDA mass spectrometer under electrospray ionisation with positive/negative switching. The samples were run on a Waters Acquity CSH^{TM} C18 1.7 µm 2.1 x 50 mm column using 0.1% NH_3 or 0.1% formic acid in water and acetonitrile under a gradient from 3% to 97% of acetonitrile over 1.5 min at a flow rate of 1 mL/ min. UV detection was collected by diode array scanning 220nm-320nm. Normal phase chromatography was performed on Teledyne Isco CombiFlash® Rf instruments using prepacked Puriflash high capacity Silica columns (50 µm, spherical particles) or Redisep Rf Gold® Amine silica columns (20-40 50 µm, spherical particles). HPLC purification was performed on a Gilson preparative HPLC-MS system with a PerkinElmer SQ300 MS detector, using Waters CSH C18 OBD column, 5µ silica, 30 mm diameter, 100 mm length and a gradient of 5%-95% acetonitrile in water with either 0.1% formic acid or 0.1% NH_3 as pH modifier and a flow rate of 50 mL/min.

NMR spectra (^{1}H and ^{13}C) were obtained on Bruker Avance 400 MHz spectrometers at 300K using d_6-DMSO as solvent and as internal reference (residual shift at 2.50 ppm ^{1}H, 39.52 ppm ^{13}C) unless otherwise noted. ^{1}H NMR data are reported in the form: δ (multiplicity, coupling constants, number of protons). The following abbreviations have been used: s, singlet; d, doublet; t, triplet; dd, doublet of doublet; dt = doublet of triplets; td = triplet of doublets; tt = triplet of triplets; ddd = doublet of doublet of doublets; m = multiplet.

High Resolution Mass Spectra (HRMS) were obtained using a Waters Orbitrap XL or Waters Xevo Qtof spectrometer in Electrospray (ESI) +ve or -ve ion mode and automatic MSMS using CID at 35eV is carried out automatically on the two biggest ions generated from MS1. Purity criteria: Compounds are > 95% purity based on UPLC and ^{1}H NMR.

ii. Abbreviations

AcOH	acetic acid	aq.	Aqueous
DCM	dichloromethane	DIPEA	*N,N*-diisopropylethylamine
DIAD	diisopropyl azodicarboxylate		
DMF	*N*,*N*-dimethylformamide	DMSO	Dimethyl sulfoxide
eq.	equivalents	ESI-HRMS	electrospray ionisation – high resolution mass spectrometry
Et_2O	diethyl ether	EtOAc	ethyl acetate
EtOH	ethanol	HATU	2-(3*H*-[1,2,3]Triazolo[4,5-b]pyridin-3-yl)-1,1,3,3-tetramethylisouronium hexafluorophosphate(V)
MeCN	acetonitrile	MeOD	d_4-methanol
MeOH	methanol	*m/z*	mass spectrometry peak(s)
RockPhos Pd G3	[(2-Di-*tert*-butylphosphino-3-methoxy-6-methyl-2′,4′,6′-triisopropyl-1,1′-biphenyl)-2-(2-aminobiphenyl)]palladium(II) methanesulfonate	RT	room temperature
TBAF	tetra *n*-butylammonium fluoride	THF	tetrahydrofuran
Sat.	saturated	$scCO_2$	Supercritical carbon dioxide
SCX	Strong cation exchange	SFC	Supercritical fluid chromatography

iii. **Synthetic procedures**

<u>(1*R*,3*R*)-1-(4-Bromo-2,6-difluorophenyl)-2-(2-fluoro-2-methylpropyl)-3-methyl-2,3,4,9-tetrahydro-1*H*-pyrido[3,4-*b*]indole (1a)</u>

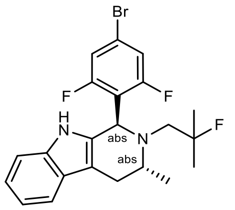

(*R*)-N-(1-(1H-Indol-3-yl)propan-2-yl)-2-fluoro-2-methylpropan-1-amine (5.00 g, 20.1 mmol) was added to 4-bromo-2,6-difluorobenzaldehyde (4.8 g, 21.72 mmol) in toluene (95 ml) and acetic acid (7 mL) at RT under nitrogen. The resulting solution was stirred at 80 °C for 20 hours. The reaction mixture was evaporated to dryness, redissolved in heptane (50 mL), evaporated to dryness, redissolved in Et_2O (50 mL), evaporated to dryness and redissolved in Et_2O (90 mL). The resulting suspension was filtered. 2M HCl in Et_2O (12 mL) was added dropwise to the filtrate and the resulting suspension stirred for 20 minutes. The reaction mixture was filtered and the precipitate washed with Et_2O (2x 50 mL). The crude solid was partitioned between EtOAc (200 mL) and 2 M aq. potassium carbonate (50 mL). The aqueous layer was removed and the organic layer washed sequentially with 2 M aq. potassium carbonate (20 mL) and sat. brine (10 mL). The organic layer was dried over magnesium sulfate, filtered and evaporated to crude product. The residue was purified by flash silica chromatography, elution gradient 0 to 15% EtOAc in heptane. Pure fractions were evaporated to dryness to afford the title compound (6.87 g, 76%) as a pale yellow solid; ^{1}H NMR (400 MHz, DMSO-d6, 30°C) 1.05 (d, *J* = 6.5 Hz, 3H), 1.18 (dd, *J* = 34.1, 21.4 Hz, 6H), 2.34 (dd, *J* = 23.1, 15.1 Hz, 1H), 2.58 (dd, *J* = 15.0, 4.3 Hz, 1H), 2.8 – 2.98 (m, 2H), 3.51 (q, *J* = 5.0 Hz, 1H), 5.19 (s, 1H), 6.91 – 7.06 (m, 2H), 7.19 (d, *J* = 7.8 Hz, 1H), 7.41 (dd, *J* = 7.8, 4.0 Hz, 3H), 10.55 (s, 1H); *m/z*: ES+ $[M+H]^+$ 451.0.

<u>2-(3,5-Difluoro-4-((1*R*,3*R*)-2-(2-fluoro-2-methylpropyl)-3-methyl-2,3,4,9-tetrahydro-1H-pyrido[3,4-*b*]indol-1-yl)phenoxy)ethan-1-ol (1b)</u>

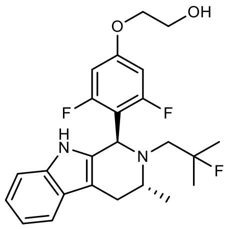

Ethane-1,2-diol (0.17 mL, 4.0 mmol) was added to (1*R*,3*R*)-1-(4-bromo-2,6-difluorophenyl)-2-(2-fluoro-2-methylpropyl)-3-methyl-2,3,4,9-tetrahydro-1*H*-pyrido[3,4-b]indole (300 mg, 0.66 mmol), cesium carbonate (758 mg, 2.33 mmol) and RockPhos Pd G3 (27.9 mg, 0.03 mmol) in toluene (5 mL) at RT under nitrogen. The resulting solution was vacuum degassed and stirred at 90 °C for 4.5 hours. The crude product was purified by flash silica chromatography, elution gradient 5 to 70% EtOAc in heptane. Pure fractions were evaporated to dryness to afford the title compound (87 mg, 30%) as a pale yellow dry film; ^{1}H NMR (400 MHz, $CDCl_3$, 30°C) 1.10 (3H, d), 1.21 (6H, dd), 1.89 (1H, t), 2.39 (1H, dd), 2.60 (1H, dd), 2.86 (1H, dd), 3.09 (1H, dd), 3.62 – 3.73 (1H, m), 3.91 – 4 (2H, m), 4.04 (2H, q), 5.20 (1H, s), 6.4 – 6.47 (2H, m), 7.05 – 7.15 (2H, m), 7.22 (1H, dd), 7.39 (1H, s), 7.51 (1H, dd); *m/z*: ES- $[M-H]^-$ 431.2.

2-(2-(3,5-Difluoro-4-((1*R*,3*R*)-2-(2-fluoro-2-methylpropyl)-3-methyl-2,3,4,9-tetrahydro-1H-pyrido[3,4-*b*]indol-1-yl)phenoxy)ethoxy)acetic acid (1c)

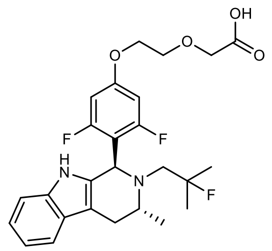

2-(3,5-Difluoro-4-((1*R*,3*R*)-2-(2-fluoro-2-methylpropyl)-3-methyl-2,3,4,9-tetrahydro-1H-pyrido[3,4-b]indol-1-yl)phenoxy)ethan-1-ol (80 mg, 0.18 mmol) was added to 60% sodium hydride in mineral oil (5.3 mg, 0.13 mmol) in THF (2.5 mL) and was stirred at RT for 5 minutes under nitrogen. *tert*-butyl bromoacetate (33 µL, 0.22 mmol) was added and the resulting suspension was stirred at RT for 2 hours. The reaction was incomplete and further 60% sodium hydride in mineral oil (3.5 mg, 0.09 mmol) was added and the suspension was stirred at RT for a further 2 hours. The reaction was incomplete and further *tert*-butyl bromoacetate (6.6 µL, 0.036 mmol) was added and the suspension was stirred at RT for a further 2 hours. The reaction was incomplete and further 60% sodium hydride in mineral oil (2.1 mg, 0.05 mmol) and THF (0.5 mL) was added and the suspension was stirred at RT for a further 2.5 hours. The reaction was incomplete and further 60% sodium hydride in mineral oil (2.1 mg, 0.05 mmol) and *tert*-butyl bromoacetate (5.5 µL, 0.04 mmol) were added and the suspension was stirred at RT for a further 12.5 hours. The reaction was incomplete and DMF (0.5 mL) was added and the temperature was increased to 50°C and the reaction mixture was stirred for a further 2 hours. The reaction mixture was slowly quenched with water (15 mL), extracted with EtOAc (3 x 15 mL), the

organic layer was dried over $MgSO_4$, filtered and evaporated to a dry film (130 mg) that was used in the next step without further purification; *m/z*: ES+ $[M+H]^+$ 491.4.

(2*S*,4*R*)-1-((*S*)-2-(2-(2-(3,5-Difluoro-4-((1*R*,3*R*)-2-(2-fluoro-2-methylpropyl)-3-methyl-2,3,4,9-tetrahydro-1*H*-pyrido[3,4-*b*]indol-1-yl)phenoxy)ethoxy)acetamido)-3,3-dimethylbutanoyl)-4-hydroxy-*N*-(4-(4-methylthiazol-5-yl)benzyl)pyrrolidine-2-carboxamide (1)

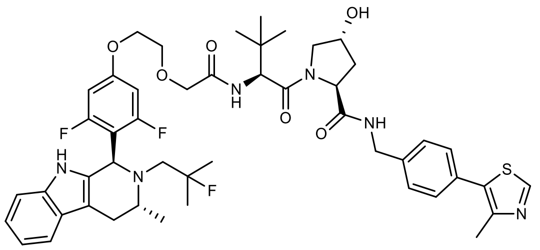

Crude 2-(2-(3,5-difluoro-4-((1*R*,3*R*)-2-(2-fluoro-2-methylpropyl)-3-methyl-2,3,4,9-tetrahydro-1*H*-pyrido[3,4-b]indol-1-yl)phenoxy)ethoxy)acetic acid (60 mg, 0.12 mmol), DIPEA (0.085 mL, 0.49 mmol), and HATU (70 mg, 0.18 mmol) were dissolved in DMF (4 mL) and stirred at RT over a period of 15 minutes under nitrogen. (2*S*,4*R*)-1-((S)-2-amino-3,3-dimethylbutanoyl)-4-hydroxy-*N*-(4-(4-methylthiazol-5-yl)benzyl)pyrrolidine-2-carboxamide, HCl[14] (57 mg, 0.12 mmol) was added to the reaction mixture at RT under nitrogen. The resulting solution was stirred at RT for 3.5 hours and diluted with EtOAc (10 mL), and washed sequentially with water (10 mL), sat. brine (10 mL), and water (10 mL). The organic layer was dried with $MgSO_4$, filtered and evaporated to afford crude product. The sample was purified using the following SFC conditions: Column: Princeton Diol, 30 x 250 mm, 5 micron Mobile phase: A = MeOH + 0.1% NH_3 / B = $scCO_2$ Gradient: 20-30% A over 10 minutes Flow rate: 100 mL/min BPR: 120 bar Temperature: 40 °C. Fractions containing the desired compound were evaporated to dryness to afford the title compound (36 mg, 32%) as a pale yellow solid; ^1H NMR (400 MHz, MeOD, 30°C) 1.02 (9H, s), 1.07 - 1.22 (9H, m), 2.10 (1H, m), 2.18 - 2.25 (1H, m), 2.34 - 2.41 (1H, m), 2.44 (3H, s), 2.58 (1H, dd), 2.83 - 2.92 (2H, m), 3.02 (1H, d), 3.67 (2H, s), 3.79 - 3.88 (2H, m), 3.88 - 3.92 (2H, m), 4.09 (2H, s), 4.15 - 4.2 (2H, m), 4.37 (1H, s), 4.44 (1H, s), 4.49 (2H, d), 4.55 - 4.6 (1H, m), 4.69 - 4.73 (1H, m), 5.19 (1H, s), 6.64 (2H, d), 6.92 - 7 (2H, m), 7.16 (1H, dd), 7.36 - 7.45 (5H, m), 7.63 (1H, d), 8.79 (1H, s); ^{13}C NMR (126 MHz, $CDCl_3$, 17°C) 12.6, 15.9, 24.7, 25.2, 26.4, 27.0, 35.1, 35.9, 43.3, 50.8,51.1, 56.6, 56.8, 57.1, 58.5, 67.7, 70.0, 70.2, 70.4, 97.4, 98.8, 108.3, 110.2, 110.8, 118.1, 119.1, 121.2, 127.6, 128.2, 129.5, 130.9, 131.7, 132.4, 136.3, 138.1, 148.3, 150.5, 159.4, 162.7, 170.1,170.6, 171.3; *m/z*: ES+ $[M+H]^+$ 903.6; LCMS purity = 100%; ESI-HRMS calculated for $C_{48}H_{58}F_3N_6O_6S$ $[M+H]^+$ = 903.4085, measured 903.4068.

2-(2-(3,5-Difluoro-4-((1*R*,3*R*)-2-(2-fluoro-2-methylpropyl)-3-methyl-2,3,4,9-tetrahydro-1H-pyrido[3,4-*b*]indol-1-yl)phenoxy)ethoxy)ethan-1-ol (2a)

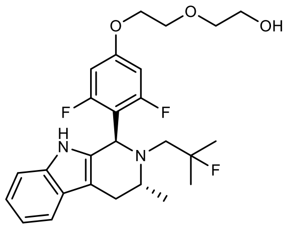

Diethylene glycol (0.47 mL, 4.92 mmol) was added to (1*R*,3*R*)-1-(4-bromo-2,6-difluorophenyl)-2-(2-fluoro-2-methylpropyl)-3-methyl-2,3,4,9-tetrahydro-1*H*-pyrido[3,4-b]indole, HCl (400 mg, 0.82 mmol), cesium carbonate (935 mg, 2.87 mmol) and RockPhos Pd G3 (34.4 mg, 0.04 mmol) in toluene (6.0 mL) at RT under nitrogen. The resulting solution was vacuum degassed and stirred at 90 °C for 4.5 hours. After cooling down to RT, the crude product was purified by flash silica chromatography, elution gradient 5 to 80% EtOAc in heptane. Pure fractions were evaporated to dryness to afford the title compound (191 mg, 49%) as a colourless dry film; ^{1}H NMR (400 MHz, $CDCl_3$, 30°C) 1.09 (3H, d), 1.14 – 1.25 (6H, m), 2.05 (1H, s), 2.39 (1H, dd), 2.60 (1H, m), 2.86 (1H, dd), 3.08 (1H, m), 3.6 – 3.7 (3H, m), 3.71 – 3.76 (2H, m), 3.8 – 3.84 (2H, m), 4.07 (2H, dd), 5.18 (1H, s), 6.39 – 6.45 (2H, m), 7.05 – 7.13 (2H, m), 7.17 – 7.23 (1H, m), 7.47 – 7.53 (1H, m), 7.55 (1H, s); *m/z*: ES- $[M-H]^-$ 475.3.

***tert*-Butyl 2-(2-(2-(3,5-difluoro-4-((1*R*,3*R*)-2-(2-fluoro-2-methylpropyl)-3-methyl-2,3,4,9-tetrahydro-1*H*-pyrido[3,4-*b*]indol-1-yl)phenoxy)ethoxy)ethoxy)acetate (2b)**

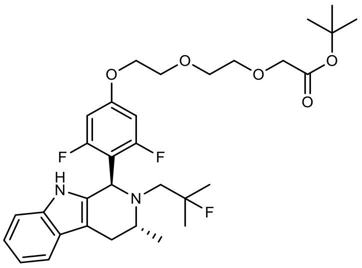

2-(2-(3,5-Difluoro-4-((1*R*,3*R*)-2-(2-fluoro-2-methylpropyl)-3-methyl-2,3,4,9-tetrahydro-1*H*-pyrido[3,4-*b*]indol-1-yl)phenoxy)ethoxy)ethan-1-ol (240 mg, 0.50 mmol) and 60% sodium hydride in mineral oil (14.5 mg, 0.36 mmol) in THF (7 mL) was stirred at RT for 5 minutes under nitrogen. *tert*-butyl 2-bromoacetate (0.089 mL, 0.60 mmol) was added and the resulting suspension was stirred at RT for 2.5 hours under nitrogen. The reaction was incomplete and further 60% sodium hydride in mineral oil (7 mg, 0.17 mmol) was added and the mixture was stirred at RT for a further 1 hour. The reaction was incomplete and further 60% sodium hydride in mineral oil (7 mg, 0.17 mmol) was added and the

suspension was stirred at RT for a further 17 hours. The reaction was incomplete and further *tert*-butyl 2-bromoacetate (20 µL, 0.13 mmol) was added and the suspension was stirred at RT for a further 2 hours. The reaction was incomplete and further *tert*-butyl 2-bromoacetate (20 µL, 0.13 mmol) was added and the suspension was stirred at RT for a further 2 hours. The reaction was incomplete and further *tert*-butyl 2-bromoacetate (20 µL, 0.13 mmol) was added and the suspension was stirred at RT for a further 2 hours. The reaction was incomplete and further *tert*-butyl 2-bromoacetate (20 µL, 0.13 mmol) was added and the suspension was stirred at RT for a further 2 hours. The reaction was incomplete and further 60% sodium hydride in mineral oil (7 mg, 0.17 mmol) and THF (1 mL) was added and the suspension was stirred at 20 °C for a further 1 hour. The reaction mixture was slowly quenched with water (15 mL), extracted with EtOAc (3 x 15 mL), the organic layer was dried over $MgSO_4$, filtered and evaporated. The crude product was purified by flash silica chromatography, elution gradient 5 to 70% EtOAc in heptane. Pure fractions were evaporated to dryness to afford the title compound (161 mg, 54%) as a colourless dry film; ^{1}H NMR (400 MHz, $CDCl_3$, 30°C) 1.10 (3H, dd), 1.15 - 1.26 (6H, m), 1.48 (9H, s), 2.38 (1H, dd), 2.57 - 2.64 (1H, m), 2.87 (1H, dd), 3.06 - 3.14 (1H, m), 3.69 (1H, dd), 3.74 (4H, s), 3.83 - 3.87 (2H, m), 4.02 (2H, d), 4.06 - 4.11 (2H, m), 5.19 (1H, s), 6.41 - 6.46 (2H, m), 7.10 (2H, m), 7.2 - 7.24 (1H, m), 7.47 (1H, s), 7.5 - 7.53 (1H, m); *m/z*: ES+ $[M+H]^+$ 591.4.

2-(2-(2-(3,5-Difluoro-4-((1*R*,3*R*)-2-(2-fluoro-2-methylpropyl)-3-methyl-2,3,4,9-tetrahydro-1*H*-pyrido[3,4-*b*]indol-1-yl)phenoxy)ethoxy)ethoxy)acetic acid (2c)

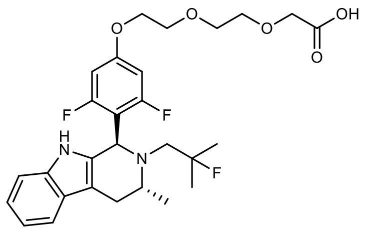

Formic acid (0.50 mL, 13 mmol) was added in one portion to *tert*-butyl 2-(2-(2-(3,5-difluoro-4-((1*R*,3*R*)-2-(2-fluoro-2-methylpropyl)-3-methyl-2,3,4,9-tetrahydro-1*H*-pyrido[3,4-*b*]indol-1-yl)phenoxy)ethoxy)ethoxy)acetate (155 mg, 0.26 mmol) at RT under air. The resulting solution was stirred at RT for 1 hour. The reaction was incomplete so the temperature was increased to 40 °C and the reaction mixture was stirred for further 2.5 hours. The reaction mixture was evaporated to dryness to afford the title compound (141 mg) as a pale yellow dry film that was used in the next step without further purification; *m/z*: ES+ $[M+H]^+$ 535.4.

(2*S*,4*R*)-1-((*S*)-2-(2-(2-(2-(3,5-Difluoro-4-((1*R*,3*R*)-2-(2-fluoro-2-methylpropyl)-3-methyl-2,3,4,9-tetrahydro-1H-pyrido[3,4-*b*]indol-1-yl)phenoxy)ethoxy)ethoxy)acetamido)-3,3-dimethylbutanoyl)-4-hydroxy-*N*-(4-(4-methylthiazol-5-yl)benzyl)pyrrolidine-2-carboxamide (2)

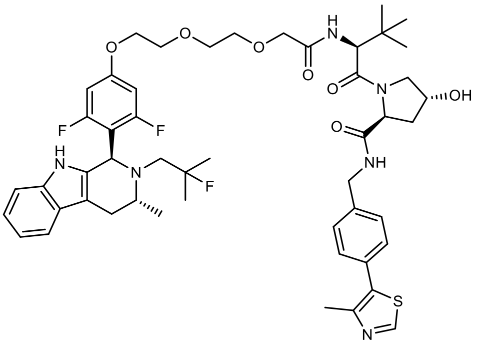

2-(2-(2-(3,5-Difluoro-4-((1*R*,3*R*)-2-(2-fluoro-2-methylpropyl)-3-methyl-2,3,4,9-tetrahydro-1*H*-pyrido[3,4-*b*]indol-1-yl)phenoxy)ethoxy)ethoxy)acetic acid (131 mg, 0.25 mmol), HATU (112 mg, 0.29 mmol), and DIPEA (0.128 mL, 0.74 mmol) were dissolved in DMF (4 mL) at RT and stirred for 15 minutes under nitrogen. (2*S*,4*R*)-1-((*S*)-2-Amino-3,3-dimethylbutanoyl)-4-hydroxy-*N*-(4-(4-methylthiazol-5-yl)benzyl)pyrrolidine-2-carboxamide, HCl (119 mg, 0.25 mmol) was added and the resulting solution was stirred at RT for 3 hours under nitrogen. The reaction was incomplete and further HATU (112 mg, 0.29 mmol), and DIPEA (0.128 mL, 0.74 mmol) were added and the solution was stirred at RT for further 19 hours. The reaction mixture was diluted with EtOAc (10 mL), and washed sequentially with water (10 mL), sat. brine (10 mL), and water (10 mL). The organic layer was dried with $MgSO_4$, filtered and evaporated to afford crude product. The residue was purified by preparative HPLC (Waters CSH C18 OBD column, 30 x 100 mm id, 5 micron particle size),using decreasingly polar mixtures of water (containing 1% by volume of NH_4OH (28-30% in H_2O)) and MeCN as eluents. Fractions containing the desired compound were evaporated to dryness to afford the title compound (46 mg, 20%) as a white solid; ^{1}H NMR (400 MHz, MeOD, 30°C) 1.01 (9H, d), 1.07 – 1.21 (9H, m), 2.08 (1H, m), 2.18 – 2.25 (1H, m), 2.32 – 2.42 (1H, m), 2.44 (3H, s), 2.58 (1H, dd), 2.88 (1H, dd), 3.02 (1H, dd), 3.58 – 3.75 (5H, m), 3.76 – 3.91 (4H, m), 4.01 – 4.06 (2H, m), 4.12 (2H, t), 4.32 (1H, d), 4.47 – 4.59 (3H, m), 4.69 (1H, s), 5.18 (1H, s), 6.45 – 6.53 (2H, m), 6.91 – 7.02 (2H, m), 7.13 – 7.21 (1H, m), 7.36 – 7.47 (5H, m), 8.80 (1H, s), exchangeable protons not observed.; ^{13}C NMR (126 MHz, $CDCl_3$, 17°C) 12.3, 15.9, 24.6, 25.3, 26.4, 27.1, 35.1, 35.7, 41.0, 43.3, 50.6, 51.2, 56.8, 56.9, 57.1, 58.4, 67.6, 69.6, 70.1, 70.3, 70.8, 71.2, 97.3, 98.7, 108.0, 109.8, 110.8, 118.1, 118.9, 121.0, 127.6, 128.2, 129.5, 130.9, 131.6, 132.6, 136.3, 138.0, 148.4, 150.4, 159.6, 162.7, 170.5, 170.7, 171.4; *m/z*: ES+ $[M+H]^+$ 947.6; LCMS purity = 100% ; ESI-HRMS calculated for $C_{50}H_{62}F_3N_6O_7S$ $[M+H]^+$ = 947.4347, measured 947.4343.

3,5-Difluoro-4-((1*R*,3*R*)-2-(2-fluoro-2-methylpropyl)-3-methyl-2,3,4,9-tetrahydro-1*H*-pyrido[3,4-b]indol-1-yl)phenol (3a)

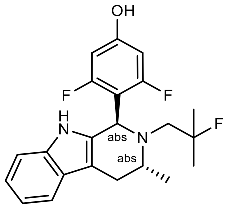

A solution of (*R*)-*N*-(1-(1*H*-indol-3-yl)propan-2-yl)-2-fluoro-2-methylpropan-1-amine[8] (4.95 g, 19.93 mmol) in toluene (10 mL) was added to a stirred solution of 2,6-difluoro-4-hydroxybenzaldehyde (3.18 g, 20.13 mmol) in toluene (120 mL) and AcOH (9 mL) at RT under air. The resulting solution was stirred at 80 °C for 18 hours. The reaction was cooled to RT and stirred for 18 hours. The reaction was warmed again to 80 °C for 8 hours. The reaction mixture was cooled to RT. The crude product was purified by ion exchange chromatography, using an SCX column. The desired product was eluted from the column using 1M NH_3/MeOH and pure fractions were evaporated to dryness to crude product. The crude product was purified by flash silica chromatography, elution gradient 0 to 30% EtOAc in DCM. Pure fractions were evaporated to dryness to afford the title compound (4.60 g, 59%) as an orange solid; ^{1}H NMR (400 MHz, $CDCl_3$, 30 °C) 1.10 (3H, d), 1.18 (3H, d), 1.21 - 1.26 (3H, m), 2.39 (1H, dd), 2.60 (1H, m), 2.86 (1H, dd), 3.08 (1H, m), 3.61 - 3.72 (1H, m), 5.18 (1H, s), 6.32 - 6.38 (2H, m), 7.06 - 7.14 (2H, m), 7.21 - 7.24 (1H, m), 7.41 (1H, s), 7.47 - 7.54 (1H, m); *m/z*: ES+ $[M+H]^+$ 389.2.

Ethyl 2-(2-(2-(2-(3,5-difluoro-4-((1*R*,3*R*)-2-(2-fluoro-2-methylpropyl)-3-methyl-2,3,4,9-tetrahydro-1*H*-pyrido[3,4-b]indol-1-yl)phenoxy)ethoxy)ethoxy)ethoxy)acetate (3b)

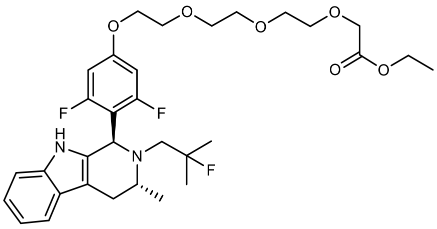

DIAD (1.67 mL, 8.50 mmol) was added dropwise to 3,5-difluoro-4-((1*R*,3*R*)-2-(2-fluoro-2-methylpropyl)-3-methyl-2,3,4,9-tetrahydro-1*H*-pyrido[3,4-b]indol-1-yl)phenol (1.65 g, 4.25 mmol), triphenylphosphine (2.23 g, 8.50 mmol) and ethyl 2-(2-(2-(2-hydroxyethoxy)ethoxy)ethoxy)acetate[15] (1.51 g, 6.37 mmol) in DCM (50 mL) cooled to 0 °C over a period of 2 minutes under nitrogen. The resulting solution was stirred at 0 °C for 45 minutes and warmed to RT for 1 hour. The reaction was incomplete and further triphenylphosphine (0.56 g, 2.13 mmol) and DIAD (0.42 mL, 2.13 mmol) were

added and the solution was stirred at RT for a further 30 minutes. The crude reaction mixture was purified by ion exchange chromatography, using an SCX column. The desired product was eluted from the column using 1M NH_3/MeOH and pure fractions were evaporated to dryness to afford crude product. The crude product was purified by flash silica chromatography, elution gradient 10 to 50% EtOAc in heptane. Pure fractions were evaporated to dryness to afford the title compound (1.09 g, 42%) as a colourless gum; ^{1}H NMR (400 MHz, DMSO-d6, 30 °C) 1.05 (3H, d), 1.15 - 1.3 (9H, m), 2.35 (1H, dd), 2.53 - 2.61 (1H, m), 2.78 - 2.95 (2H, m), 3.48 - 3.62 (9H, m), 3.71 - 3.77 (2H, m), 4.08 - 4.16 (6H, m), 5.13 (1H, s), 6.68 (2H, d), 6.89 - 7.07 (2H, m), 7.15 - 7.24 (1H, m), 7.40 (1H, d), 10.50 (1H, s).*m/z*: ES+ $[M+H]^+$ 607.5.

2-(2-(2-(2-(3,5-Difluoro-4-((1*R*,3*R*)-2-(2-fluoro-2-methylpropyl)-3-methyl-2,3,4,9-tetrahydro-1*H*-pyrido[3,4-b]indol-1-yl)phenoxy)ethoxy)ethoxy)ethoxy)acetic acid (3c)

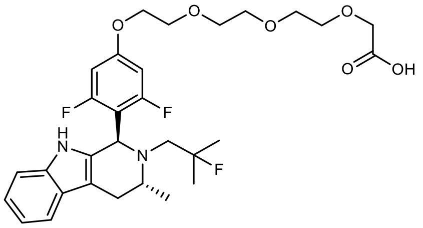

Lithium hydroxide hydrate (0.151 g, 3.59 mmol) was added in one portion to ethyl 2-(2-(2-(2-(3,5-difluoro-4-((1*R*,3*R*)-2-(2-fluoro-2-methylpropyl)-3-methyl-2,3,4,9-tetrahydro-1*H*-pyrido[3,4-*b*]indol-1-yl)phenoxy)ethoxy)ethoxy)ethoxy)acetate (1.09 g, 1.80 mmol) in THF (15 mL) and water (5 mL) at RT. The resulting solution was stirred at RT for 30 minutes and the solvent removed under reduced pressure. The resulting mixture was acidified with 2M aq. HCl and extracted into EtOAc (2 x 10 mL). The organic extracts were washed with sat. brine (5 mL) and evaporated to afford the title compound (0.88 g, 85%) that was used without further purification; ^{1}H NMR (400 MHz, DMSO-d6, 30 °C) 1.05 (3H, d), 1.11 (3H, s), 1.23 (2H, d), 2.38 (1H, d), 2.58 (1H, d), 2.78 - 2.95 (2H, m), 3.52 - 3.56 (5H, m), 3.58 (5H, dd), 3.71 - 3.76 (2H, m), 4.02 (2H, s), 4.12 (2H, s), 5.13 (1H, s), 6.68 (2H, d), 6.97 (2H, m), 7.19 (1H, d), 7.40 (1H, d), 10.50 (1H, s), 12.49 (1H, s); *m/z*: ES+ $[M+H]^+$ 579.3.

(2*S*,4*R*)-1-((*S*)-2-(*tert*-Butyl)-14-(3,5-difluoro-4-((1*R*,3*R*)-2-(2-fluoro-2-methylpropyl)-3-methyl-2,3,4,9-tetrahydro-1*H*-pyrido[3,4-*b*]indol-1-yl)phenoxy)-4-oxo-6,9,12-trioxa-3-azatetradecanoyl)-4-hydroxy-*N*-(4-(4-methylthiazol-5-yl)benzyl)pyrrolidine-2-carboxamide (3)

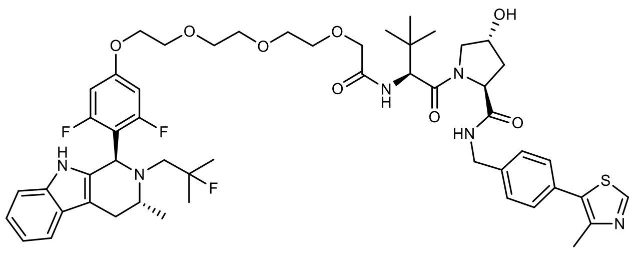

HATU (1.95 g, 5.14 mmol) was added portionwise to 2-(2-(2-(2-(3,5-difluoro-4-((1*R*,3*R*)-2-(2-fluoro-2-methylpropyl)-3-methyl-2,3,4,9-tetrahydro-1*H*-pyrido[3,4-b]indol-1-yl)phenoxy)ethoxy)ethoxy)ethoxy)acetic acid (1.98 g, 3.42 mmol), (2*S*,4*R*)-1-((*S*)-2-amino-3,3-dimethylbutanoyl)-4-hydroxy-*N*-(4-(4-methylthiazol-5-yl)benzyl)pyrrolidine-2-carboxamide, HCl (1.48 g, 3.25 mmol) and triethylamine (1.91 mL, 13.71 mmol) in DMF (10 mL) at RT under nitrogen. The resulting mixture was stirred at RT for 30 minutes . The reaction mixture was diluted with EtOAc (25 mL), and washed sequentially with sat. $NaHCO_3$ (25 mL), water (25 mL), and sat. brine (25 mL). The organic layer was dried with Na_2SO_4, filtered and evaporated to afford crude product. The crude product was purified by flash silica chromatography, elution gradient 0 to 10% MeOH in DCM. Pure fractions were evaporated to dryness to afford a yellow oil. The samples were dissolved in MeOH and purified using the SFC conditions: Column: Princeton Diol, 30 x 250 mm, 5 micron; Mobile phase: A = MeOH + 0.1% NH_3, B = $scCO_2$; Gradient: 20-30% A over 8 minutes; Flow rate: 100 mL/min; BPR: 120 bar; Column temperature: 40 °C. The title compound (1.1 g, 32%) was obtained as a pale yellow solid; ^{1}H NMR (400 MHz, $CDCl_3$, 30 °C) 0.94 (s, 9H), 1.10 (d, *J* = 6.5 Hz, 3H), 1.20 (dd, *J* = 21.5, 16.5 Hz, 6H), 2.06 (ddt, *J* = 12.9, 8.4, 2.1 Hz, 1H), 2.39 (dd, *J* = 25.3, 15.0 Hz, 1H), 2.48 (s, 3H), 2.53 (m, 1H), 2.60 (dd, *J* = 14.9, 3.9 Hz, 1H), 2.84 (dd, *J* = 19.5, 15.0 Hz, 1H), 2.97 (s, 1H, OH), 3.08 (m, 1H), 3.59 (dd, *J* = 11.3, 3.8 Hz, 1H), 3.62 – 3.73 (m, 10H), 3.78 (t, *J* = 4.7 Hz, 2H), 3.93 (dd (AB), 2H), 4.01 – 4.09 (m, 3H), 4.31 (dd, *J* = 14.9, 5.2 Hz, 1H), 4.50 (m, 1H), 4.51 (d, *J* = 8.3 Hz, 1H), 4.55 (dd, *J* = 15.0, 6.6 Hz, 1H), 4.70 (t, *J* = 7.8 Hz, 1H), 5.19 (s, 1H), 6.38 (m, 2H), 7.08 (td, *J* = 7.3, 6.7, 4.0 Hz, 2H), 7.23 (m, 1H), 7.31 (m, 1H, NH), 7.33 (m, 1H, NH), 7.34 (q, *J* = 8.4 Hz, 4H), 7.51 (m, 1H), 8.35 (s, 1H, NH), 8.63 (s, 1H).; ^{13}C NMR (126 MHz, $CDCl_3$, 30 °C) 12.21, 15.96, 24.95, 26.40, 27.09, 34.95, 35.59, 43.21, 50.56, 51.16, 56.59, 56.88, 57.11, 58.32, 67.96, 69.36, 70.11, 70.27, 70.75, 97.26, 98.65, 107.98, 109.45, 110.77, 118.10, 118.97, 121.10, 127.46, 128.21, 129.47, 131.29, 132.29, 136.20, 137.85, 137.91, 148.40, 150.26, 159.46, 162.60, 170.37, 170.68, 171.07; *m/z*: ES+ $[M+H]^+$ 991.7; LCMS purity = 100% ; ESI-HRMS calculated for $C_{52}H_{66}F_3N_6O_8S$ $[M+H]^+$ = 991.4609, measured 991.4584.

2-(2-(2-(2-(3,5-Difluoro-4-((1*R*,3*R*)-2-(2-fluoro-2-methylpropyl)-3-methyl-2,3,4,9-tetrahydro-1*H*-pyrido[3,4-b]indol-1-yl)phenoxy)ethoxy)ethoxy)ethoxy)ethan-1-ol (4a)

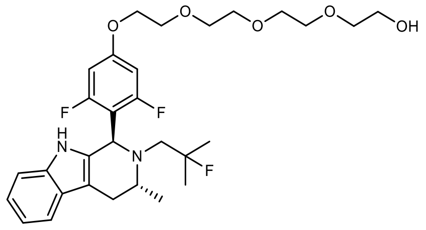

RockPhos Pd G3 (86 mg, 0.10 mmol) was added in one portion to a degassed mixture of 2,2'-((oxybis(ethane-2,1-diyl))bis(oxy))bis(ethan-1-ol) (1.77 mL, 10.25 mmol), (1*R*,3*R*)-1-(4-bromo-2,6-difluorophenyl)-2-(2-fluoro-2-methylpropyl)-3-methyl-2,3,4,9-tetrahydro-1*H*-pyrido[3,4-b]indole, HCl (500 mg, 1.03 mmol) 4 Å molecular sieves (100 mg), and cesium carbonate (1.17g, 3.59 mmol) in toluene (7.5 mL) at RT under nitrogen. The resulting mixture was stirred at 90 °C for 4 hours. The reaction was allowed to cool to RT and diluted with DCM (10 mL), filtered and evaporated to afford crude product as a orange gum. The crude product was purified by flash silica chromatography, elution gradient 0 to 100% EtOAc in heptane. Pure fractions were evaporated to dryness to afford the title compound (261 mg, 45%) as an orange gum; ^{1}H NMR (400 MHz, $CDCl_3$, 30 °C) 1.10 (3H, d), 1.20 (6H, dd), 2.31 - 2.47 (2H, m), 2.53 - 2.67 (1H, m), 2.86 (1H, dd), 3.04 - 3.14 (1H, m), 3.55 - 3.62 (2H, m), 3.63 - 3.73 (11H, m), 3.83 (2H, dd), 4.05 - 4.12 (2H, m), 5.18 (1H, s), 6.39 - 6.5 (2H, m), 7.03 - 7.14 (2H, m), 7.18 - 7.24 (1H, m), 7.51 (1H, dd), 7.59 (1H, s); *m/z*: ES+ $[M+H]^+$ 565.3.

***tert*-Butyl 14-(3,5-difluoro-4-((1*R*,3*R*)-2-(2-fluoro-2-methylpropyl)-3-methyl-2,3,4,9-tetrahydro-1*H*-pyrido[3,4-b]indol-1-yl)phenoxy)-3,6,9,12-tetraoxatetradecanoate (4b)**

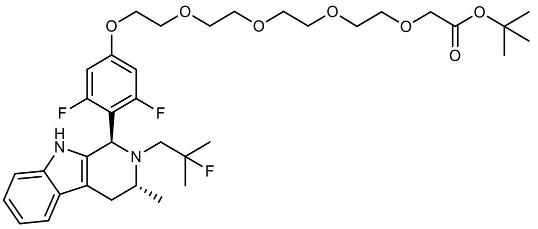

60% Sodium hydride in mineral oil (8.50 mg, 0.21 mmol) was added in one portion to 2-(2-(2-(2-(3,5-difluoro-4-((1*R*,3*R*)-2-(2-fluoro-2-methylpropyl)-3-methyl-2,3,4,9-tetrahydro-1*H*-pyrido[3,4-b]indol-1-yl)phenoxy)ethoxy)ethoxy)ethoxy)ethan-1-ol (100 mg, 0.18 mmol), in THF (5 mL) at RT under nitrogen. *tert*-butyl 2-bromoacetate (0.031 mL, 0.21 mmol) was added after 10 minutes and the reaction was stirred at RT for 2 hours. Further 60% sodium hydride in mineral oil (8.5 mg, 0.21 mmol) was added and the reaction stirred for 1 hour. The reaction was slowly quenched with water and

extracted with EtOAc (2 x 20 mL). The combined organics were washed with brine (20 mL), dried over anhydrous $MgSO_4$, filtered and evaporated under reduced pressure. The crude product was purified by flash silica chromatography, elution gradient 0 to 100% EtOAc in heptane. Pure fractions were evaporated to dryness to afford the title compound (49 mg, 41%) as a colourless oil; ^1H NMR (400 MHz, $CDCl_3$, 30 °C) 1.09 (3H, d), 1.17 (3H, d), 1.23 (3H, d), 1.46 (9H, s), 2.38 (1H, dd), 2.60 (1H, m), 2.86 (1H, dd), 3.05 - 3.14 (1H, m), 3.63 - 3.72 (13H, m), 3.83 (2H, dd), 3.99 (2H, s), 4.05 - 4.1 (2H, m), 5.18 (1H, s), 6.38 - 6.48 (2H, m), 7.03 - 7.14 (2H, m), 7.19 - 7.25 (1H, m), 7.47 - 7.57 (2H, m); *m/z*: ES+ $[M+H]^+$ 679.4.

14-(3,5-Difluoro-4-((1*R*,3*R*)-2-(2-fluoro-2-methylpropyl)-3-methyl-2,3,4,9-tetrahydro-1*H*-pyrido[3,4-b]indol-1-yl)phenoxy)-3,6,9,12-tetraoxatetradecanoic acid (4c)

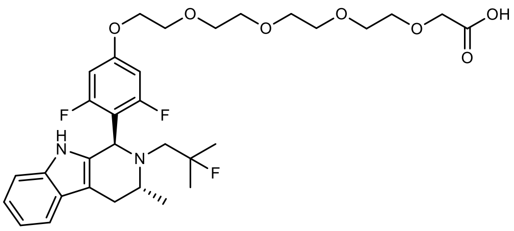

tert-Butyl 14-(3,5-difluoro-4-((1*R*,3*R*)-2-(2-fluoro-2-methylpropyl)-3-methyl-2,3,4,9-tetrahydro-1*H*-pyrido[3,4-b]indol-1-yl)phenoxy)-3,6,9,12-tetraoxatetradecanoate (49 mg, 0.07 mmol) was dissolved in formic acid (0.5 mL) at RT under air. The resulting mixture was stirred at RT for 5 hours and concentrated. The residue was re-dissolved in formic acid (0.5 mL) and stirred for a further 2 hours. The solvent was removed under reduced pressure to afford the title compound as a yellow oil that was used in the next step without further purification; *m/z*: ES+ $[M+H]^+$ 623.3.

(2*S*,4*R*)-1-((*S*)-2-(*tert*-Butyl)-17-(3,5-difluoro-4-((1*R*,3*R*)-2-(2-fluoro-2-methylpropyl)-3-methyl-2,3,4,9-tetrahydro-1*H*-pyrido[3,4-*b*]indol-1-yl)phenoxy)-4-oxo-6,9,12,15-tetraoxa-3-azaheptadecanoyl)-4-hydroxy-*N*-(4-(4-methylthiazol-5-yl)benzyl)pyrrolidine-2-carboxamide (4)

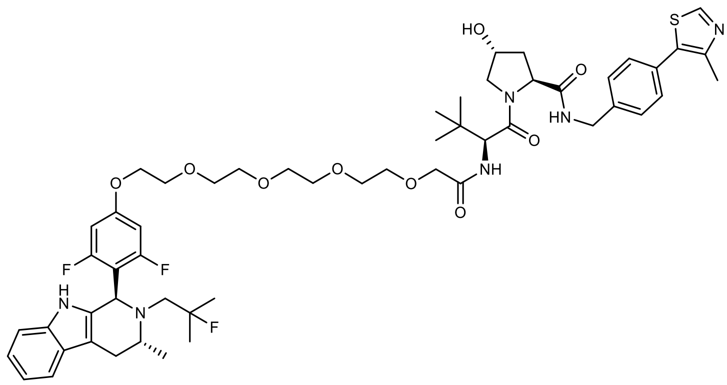

HATU (41 mg, 0.11 mmol) was added portionwise to 14-(3,5-difluoro-4-((1*R*,3*R*)-2-(2-fluoro-2-methylpropyl)-3-methyl-2,3,4,9-tetrahydro-1*H*-pyrido[3,4-b]indol-1-yl)phenoxy)-3,6,9,12-tetraoxatetradecanoic acid (45 mg, 0.07 mmol), (2*S*,4*R*)-1-((*S*)-2-amino-3,3-dimethylbutanoyl)-4-hydroxy-*N*-(4-(4-methylthiazol-5-yl)benzyl)pyrrolidine-2-carboxamide, HCl (33.8 mg, 0.07 mmol) and triethylamine (40 µL, 0.29 mmol) in DMF (1.4 mL) at RT under nitrogen. The resulting mixture was stirred at RT for 30 minutes. Further HATU (41.2 mg, 0.11 mmol) and triethylamine (40 µL, 0.29 mmol) were added and the reaction stirred at RT for 1 hour. The reaction mixture was diluted with EtOAc (50 mL), and washed sequentially with water (50 mL) and sat. brine (25 mL). The organic layer was dried with $MgSO_4$, filtered and evaporated to afford crude product. The crude residue was purified by preparative HPLC (Waters XSelect CSH C_{18} column, 30 x 100 mm id, 5 micron particle size), using decreasingly polar mixtures of water (containing 0.1% formic acid) and MeCN as eluents. Fractions containing the desired compound were evaporated to dryness to afford the title compound (9 mg, 12%); ^{1}H NMR (400 MHz, MeOD, 30 °C) 1.02 (9H, d), 1.21 (9H, d), 1.41 - 1.55 (1H, m), 2.08 (1H, m), 2.16 - 2.26 (1H, m), 2.46 (4H, s), 2.63 (1H, d), 2.91 (1H, s), 3.04 (1H, s), 3.59 - 3.72 (14H, m), 3.76 - 3.88 (4H, m), 4.01 (2H, d), 4.12 (2H, d), 4.34 (1H, dd), 4.44 - 4.61 (3H, m), 4.69 (1H, d), 5.20 (1H, s), 6.55 (2H, s), 6.98 (2H, s), 7.19 (1H, d), 7.36 - 7.48 (5H, m), 7.62 (1H, d), 8.58 (1H, t), 8.84 (1H, d); *m/z*: ES+ $[M+H]^+$ 1035.5; LCMS purity = 98%; ESI-HRMS calculated for $C_{54}H_{70}F_3N_6O_9S$ $[M+H]^+$ = 1035.4872, measured 1035.4830.

2-(3-(3-(Benzyloxy)propoxy)propoxy)tetrahydro-2*H*-pyran (5a)

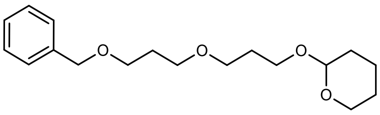

Tetrabutylammonium hydrogen sulfate (0.951 g, 2.80 mmol) was added in one portion to 3-(benzyloxy)propan-1-ol (2.96 mL, 18.7 mmol) and 2-(3-bromopropoxy)tetrahydro-2*H*-pyran (5.0 g, 22.4 mmol) in 50% sodium hydroxide solution (12 mL) at rt under air. The resulting mixture was stirred at 70 °C for 18 hours. The cooled reaction mixture was diluted with water (20 mL) and

extracted with EtOAc (3 x 25 mL). The combined organic extracts were washed with water (20 mL), sat. brine solution (20 mL), dried over $MgSO_4$, filtered and evaporated to afford crude product as a yellow oil. The crude product was purified by flash silica chromatography, elution gradient 0 to 10% EtOAc in heptane to afford the title compound (3.42 g, 59%) as a colourless oil; ^{1}H NMR (400 MHz, $CDCl_3$, 30°C) 1.49 – 1.61 (4H, m), 1.65 – 1.73 (1H, m), 1.78 – 1.92 (5H, m), 3.43 – 3.6 (8H, m), 3.75 – 3.9 (2H, m), 4.50 (2H, s), 4.54 – 4.59 (1H, m), 7.23 – 7.28 (1H, m), 7.32 (4H, d).

3-(3-(Benzyloxy)propoxy)propan-1-ol (5b)

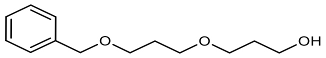

2-(3-(3-(Benzyloxy)propoxy)propoxy)tetrahydro-2*H*-pyran (3.42 g, 11.1 mmol) was dissolved in MeOH (30 mL) and 1M aq. HCl (15 mL) was added. The resulting mixture was stirred at RT for 1 hour. The reaction mixture was diluted with water (100 mL), and extracted with EtOAc (3 x 100 mL). The combined organics were washed with sat. brine (50 mL). The organic layer was dried with $MgSO_4$, filtered and evaporated to afford the title compound (2.58 g) that was used directly in the next step without further purification; ^{1}H NMR (400 MHz, $CDCl_3$, 30°C) 1.78 – 1.92 (4H, m), 2.36 (1H, s), 3.55 (4H, td), 3.61 (2H, t), 3.76 (2H, t), 4.50 (2H, s), 7.27 – 7.31 (1H, m), 7.31 – 7.38 (4H, m); *m/z*: ES+ $[M+H]^+$ 225.2.

Ethyl 2-(3-(3-(benzyloxy)propoxy)propoxy)acetate (5c)

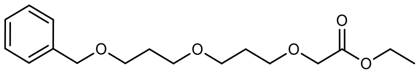

Ethyl 2-diazoacetate (3.42 mL, 27.9 mmol) in DCM (10 mL) was added slowly to 3-(3-(benzyloxy)propoxy)propan-1-ol (2.50 g, 11.2 mmol) and diacetoxyrhodium (0.25 g, 0.56 mmol) in DCM (30 mL) at RT over a period of 1 hour under nitrogen. The resulting solution was stirred for 18 hours. The mixture was diluted with DCM (50 mL) and washed with water (3 x 50 mL). The organic layer was collected and filtered through a phase separating cartridge then evaporated to dryness. The crude product was purified by flash silica chromatography, elution gradient 0 to 10% EtOAc in heptane to afford the title compound (2.27 g, 66%) as a colourless liquid; ^{1}H NMR (400 MHz, $CDCl_3$, 30°C) 1.29 (3H, dt), 1.88 (4H, p), 3.48 – 3.63 (8H, m), 4.05 (2H, s), 4.18 – 4.27 (2H, m), 4.50 (2H, s), 7.26 – 7.29 (1H, m), 7.33 (4H, d); *m/z*: ES+ $[M+H]^+$ 311.2.

Ethyl 2-(3-(3-hydroxypropoxy)propoxy)acetate (5d)

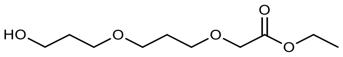

Ethyl 2-(3-(3-(benzyloxy)propoxy)propoxy)acetate (2.20 g, 7.09 mmol) and 10% palladium on carbon (0.075 g, 0.71 mmol) in ethanol (14 mL) were stirred under an atmosphere of hydrogen at RT for 4 hours. The reaction mixture was filtered, washing with EtOH then the solvent was evaporated to afford crude product. The crude product was purified by flash silica chromatography, elution gradient 0 to 100% EtOAc in heptane to afford the title compound (1.11 g, 71%) as a colourless oil; ^{1}H NMR (400 MHz, $CDCl_3$, 30°C) 1.29 (3H, t), 1.83 (2H, p), 1.89 (2H, p), 2.42 (1H, t), 3.57 (2H, t), 3.62 (4H, td), 3.77 (2H, q), 4.06 (2H, s), 4.22 (2H, q).

Ethyl 2-(3-(3-(3,5-difluoro-4-((1*R*,3*R*)-2-(2-fluoro-2-methylpropyl)-3-methyl-2,3,4,9-tetrahydro-1*H*-pyrido[3,4-b]indol-1-yl)phenoxy)propoxy)propoxy)acetate (5e)

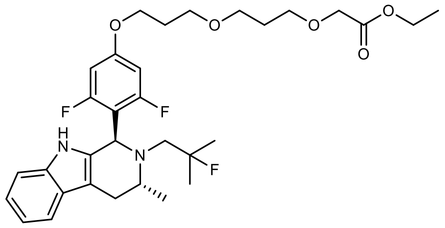

DIAD (0.10 mL, 0.51 mmol) was added dropwise over 15 minutes to a stirred mixture of 3,5-difluoro-4-((1*R*,3*R*)-2-(2-fluoro-2-methylpropyl)-3-methyl-2,3,4,9-tetrahydro-1*H*-pyrido[3,4-b]indol-1-yl)phenol (100 mg, 0.26 mmol), ethyl 2-(3-(3-hydroxypropoxy)propoxy)acetate (113 mg, 0.51 mmol) and triphenylphosphine (135 mg, 0.51 mmol) in DCM (6 mL) at 0 °C. The resulting mixture was stirred at RT for 1 hour. DCM (50 mL) and water (25 mL) were added and the layers were separated. The DCM layer was passed through a phase separating cartridge and concentrated to give the crude product as an orange oil. The crude product was purified by flash silica chromatography, elution gradient 0 to 25% EtOAc in heptane to afford the title compound (141 mg, 93%) as a pale yellow gum; ^{1}H NMR (400 MHz, $CDCl_3$, 30°C) 1.10 (3H, d), 1.15 – 1.25 (6H, m), 1.25 – 1.29 (3H, t), 1.87 (2H, p), 1.97 – 2.07 (2H, m), 2.39 (1H, dd), 2.60 (1H, dd), 2.86 (1H, dd), 3.09 (1H, dd), 3.51 – 3.61 (6H, m), 3.64 – 3.73 (1H, m), 3.99 – 4.04 (3H, m), 4.98 (2H, hept), 5.19 (1H, s), 6.28 (1H, s), 6.37 – 6.45 (2H, m), 7.05 – 7.14 (2H, m), 7.18 – 7.24 (1H, m), 7.51 (1H, dd), 7.56 (1H, s); *m/z*: ES+ $[M+H]^+$ 591.4.

2-(3-(3-(3,5-Difluoro-4-((1*R*,3*R*)-2-(2-fluoro-2-methylpropyl)-3-methyl-2,3,4,9-tetrahydro-1*H*-pyrido[3,4-b]indol-1-yl)phenoxy)propoxy)propoxy)acetic acid (5f)

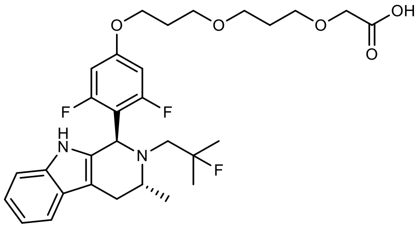

Lithium hydroxide monohydrate (20 mg, 0.48 mmol) was added in one portion to ethyl 2-(3-(3-(3,5-difluoro-4-((1*R*,3*R*)-2-(2-fluoro-2-methylpropyl)-3-methyl-2,3,4,9-tetrahydro-1*H*-pyrido[3,4-b]indol-1-yl)phenoxy)propoxy)propoxy)acetate (141 mg, 0.24 mmol) in THF (1 mL) and water (0.3 mL) at RT under air. The resulting solution was stirred for 30 minutes. The reaction mixture was diluted with water (10 mL) then was acidified with 2M HCl and extracted into EtOAc (50 mL). The organic layer was washed with brine (15 mL) and evaporated to afford the title compound (134 mg, 100%) as a yellow gum which was used in the next step without further purification; *m/z*: ES+ $[M+H]^+$ 563.3.

(2*S*,4*R*)-1-((*S*)-2-(2-(3-(3-(3,5-Difluoro-4-((1*R*,3*R*)-2-(2-fluoro-2-methylpropyl)-3-methyl-2,3,4,9-tetrahydro-1*H*-pyrido[3,4-b]indol-1-yl)phenoxy)propoxy)propoxy)acetamido)-3,3-dimethylbutanoyl)-4-hydroxy-*N*-(4-(4-methylthiazol-5-yl)benzyl)pyrrolidine-2-carboxamide (5)

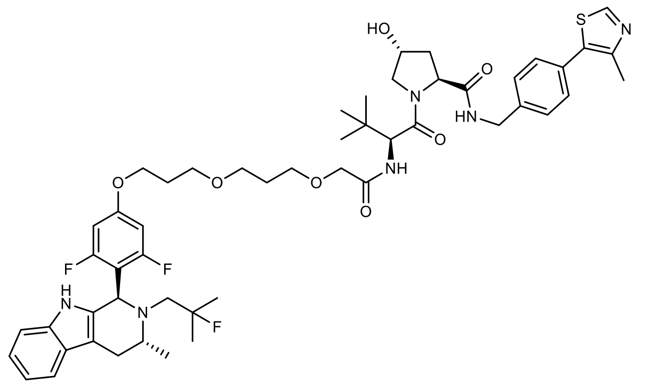

HATU (136 mg, 0.36 mmol) was added in one portion to (2*S*,4*R*)-1-((*S*)-2-amino-3,3-dimethylbutanoyl)-4-hydroxy-*N*-(4-(4-methylthiazol-5-yl)benzyl)pyrrolidine-2-carboxamide, HCl (111 mg, 0.24 mmol), 2-(3-(3-(3,5-difluoro-4-((1*R*,3*R*)-2-(2-fluoro-2-methylpropyl)-3-methyl-2,3,4,9-tetrahydro-1*H*-pyrido[3,4-b]indol-1-yl)phenoxy)propoxy)propoxy)acetic acid (134 mg, 0.24 mmol) and triethylamine (0.13 mL, 0.95 mmol) in DMF (5 mL) at RT under nitrogen. The resulting mixture was stirred for 30 minutes. The reaction mixture was diluted with EtOAc (50 mL), and washed sequentially with water (50 mL) and sat. brine (25 mL). The organic layer was dried with $MgSO_4$, filtered and evaporated to afford crude product. The crude product was purified by preparative HPLC (Waters CSH C18 OBD column, 30 x 100 mm id, 5 micron particle size),using decreasingly polar mixtures of water (containing 1% by volume of NH_4OH (28-30% in H_2O)) and MeCN as eluents to afford the title compound (106 mg, 46%) as a white solid; ^1H NMR (500 MHz, $CDCl_3$, 30°C) 0.94 (s, 9H), 1.09 (d, *J* = 6.5 Hz, 3H), 1.21 (dd, *J* = 21.6, 18.8 Hz, 6H), 1.84 (tt, *J* = 5.8, 2.9 Hz, 2H), 1.99 (p, *J* = 5.9 Hz, 2H), 2.09 (dddd, *J* = 13.0, 8.2, 3.5, 1.5 Hz, 1H), 2.42 (dd, *J* = 24.4, 15.0 Hz, 1H), 2.50 (s, 3H), 2.52 – 2.66 (m, 2H), 2.78 (d, *J* = 4.3 Hz, 1H, OH), 2.83 (dd, *J* = 21.2, 15.2 Hz, 1H), 3.05 (dd, J=14.7, 3.7, 1H), 3.45 – 3.55 (m, 5H), 3.57 (br t, 2H), 3.65 (m, 1H), 3.65 (dd, *J* = 11.1, 4.1 Hz, 2H), 3.80 (d, *J* = 15.4 Hz, 1H), 3.97 (td, *J* = 6.0, 2.8 Hz, 2H), 4.02 (dt, *J* = 11.5, 1.9 Hz, 1H), 4.34 (dd, *J* = 14.9, 5.3 Hz, 1H), 4.51

– 4.61 (m, 3H), 4.73 (t, *J* = 7.7 Hz, 1H), 5.20 (s, 1H), 6.28 – 6.41 (m, 2H), 7.01 – 7.13 (m, 2H), 7.16 (d, *J* = 9.0 Hz, 1H, NH), 7.19 – 7.26 (m, 2H), 7.31 – 7.42 (m, 4H), 7.47 – 7.56 (m, 1H), 8.65 (s, 1H), 8.67 (s, 1H, NH); ^{13}C NMR (126 MHz, $CDCl_3$, 30°C) δ 13.03, 16.23, 25.18, 26.64, 27.30, 29.45, 29.95, 35.47, 35.93, 43.54, 51.11, 51.27, 56.78, 56.98, 57.02, 58.75, 65.20, 66.77, 67.39, 68.75, 70.12, 70.49, 97.76, 98.62, 108.42, 109.87, 111.00, 118.33, 119.22, 121.24, 127.82, 128.55, 129.77, 131.17, 131.66, 132.85, 136.51, 138.16, 148.58, 150.44, 160.20, 162.98, 162.98, 170.49, 170.80, 171.57; *m/z*: ES+ $[M+H]^+$ 975.6; LCMS purity = 100%; ESI-HRMS calculated for $C_{52}H_{65}F_3N_6O_7S$ $[M+H]^+$ = 975.4666, measured 975.4673.

tert-Butyl 4-(2-(2-ethoxy-2-oxoethoxy)ethyl)piperazine-1-carboxylate (6a)

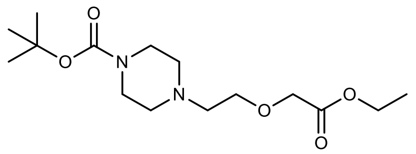

Rhodium(II) acetate (0.27 g, 0.62 mmol) was added to a stirred solution of *tert*-butyl 4-(2-hydroxyethyl)piperazine-1-carboxylate (1.42 g, 6.17 mmol) in DCM (30 mL) cooled to 0°C. Ethyl 2-diazoacetate (0.938 g, 7.40 mmol) in DCM (5 mL) was added over a period of 5 minute under nitrogen. The resulting suspension was stirred at room temperature for 4 hours. Water (20 mL) was added. The organic layer was separated, washed with brine (15 mL), and concentrated in vacuo. The resulting residue was purified by flash silica chromatography, elution gradient 0 to 10% MeOH in DCM. Product fractions were concentrated under reduced pressure to afford the title compound (0.650 g, 33%) as a brown oil; ^{1}H NMR (400 MHz, $CDCl_3$) 1.28 (t, J = 7.1 Hz, 3H), 1.46 (s, 9H), 2.41 – 2.53 (m, 4H), 2.64 (t, J = 5.6 Hz, 2H), 3.4 – 3.49 (m, 4H), 3.69 (t, J = 5.6 Hz, 2H), 4.10 (s, 2H), 4.21 (q, J = 7.1 Hz, 2H); *m/z*: ES+ $[M+H]^+$ 317.0.

Ethyl 2-(2-(piperazin-1-yl)ethoxy)acetate (6b)

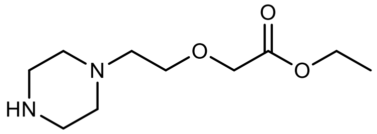

A solution of 4 M HCl in dioxane (1.2 mL, 4.8 mmol) in was added to a stirred solution of tert-butyl 4-(2-(2-ethoxy-2-oxoethoxy)ethyl)piperazine-1-carboxylate (0.7 g, 2.21 mmol) inn DCM (10 mL) at 0°C. The resulting solution was stirred at room temperature for 8 hours. The reaction solution was concentrated under reduced pressue to afford the title compound (0.520 g, 93 %) as a colourless gum; m/z: ES+ $[M+H]^+$=217.

(1*R*,3*R*)-1-(4-(2-Bromoethoxy)-2,6-difluorophenyl)-2-(2-fluoro-2-methylpropyl)-3-methyl-2,3,4,9-tetrahydro-1*H*-pyrido[3,4-b]indole (6c)

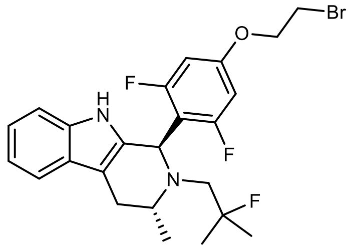

DIAD (0.55 ml, 2.8 mmol) was added dropwise to a stirred solution of 3,5-difluoro-4-((1*R*,3*R*)-2-(2-fluoro-2-methylpropyl)-3-methyl-2,3,4,9-tetrahydro-1*H*-pyrido[3,4-b]indol-1-yl)phenol (544 mg, 1.40 mmol), 2-bromoethan-1-ol (438 mg, 3.50 mmol) and triphenylphosphine (735 mg, 2.80 mmol) in DCM (7 mL) at 5 °C. The resulting mixture was stirred at 5 °C for 30 minutes and then at 21 °C for 12 hour. The reaction mixture was diluted with DCM (20 mL) and water (40 mL). After separation, the organic fraction were concentrated under reduced pressure. The crude product was purified by flash silica chromatography, elution gradient 0 to 100% EtOAc in heptane. Pure fractions were evaporated to dryness to afford the title compound (250 mg, 36.0 %) as a pale yellow oil; ^{1}H NMR (500 MHz, $CDCl_3$) 1.10 (d, J = 6.6 Hz, 3H), 1.18 (d, J = 12.7 Hz, 6H), 2.39 (dd, J = 14.7, 25.4 Hz, 1H), 2.60 (ddd, J = 1.3, 3.9, 15.0 Hz, 1H), 2.86 (dd, J = 14.9, 19.6 Hz, 1H), 3.08 (dd, J = 3.3, 15.0 Hz, 1H), 3.62 (t, J = 6.1 Hz, 2H), 3.64 – 3.72 (m, 1H), 4.24 (t, J = 6.1 Hz, 2H), 5.20 (s, 1H), 6.37 – 6.47 (m, 2H), 7.07 – 7.15 (m, 2H), 7.2 – 7.24 (m, 1H), 7.39 (s, 1H), 7.49 – 7.56 (m, 1H); m/z: ES+ $[M+H]^+$ = 495.2.

Ethyl 2-(2-(4-(2-(3,5-difluoro-4-((1*R*,3*R*)-2-(2-fluoro-2-methylpropyl)-3-methyl-2,3,4,9-tetrahydro-1*H*-pyrido[3,4-*b*]indol-1-yl)phenoxy)ethyl)piperazin-1-yl)ethoxy)acetate (6d)

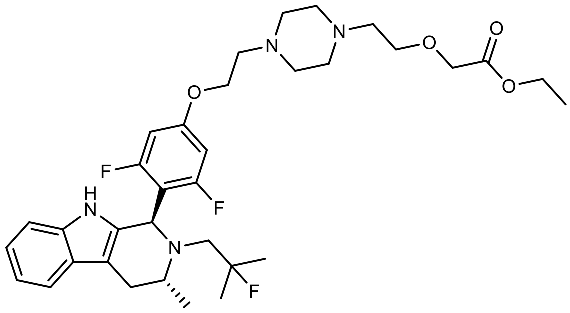

(1*R*,3*R*)-1-[4-(2-Bromoethoxy)-2,6-difluoro-phenyl]-2-(2-fluoro-2-methyl-propyl)-3-methyl-1,3,4,9-tetrahydropyrido[3,4-b]indole (0.24 g, 0.48 mmol) was dissolved in acetonitrile (30 mL). To the stirred solution was added ethyl 2-(2-(piperazin-1-yl)ethoxy)acetate hydrochloride (0.15 g, 0.58 mmol) and potassium carbonate (0.536 g, 3.88 mmol) at room temperature. The mixture was stirred at heated to 75 °C and stirred at that temperature overnight. The reaction mixture was cooled to room temperature and the solvent was removed under reduced pressure. To the residue was added DCM (30 mL) and water (30 mL). After partition, the organic layer was washed with brine (10 mL) and concentrated under reduced pressure. The resulting residue was purified by flash silica chromatography, elution gradient 0 to 10% MeOH in DCM. Product fractions were concentrated under reduced pressure to afford the title compound (0.280 g, 92 %); m/z: ES+ $[M+H]^+$ = 631.5.

(2S,4R)-1-((S)-2-(2-(2-(4-(2-(3,5-Difluoro-4-((1R,3R)-2-(2-fluoro-2-methylpropyl)-3-methyl-2,3,4,9-tetrahydro-1H-pyrido[3,4-b]indol-1-yl)phenoxy)ethyl)piperazin-1-yl)ethoxy)acetamido)-3,3-dimethylbutanoyl)-4-hydroxy-N-(4-(4-methylthiazol-5-yl)benzyl)pyrrolidine-2 (6)

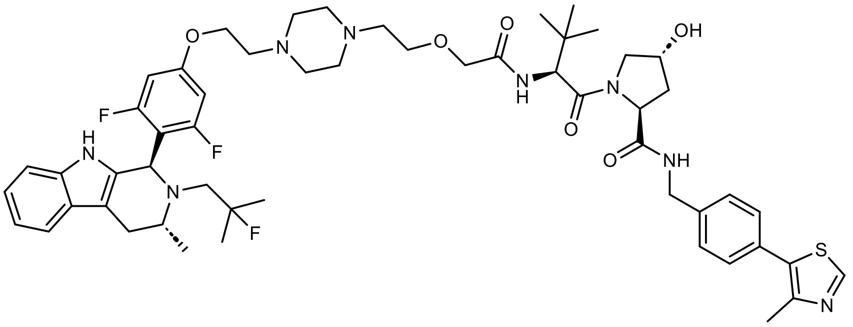

Lithium hydroxide (106 mg, 4.44 mmol) was added to ethyl 2-(2-(4-(2-(3,5-difluoro-4-((1R,3R)-2-(2-fluoro-2-methylpropyl)-3-methyl-2,3,4,9-tetrahydro-1H-pyrido[3,4-b]indol-1-yl)phenoxy)ethyl)piperazin-1-yl)ethoxy)acetate (280 mg, 0.44 mmol) in methanol (3 mL), THF (3 mL), and water (1 mL) at 20°C under air. The resulting solution was stirred at 50 °C for 2 hours. The mixture was evaporated to dryness and 80 mg of the residue was added to a stirred solution of (2S,4R)-1-((S)-2-amino-3,3-dimethylbutanoyl)-4-hydroxy-N-(4-(4-methylthiazol-5-yl)benzyl)pyrrolidine-2-carboxamide hydrochloride (68 mg, 0.15 mmol) and triethylamine (0.056 mL, 0.40 mmol) in DMF (2 mL) at 20°C under nitrogen. HATU (71 mg, 0.19 mmol) was added. The resulting mixture was stirred at 20 °C for 10 minutes. The reaction mixture was purified by preparative HPLC (XBridge Prep C18 OBD column), using an elution gradient of 50 to 75% MeCN (in water containing 0.2% NH4OH). Fractions containing the product were concentrated under reduced pressure to afford (2S,4R)-1-((S)-2-(2-(2-(4-(2-(3,5-difluoro-4-((1R,3R)-2-(2-fluoro-2-methylpropyl)-3-methyl-2,3,4,9-tetrahydro-1H-pyrido[3,4-b]indol-1-yl)phenoxy)ethyl)piperazin-1-yl)ethoxy)acetamido)-3,3-dimethylbutanoyl)-4-hydroxy-N-(4-(4-methylthiazol-5-yl)benzyl)pyrrolidine-2-carboxamide (65 mg, 48%) as a white solid; ^{1}H NMR (500 MHz, $CDCl_3$, 30°C) 0.95 (s, 9H), 1.05 (d, J = 6.6 Hz, 3H), 1.14 (d, J = 21.4 Hz, 3H), 1.20 (d, J = 21.5 Hz, 3H), 1.91 (ddd, J = 12.9, 8.7, 4.5 Hz, 1H), 2.03 - 2.10 (m, 1H), 2.28 - 2.49 (m, 12H), 2.50 (m, 2H, overlapped with DMSO), 2.57 (br d, J = 4.4 Hz, 1H), 2.62 (t, J = 5.8 Hz, 2H), 2.79 - 2.92 (m, 2H), 3.47 - 3.55 (m, 1H), 3.57 - 3.63 (m, 3H), 3.68 (dd, J = 10.5, 4.0 Hz, 1H), 3.91 - 4.00 (m, 2H), 4.04 (t, J = 5.9 Hz, 2H), 4.25 (dd, J = 15.9, 5.6 Hz, 1H), 4.33 - 4.47 (m, 3H), 4.56 (d, J = 9.6 Hz, 1H), 5.08 - 5.18 (m, 2H), 6.61 - 6.67 (m, J = 11.1 Hz, 2H), 6.94 (td, J = 7.2, 1.2 Hz, 1H), 6.99 (td, J = 7.3, 1.1 Hz, 1H), 7.18 (d, J = 7.9 Hz, 1H), 7.34 - 7.42 (m, 6H), 8.58 (t, J = 6.0 Hz, 1H), 8.97 (s, 1H), 10.51 (s, 1H); ^{13}C NMR (126 MHz, $CDCl_3$, 30°C) δ 13.33, 16.39, 24.98, 25.33, 26.66, 27.09, 36.25, 38.37, 42.16, 50.98, 51.07 - 51.13, 53.51, 53.62, 56.11, 56.31 - 56.71, 56.81, 57.04, 57.65, 59.24, 66.80, 69.24, 69.35, 70.09, 97.44, 99.24, 106.84, 109.67, 111.37, 117.91, 118.57, 120.76, 127.44, 127.94, 129.16, 130.17, 131.61, 133.13, 136.71, 139.91,

148.21, 151.88, 160.00, 162.69, 168.96, 169.61, 172.22. *m/z*: ES+ $[M+H]^+$ 1016.0; LCMS purity = 98%; ESI-HRMS calculated for $C_{54}H_{69}F_3N_8O_6S$ $[M+H]^+$ = 975.4666, measured 975.4673.

tert-Butyl ((S)-1-((2S,4S)-4-hydroxy-2-((4-(4-methylthiazol-5-yl)benzyl)carbamoyl)pyrrolidin-1-yl)-3,3-dimethyl-1-oxobutan-2-yl)carbamate (7a)

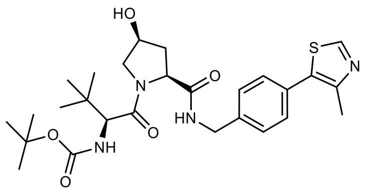

DIAD (0.40 mL, 2.03 mmol) was added to *tert*-butyl ((*S*)-1-((2*S*,4*R*)-4-hydroxy-2-((4-(4-methylthiazol-5-yl)benzyl)carbamoyl)pyrrolidin-1-yl)-3,3-dimethyl-1-oxobutan-2-yl)carbamate[16] (570 mg, 1.07 mmol), 3-nitrobenzoic acid (250 mg, 1.50 mmol) and triphenylphosphine (570 mg, 2.17 mmol) in 1,4-dioxane (10 mL) at 10°C under air. The resulting solution was stirred at RT for 20 hours. The reaction was incomplete and further triphenylphosphine (570 mg, 2.17 mmol) and DIAD (0.40 mL, 2.03 mmol) were added and the solution was stirred at RT for a further 1 hour. The reaction mixture was diluted with EtOAc (50 mL), and washed sequentially with water (10 mL), sat. $NaHCO_3$ (2 x 10 mL), and sat. brine (5 mL). The organic layer was dried with $MgSO_4$, filtered and evaporated to afford crude product. The crude product was purified by flash silica chromatography, elution gradient 30 to 90% EtOAc in heptane. Fractions containing the product were evaporated to dryness to afford crude (3*S*,5*S*)-1-((*S*)-2-((*tert*-butoxycarbonyl)amino)-3,3-dimethylbutanoyl)-5-((4-(4-methylthiazol-5-yl)benzyl)carbamoyl)pyrrolidin-3-yl 3-nitrobenzoate; *m/z*: ES+ $[M+H]^+$ 680.3.
A solution of 2M aq. sodium hydroxide (1 mL, 2.00 mmol) was added to ester (1.83 g, 0.94 mmol) in EtOH (10 mL) at RT under air. The resulting solution was stirred at RT for 1 hour. The reaction mixture was evaporated to dryness and redissolved in EtOAc (25 mL), and washed sequentially with water (5 mL), sat. aq. $NaHCO_3$ (2 x 5 mL), and sat. brine (5 mL). The organic layer was dried with $MgSO_4$, filtered and evaporated to afford crude product. The crude product was purified by flash silica chromatography, elution gradient 50 to 100% EtOAc in heptane. Pure fractions were evaporated to dryness to afford the title compound (436 mg, 87%) as a white solid; ^1H NMR (400 MHz, $CDCl_3$, 30°C) 0.89 (d, *J* = 7.1 Hz, 9H), 1.41 (s, 10H), 2.19 (ddd, *J* = 14.0, 9.1, 5.0 Hz, 1H), 2.39 (d, *J* = 13.9 Hz, 1H), 2.52 (s, 3H), 3.78 (d, *J* = 10.8 Hz, 1H), 3.83 – 3.97 (m, 1H), 4.18 (d, *J* = 9.6 Hz, 1H), 4.30 (dd, *J* = 15.0, 4.6 Hz, 1H), 4.48 (dt, *J* = 9.3, 4.3 Hz, 1H), 4.64 (dd, *J* = 14.9, 7.1 Hz, 1H), 4.77 (d, *J* = 8.9 Hz, 1H), 5.09 (d, *J* = 9.1 Hz, 1H), 5.47 (d, *J* = 9.7 Hz, 1H), 7.36 (q, *J* = 8.3 Hz, 4H), 8.68 (s, 1H); *m/z*: ES+ $[M+H]^+$ 531.2.

(2*S*,4*S*)-1-((*S*)-2-Amino-3,3-dimethylbutanoyl)-4-hydroxy-*N*-(4-(4-methylthiazol-5-yl)benzyl)pyrrolidine-2-carboxamide. HCl (7b)

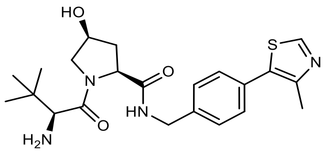

A solution of 6M hydrogen chloride in IPA (3 mL, 18.00 mmol) was added to *tert*-butyl ((*S*)-1-((2*S*,4*S*)-4-hydroxy-2-((4-(4-methylthiazol-5-yl)benzyl)carbamoyl)pyrrolidin-1-yl)-3,3-dimethyl-1-oxobutan-2-yl)carbamate (0.43 g, 0.81 mmol) in IPA (1 mL) at RT under air. The resulting solution was stirred at RT for 2 hours. The reaction mixture was diluted with Et_2O (10 mL) and stirred for 30 minutes. The precipitate was collected by filtration, washed with Et_2O (5 mL) and dried under vacuum to afford the title compound (0.33 g, 86%) as a white solid; ^1H NMR (400 MHz, DMSO-d6, 30°C) 0.96 – 1.06 (m, 9H), 1.74 (dt, *J* = 12.6, 7.2 Hz, 1H), 2.39 (ddd, *J* = 12.6, 8.4, 6.1 Hz, 1H), 2.45 (s, 3H), 3.31 (dd, *J* = 10.1, 6.5 Hz, 1H), 3.91 (d, *J* = 5.4 Hz, 1H), 4.01 (dd, *J* = 10.2, 6.0 Hz, 1H), 4.14 – 4.5 (m, 5H), 7.40 (s, 4H), 8.01 – 8.26 (m, 3H), 8.74 (t, *J* = 6.0 Hz, 1H), 9.02 (s, 1H); *m/z*: ES+ $[M+H]^+$ 431.2.

(2*S*,4*S*)-1-((*S*)-2-(2-(3-(3-(3,5-Difluoro-4-((1*R*,3*R*)-2-(2-fluoro-2-methylpropyl)-3-methyl-2,3,4,9-tetrahydro-1*H*-pyrido[3,4-*b*]indol-1-yl)phenoxy)propoxy)propoxy)acetamido)-3,3-dimethylbutanoyl)-4-hydroxy-*N*-(4-(4-methylthiazol-5-yl)benzyl)pyrrolidine-2-carboxamide (7)

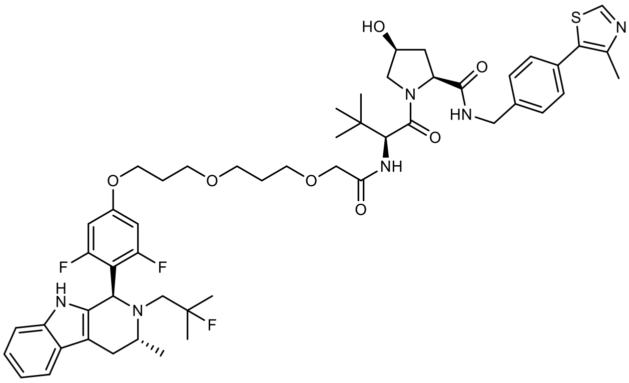

HATU (135 mg, 0.36 mmol) was added to 2-(3-(3-(3,5-difluoro-4-((1*R*,3*R*)-2-(2-fluoro-2-methylpropyl)-3-methyl-2,3,4,9-tetrahydro-1*H*-pyrido[3,4-b]indol-1-yl)phenoxy)propoxy)propoxy)acetic acid (**5f**) (100 mg, 0.18 mmol), (2*S*,4*S*)-1-((*S*)-2-amino-3,3-dimethylbutanoyl)-4-hydroxy-*N*-(4-(4-methylthiazol-5-yl)benzyl)pyrrolidine-2-carboxamide, HCl (100 mg, 0.21 mmol) and DIPEA (125 µl, 0.72 mmol) in DCM (3 mL) at RT under air. The resulting solution was stirred at RT for 30 minutes. The reaction mixture was diluted with EtOAc (50 mL), and washed

sequentially with water (5 mL), sat. $NaHCO_3$ (2 x 5 mL), and sat. brine (2 mL). The organic layer was dried with $MgSO_4$, filtered and evaporated to afford crude product. The crude product was purified by flash silica chromatography, elution gradient 3 to 9% MeOH in DCM. Product containing fractions were evaporated to dryness. The residue was repurified by preparative HPLC (Waters XSelect CSH C18 column, 5µ silica, 30 mm diameter, 100 mm length), using decreasingly polar mixtures of water (containing 0.1% formic acid) and MeCN as eluents. Fractions containing the desired compound were evaporated to dryness to afford the title compound (71 mg, 41%) as a beige solid; ^{1}H NMR (400 MHz, $CDCl_3$, 30°C) 0.93 (s, 9H), 1.09 (d, *J* = 6.5 Hz, 3H), 1.21 (dd, *J* = 21.5, 14.1 Hz, 6H), 1.84 (td, *J* = 5.9, 1.7 Hz, 2H), 1.96 – 2.03 (m, 2H), 2.18 (ddd, *J* = 14.0, 9.1, 4.9 Hz, 1H), 2.41 (dd, *J* = 25.8, 14.5 Hz, 2H), 2.51 (s, 3H), 2.60 (dd, *J* = 14.8, 3.9 Hz, 1H), 2.74 – 2.87 (m, 1H), 3.05 (dd, *J* = 15.2, 3.7 Hz, 1H), 3.37 – 3.5 (m, 3H), 3.52 (td, *J* = 5.9, 1.8 Hz, 2H), 3.57 (t, *J* = 5.8 Hz, 2H), 3.64 (q, *J* = 4.9 Hz, 1H), 3.72 – 3.86 (m, 2H), 3.9 – 4.05 (m, 3H), 4.31 (dd, *J* = 14.9, 5.1 Hz, 1H), 4.48 (s, 1H), 4.55 (d, *J* = 9.4 Hz, 1H), 4.64 (dd, *J* = 14.9, 7.0 Hz, 1H), 4.77 (d, *J* = 8.8 Hz, 1H), 5.18 (s, 1H), 5.44 (s, 1H), 6.26 – 6.4 (m, 2H), 7.01 – 7.11 (m, 3H), 7.16 – 7.25 (m, 1H), 7.3 – 7.42 (m, 4H), 7.43 – 7.56 (m, 2H), 8.67 (s, 1H), 8.80 (s, 1H); ^{13}C NMR (126 MHz, $CDCl_3$, 17°C) δ 13.0, 16.1, 24.8, 25.2, 26.3, 27.0, 29.1, 29.6, 34.9, 35.3, 43.5, 50.9, 56.1, 56.5, 58.7, 60.0, 64.7, 66.1, 66.9, 68.2, 69.7, 71.1, 97.4, 98.2, 108.0, 109.9, 110.8, 118.0, 118.8, 120.9, 127.5, 128.3, 129.7, 131.3, 131.5, 132.5, 136.4, 137.3, 148.5, 150.5, 159.9, 162.7, 169.7, 171.7, 172.6;*m/z*: ES+ $[M+H]^+$ 975.5; LCMS purity = 100%; ESI-HRMS calculated for $C_{52}H_{65}F_3N_6O_7S$ $[M+H]^+$ = 975.4660, measured 975.4695.

Benzyl propiolate (8a)

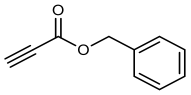

Propiolic acid (1.00 mL, 16.3 mmol) was added dropwise to cesium carbonate (13.26 g, 40.69 mmol) in DMF (10 mL) cooled to 0°C over a period of 10 minutes under air. The resulting solution was stirred at 0 °C for 20 minutes. Benzyl bromide (1.61 mL, 13.6 mmol) was added dropwise, the reaction mixture warmed to RT and stirred for 1.5 hours. The reaction mixture was diluted with Et_2O (50 mL), and washed sequentially with sat. brine (5 x 20 mL). The organic layer was dried with $MgSO_4$, filtered and evaporated to afford crude product. The crude product was purified by flash silica chromatography, elution gradient 0 to 10% EtOAc in heptane. Pure fractions were evaporated to dryness to afford the title compound (1.12 g, 52%) as a colourless oil; ^{1}H NMR (400 MHz, DMSO-d6, 27°C) 0.77 – 0.94 (m, 0H), 2.00 (s, 0H), 4.61 (s, 1H), 5.23 (s, 2H), 7.25 – 7.48 (m, 5H).

Benzyl (E)-3-(3,5-difluoro-4-((1*R*,3*R*)-2-(2-fluoro-2-methylpropyl)-3-methyl-2,3,4,9-tetrahydro-1*H*-pyrido[3,4-*b*]indol-1-yl)phenoxy)acrylate (8b)

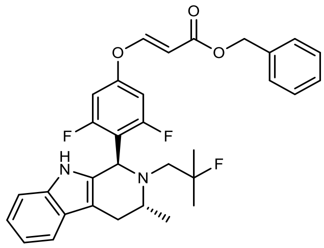

4-Methylmorpholine (6 µL, 0.05 mmol) was added to benzyl propiolate (90 mg, 0.56 mmol) and 3,5-difluoro-4-((1*R*,3*R*)-2-(2-fluoro-2-methylpropyl)-3-methyl-2,3,4,9-tetrahydro-1*H*-pyrido[3,4-*b*]indol-1-yl)phenol (**3a**) (200 mg, 0.51 mmol) in DCM (2 mL) cooled to 0°C under nitrogen. The resulting solution was stirred at RT for 2 hours. The reaction mixture was diluted with EtOAc (10 mL), and washed sequentially with 5% aq. AcOH (2 x 2mL) and sat. brine (2 mL). The organic layer was dried with MgSO4, filtered and evaporated to afford crude product. The crude product was purified by flash silica chromatography, elution gradient 0 to 40% EtOAc in heptane. Pure fractions were evaporated to dryness to afford the title compound (174 mg, 62%) as a white solid; ^{1}H NMR (500 MHz, DMSO-d6, 27°C) 1.05 (d, *J* = 6.5 Hz, 3H), 1.14 (d, *J* = 21.4 Hz, 3H), 1.21 (d, *J* = 21.5 Hz, 3H), 2.35 (dd, *J* = 23.3, 15.0 Hz, 1H), 2.57 (dd, *J* = 15.0, 4.6 Hz, 1H), 2.8 – 2.96 (m, 2H), 3.51 (q, *J* = 5.2 Hz, 1H), 5.18 (s, 3H), 5.77 (d, *J* = 12.0 Hz, 1H), 6.95 (t, *J* = 7.4 Hz, 1H), 7.01 (t, *J* = 7.4 Hz, 1H), 7.09 (d, *J* = 10.0 Hz, 2H), 7.19 (d, *J* = 7.9 Hz, 1H), 7.3 – 7.45 (m, 6H), 7.95 (d, *J* = 12.0 Hz, 1H), 10.56 (s, 1H); *m/z*: ES+ $[M+H]^+$ 549.2.

3-(3,5-Difluoro-4-((1*R*,3*R*)-2-(2-fluoro-2-methylpropyl)-3-methyl-2,3,4,9-tetrahydro-1*H*-pyrido[3,4-*b*]indol-1-yl)phenoxy)propanoic acid (8)

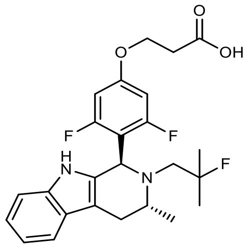

Benzyl (E)-3-(3,5-difluoro-4-((1*R*,3*R*)-2-(2-fluoro-2-methylpropyl)-3-methyl-2,3,4,9-tetrahydro-1*H*-pyrido[3,4-*b*]indol-1-yl)phenoxy)acrylate (170 mg, 0.31 mmol) and 10% palladium on carbon (33 mg, 0.03 mmol) in EtOAc (3 mL) was stirred under an atmosphere of hydrogen at 1 atm and RT for 3 hours. The reaction mixture was filtered through Celite® and the filtrate evaporated to dryness. The crude product was purified by flash silica chromatography, elution gradient 0 to 8% MeOH in DCM.

Product containing fractions were evaporated to dryness to afford impure product which was repurified by flash silica chromatography, elution gradient 0 to 60% EtOAc in heptane. Pure fractions were evaporated to dryness to afford the title compound (22 mg, 15%) as a white solid; ^{1}H NMR (500 MHz, DMSO, 27°C) 1.05 (d, *J* = 6.5 Hz, 3H), 1.17 (dd, *J* = 29.4, 21.5 Hz, 6H), 2.35 (dd, *J* = 24.1, 14.9 Hz, 1H), 2.56 (dd, *J* = 15.0, 4.5 Hz, 1H), 2.67 (t, *J* = 5.9 Hz, 2H), 2.78 – 2.93 (m, 2H), 3.52 (q, *J* = 5.1 Hz, 1H), 4.18 (t, *J* = 5.9 Hz, 2H), 5.13 (s, 1H), 6.67 (d, *J* = 11.1 Hz, 2H), 6.94 (t, *J* = 7.4 Hz, 1H), 6.99 (t, *J* = 7.3 Hz, 1H), 7.18 (d, *J* = 7.9 Hz, 1H), 7.39 (d, *J* = 7.7 Hz, 1H), 10.51 (s, 1H), 12.46 (br. s, 1H); ^{13}C NMR (126 MHz, DMSO-d6, 27°C) 13.1, 22.6, 24.9, 25.2, 27.1, 31.7, 34.5, 51.0, 56.6, 65.1, 97.5, 99.2, 106.8, 109.8, 111.4, 117.9, 118.6, 120.8, 127.4, 133.1, 136.7, 159.9, 162.7, 172.6; *m/z*: ES+ $[M+H]^+$ 461.3; LCMS purity = 100%; ESI-HRMS calculated for $C_{25}H_{27}F_3N_2O_3$ $[M+H]^+$ = 461.2052, measured 461.2044.

2-(2-(4-(Benzyloxy)butoxy)ethoxy)tetrahydro-2H-pyran (9a)

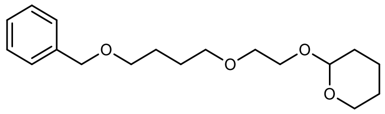

Tetrabutylammonium hydrogen sulfate (1.158 g, 3.41 mmol) was added in one portion to 2-(2-bromoethoxy)tetrahydro-2H-pyran (4.12 mL, 27.3 mmol) and 4-(benzyloxy)butan-1-ol (4.0 mL, 22.8 mmol) in sodium hydroxide solution 50% (7.0 mL) at 20 °C. The resulting mixture was stirred at 70 °C for 18 hours. The cooled reaction mixture was diluted with water (20 mL) and extracted with EtOAc (3 x 25 mL). The combined organic extracts were washed with water (20 mL), saturated brine solution (20 mL), dried ($MgSO_4$), filtered and evaporated to afford crude product as a yellow oil. The crude product was purified by flash silica chromatography, elution gradient 0 to 30% EtOAc in heptane. Pure fractions were evaporated to dryness to afford the title compound (4.00 g, 57.0 %) as a yellow oil. ^{1}H NMR (400 MHz, $CDCl_3$, 30°C) 1.46 – 1.76 (9H, m), 1.83 (1H, ddt), 3.49 (5H, ddt), 3.56 – 3.65 (3H, m), 3.79 – 3.93 (2H, m), 4.50 (2H, s), 4.6 – 4.66 (1H, m), 7.28 (1H, dd), 7.3 – 7.37 (4H, m).

2-(4-(Benzyloxy)butoxy)ethan-1-ol (9b)

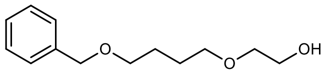

2-(2-(4-(Benzyloxy)butoxy)ethoxy)tetrahydro-2H-pyran (4.0 g, 13 mmol) was dissolved in MeOH (35 mL) and 1M aq. HCl (15 mL) was added. The resulting mixture was stirred at 20 °C for 1 hour. The reaction mixture was diluted with water (50 mL), and extracted with EtOAc (3 x 75 mL). The combined

organics were washed with saturated brine (50 mL). The organic layer was dried with $MgSO_4$, filtered and evaporated to afford the title compound (3.34 g) as a colourless oil that was used directly in the next step without purification. ^{1}H NMR (400 MHz, $CDCl_3$, 30°C) 1.67 – 1.72 (4H, m), 3.46 – 3.55 (6H, m), 3.68 – 3.74 (2H, m), 4.50 (2H, s), 7.26 – 7.31 (1H, m), 7.31 – 7.37 (4H, m); *m/z*: ES+ $[M+H]^+$ 225.3

Ethyl 2-(2-(4-(benzyloxy)butoxy)ethoxy)acetate (9c)

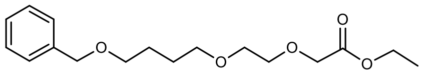

Ethyl 2-diazoacetate (1.9 mL, 15.6 mmol) in DCM (15 mL) was added slowly to 2-(4-(benzyloxy)butoxy)ethan-1-ol (2.91 g, 13.0 mmol) and rhodium acetate dimer (0.057 g, 0.13 mmol) in DCM (40 mL) at 20 °C over a period of 1 hour under nitrogen. The resulting solution was stirred at 20 °C for 3 hours. The mixture was diluted with DCM (50 mL) and washed with water (3 x 50 mL). The organic layer was collected and dried using a phase separating cartridge then evaporated to dryness. The crude product was purified by flash silica chromatography, elution gradient 0 to 25% EtOAc in heptane. Pure fractions were evaporated to dryness to afford the title compound (2.43 g, 60 %) as a colourless liquid. ^{1}H NMR (400 MHz, $CDCl_3$, 30°C) 1.28 (3H, t), 1.64 – 1.72 (4H, m), 3.45 – 3.52 (4H, m), 3.59 – 3.64 (2H, m), 3.69 – 3.73 (2H, m), 4.14 (2H, s), 4.21 (2H, q), 4.50 (2H, s), 7.26 – 7.31 (1H, m), 7.31 – 7.36 (4H, m).

Ethyl 2-(2-(4-hydroxybutoxy)ethoxy)acetate (9d)

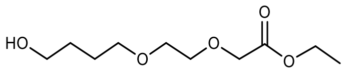

Ethyl 2-(2-(4-(benzyloxy)butoxy)ethoxy)acetate (2.4 g, 7.73 mmol) and 10% palladium on carbon (0.082 g, 0.77 mmol) in ethanol (15 mL) were stirred under hydrogen at 2 bar for 2 hours at room temperature. The reaction mixture was filtered, washing with more EtOH then the solvent was evaporated to afford the title compound (1.75 g, 100 %) as a colourless oil which was used in the next step without further purification. ^{1}H NMR (400 MHz, $CDCl_3$, 30°C) 1.29 (3H, t), 1.68 (4H, ddtd), 2.15 (1H, s), 3.53 (2H, t), 3.62 – 3.68 (4H, m), 3.71 – 3.74 (2H, m), 4.14 (2H, s), 4.22 (2H, q); *m/z*: ES+ $[M+H]^+$ 221.2

Ethyl 2-(2-(4-(3,5-difluoro-4-((1R,3R)-2-(2-fluoro-2-methylpropyl)-3-methyl-2,3,4,9-tetrahydro-1H-pyrido[3,4-b]indol-1-yl)phenoxy)butoxy)ethoxy)acetate (9e)

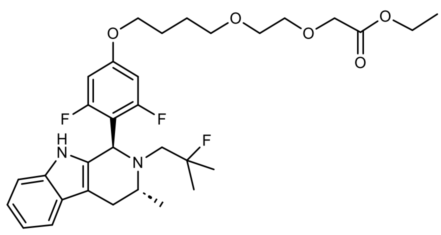

DIAD (0.30 mL, 1.54 mmol) was added dropwise to a stirred solution of 3,5-difluoro-4-((1R,3R)-2-(2-fluoro-2-methylpropyl)-3-methyl-2,3,4,9-tetrahydro-1H-pyrido[3,4-b]indol-1-yl)phenol (300 mg, 0.77 mmol), ethyl 2-(2-(4-hydroxybutoxy)ethoxy)acetate (340 mg, 1.54 mmol) and triphenylphosphine (405 mg, 1.54 mmol) in DCM (10 mL) at 20 °C. The resulting mixture was stirred for 1 hour. The reaction mixture was loaded directly onto a 24 g silica column and was purified by flash silica chromatography, elution gradient 0 to 50% EtOAc in heptane. Pure fractions were evaporated to dryness to afford the title compound (530 mg) as a yellow gum that was used without further purification. ^{1}H NMR (400 MHz, $CDCl_3$, 30°C) 1.10 (3H, d), 1.20 (6H, dd), 1.27 (3H, t), 1.7 – 1.79 (2H, m), 1.81 – 1.92 (2H, m), 2.39 (1H, dd), 2.60 (1H, dd), 2.86 (1H, dd), 3.09 (1H, dd), 3.53 (2H, t), 3.61 – 3.65 (2H, m), 3.66 – 3.7 (1H, m), 3.7 – 3.74 (2H, m), 3.95 (2H, t), 4.13 (2H, s), 4.20 (2H, q), 5.18 (1H, s), 6.35 – 6.43 (2H, m), 7.05 – 7.14 (2H, m), 7.19 – 7.24 (1H, m), 7.47 (1H, s), 7.49 – 7.53 (1H, m); *m/z*: ES+ $[M+H]^+$ 591.5

2-(2-(4-(3,5-Difluoro-4-((1R,3R)-2-(2-fluoro-2-methylpropyl)-3-methyl-2,3,4,9-tetrahydro-1H-pyrido[3,4-b]indol-1-yl)phenoxy)butoxy)ethoxy)acetic acid (9f)

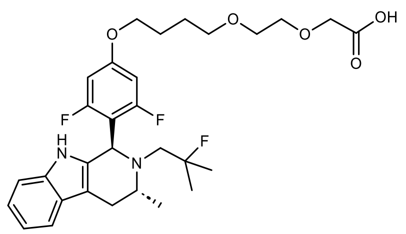

Lithium hydroxide hydrate (64 mg, 1.5 mmol) was added in one portion to ethyl 2-(2-(4-(3,5-difluoro-4-((1R,3R)-2-(2-fluoro-2-methylpropyl)-3-methyl-2,3,4,9-tetrahydro-1H-pyrido[3,4-b]indol-1-yl)phenoxy)butoxy)ethoxy)acetate (450 mg, 0.76 mmol) in THF (3 mL) and water (1 mL). The resulting solution was stirred at 20 °C for 30 minutes. The reaction mixture was diluted with water (10 mL), acidified with 2M HCl and extracted into EtOAc (50 mL). The organic layer was washed with brine (15 mL) and evaporated to afford the title compound (420 mg, 98 %) as a yellow gum which was used in the next step without further purification; *m/z*: ES+ $[M+H]^+$ 563.5

(2S,4R)-1-((S)-2-(2-(2-(4-(3,5-Difluoro-4-((1R,3R)-2-(2-fluoro-2-methylpropyl)-3-methyl-2,3,4,9-tetrahydro-1H-pyrido[3,4-b]indol-1-yl)phenoxy)butoxy)ethoxy)acetamido)-3,3-dimethylbutanoyl)-4-hydroxy-N-(4-(4-methylthiazol-5-yl)benzyl)pyrrolidine-2-carboxamide (9)

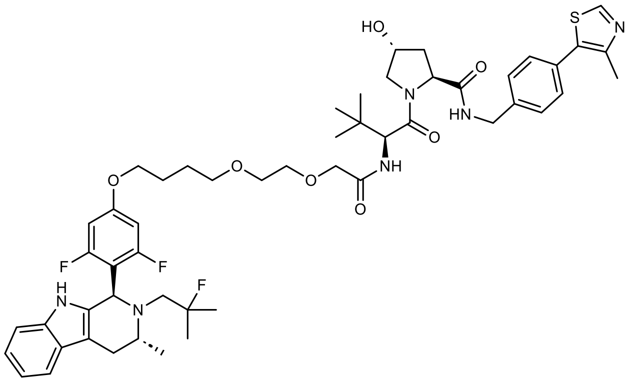

HATU (213 mg, 0.56 mmol) was added portionwise to 2-(2-(4-(3,5-difluoro-4-((1R,3R)-2-(2-fluoro-2-methylpropyl)-3-methyl-2,3,4,9-tetrahydro-1H-pyrido[3,4-b]indol-1-yl)phenoxy)butoxy)ethoxy)acetic acid (210 mg, 0.37 mmol), (2S,4R)-1-((S)-2-amino-3,3-dimethylbutanoyl)-4-hydroxy-N-(4-(4-methylthiazol-5-yl)benzyl)pyrrolidine-2-carboxamide, 2HCl (188 mg, 0.37 mmol) and triethylamine (0.208 ml, 1.49 mmol) in DMF (4 ml) at 20°C under nitrogen. The resulting mixture was stirred at 20 °C for 30 minutes. The reaction mixture was diluted with EtOAc (50 mL), and washed sequentially with water (20 mL) and saturated brine (20 mL). The organic layer was dried with $MgSO_4$, filtered and evaporated to afford crude product. The crude product was purified by preparative HPLC (Waters CSH C18 OBD column, 30 x 100 mm id, 5 micron particle size), using decreasingly polar mixtures of water (containing 0.1% NH3) and MeCN as eluents to afford the title compound (146 mg, 40 %) as a white solid; ^{1}H NMR (400 MHz, CDCl3, 30°C) 0.94 (9H, s), 1.10 (3H, d), 1.20 (6H, dd), 1.73 (2H, dt), 1.78 – 1.89 (2H, m), 2 – 2.13 (1H, m), 2.40 (1H, dd), 2.46 (3H, s), 2.52 – 2.66 (2H, m), 2.73 (1H, d), 2.85 (1H, dd), 3.08 (1H, dd), 3.53 (2H, t), 3.56 – 3.72 (6H, m), 3.83 – 4.04 (4H, m), 4.08 (1H, d), 4.29 (1H, dd), 4.48 (1H, d), 4.51 (1H, s), 4.58 (1H, dd), 4.71 (1H, t), 5.19 (1H, s), 6.25 – 6.38 (2H, m), 7.01 – 7.14 (2H, m), 7.18 – 7.25 (1H, m), 7.28 (1H, s), 7.3 – 7.39 (5H, m), 7.48 – 7.54 (1H, m), 8.37 (1H, s), 8.61 (1H, s); ^{13}C NMR (126 MHz, DMSO-d6, 27°C) 12.5, 15.6, 24.2, 24.5, 25.0, 25.3, 25.9, 26.3, 35.4, 37.6, 41.4, 50.2 - 50.4 (2C), 55.4, 55.7, 56.2, 58.4, 67.8, 68.6, 68.9, 69.3, 69.6, 70.2, 96.7, 98.3, 106.0, 108.8, 110.6, 117.1, 117.8, 120.0, 126.7, 127.2, 128.4, 129.4 130.8, 132.4, 135.9, 139.1, 147.4, 151.1, 159.4, 161.9, 168.2, 168.8, 171.4; *m/z*: ES- $[M-H]^-$ 973.5; LCMS purity = 100%; ESI-HRMS calculated for $C_{52}H_{66}F_3N_6O_7S$ $[M+H]^+$: 975.4660, observed 975.4633.

2-(3-(2-(Benzyloxy)ethoxy)propoxy)tetrahydro-2H-pyran (10a)

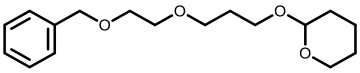

Tetrabutylammonium hydrogen sulfate (0.951 g, 2.80 mmol) was added in one portion to 2-(benzyloxy)ethan-1-ol (2.66 mL, 18.7 mmol) and 2-(3-bromopropoxy)tetrahydro-2H-pyran (5.0 g, 22.4 mmol) in 50% aq. NaOH solution (12.3 mL) at 20 °C. The resulting mixture was stirred at 70 °C for 4 hours. The cooled reaction mixture was diluted with water (20 mL) and extracted with EtOAc (3 x 25 mL). The combined organic extracts were washed with water (20 mL), saturated brine solution (20 mL), dried ($MgSO_4$), filtered and evaporated to afford crude product. The crude product was purified by flash column chromatography, elution gradient 0 to 10% EtOAc in heptane to afford the title compound (4.31 g, 78%) as a colourless oil; ^{1}H NMR (400 MHz, $CDCl_3$) 1.47 - 1.6 (4H, m), 1.65 - 1.74 (1H, m), 1.80 (1H, m), 1.89 (2H, p), 3.45 - 3.53 (2H, m), 3.54 - 3.65 (6H, m), 3.83 (2H, m), 4.57 (3H, s), 7.23 - 7.37 (5H, m).

3-(2-(Benzyloxy)ethoxy)propan-1-ol (10b)

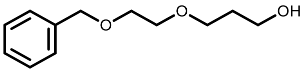

2-(3-(2-(Benzyloxy)ethoxy)propoxy)tetrahydro-2H-pyran (4.3 g, 14.6 mmol) was dissolved in MeOH (39.0 mL) and 1M aq. HCl (19.5 mL) was added. The resulting mixture was stirred at 20 °C for 3 hours. The reaction mixture was diluted with water (100 mL), and extracted with EtOAc (3 x 100 mL). The combined organics were washed with saturated brine (20 mL). The organic layer was dried with $MgSO_4$, filtered and evaporated to afford the title compound (2.95 g, 96%) as a colourless liquid; ^{1}H NMR (400 MHz, $CDCl_3$) 1.85 (2H, p), 2.38 (1H, s), 3.59 - 3.66 (4H, m), 3.68 (2H, t), 3.78 (2H, s), 4.57 (2H, s), 7.27 - 7.3 (1H, m), 7.34 (4H, d).

Ethyl 2-(3-(2-(benzyloxy)ethoxy)propoxy)acetate (10c)

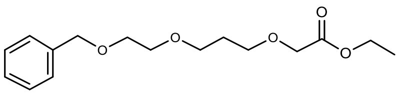

Ethyl 2-diazoacetate (4.30 mL, 35.07 mmol) in DCM (14.39 mL) was added slowly to 3-(2-(benzyloxy)ethoxy)propan-1-ol (2.95 g, 14.0 mmol) and rhodium acetate dimer (0.310 g, 0.70 mmol) in DCM (37 mL) at 20 °C over a period of 1 hour under nitrogen. The resulting solution was stirred at 20 °C for 18 hours. The mixture was diluted with DCM (50 mL) and washed with water (20 mL). The

organic layer was collected and dried using phase separating cartridge then evaporated to dryness. The crude product was purified by flash column chromatography, elution gradient 0 to 10% EtOAc in heptane to afford the title compound (3.47 g, 83%) a a colourless liquid; ^{1}H NMR (400 MHz, $CDCl_3$) 1.29 (3H, m), 1.92 (2H, p), 3.56 - 3.67 (8H, m), 4.05 (2H, s), 4.18 - 4.26 (2H, m), 4.57 (2H, s), 7.27 - 7.3 (1H, m), 7.31 - 7.37 (4H, m).

Ethyl 2-(3-(2-hydroxyethoxy)propoxy)acetate (10d)

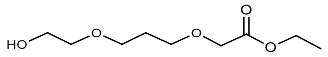

Ethyl 2-(3-(2-(benzyloxy)ethoxy)propoxy)acetate (3.47 g, 11.71 mmol) and 10% palladium on carbon (0.125 g, 1.17 mmol) in EtOH (23 mL) were stirred under an atmosphere of hydrogen (1.5 bar) at RT for 4 hours. The reaction mixture was filtered and evaporated to afford crude product. The crude product was purified by flash column chromatography, elution gradient 0 to 100% EtOAc in heptane to afford the title compound (1.750 g, 73%) as a colourless oil; ^{1}H NMR (400 MHz, $CDCl_3$) 1.28 (3H, t), 1.91 (2H, p), 2.25 (1H, t), 3.53 - 3.59 (2H, m), 3.63 (4H, m), 3.73 (2H, m), 4.07 (2H, s), 4.22 (2H, q).

Ethyl 2-(3-(2-(3,5-difluoro-4-((1*R*,3*R*)-2-(2-fluoro-2-methylpropyl)-3-methyl-2,3,4,9-tetrahydro-1*H*-pyrido[3,4-b]indol-1-yl)phenoxy)ethoxy)propoxy)acetate (10e)

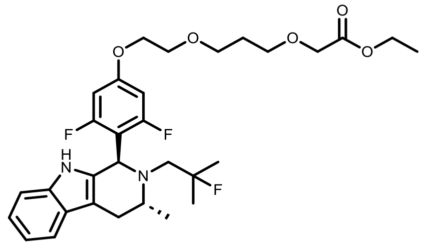

DIAD (0.152 mL, 0.77 mmol) was added dropwise to a stirred mixture of 3,5-difluoro-4-((1*R*,3*R*)-2-(2-fluoro-2-methylpropyl)-3-methyl-2,3,4,9-tetrahydro-1*H*-pyrido[3,4-b]indol-1-yl)phenol (150mg, 0.39 mmol), ethyl 2-(3-(2-hydroxyethoxy)propoxy)acetate (159 mg, 0.77 mmol) and triphenylphosphine (203 mg, 0.77 mmol) in DCM (6.3 mL) at 0 °C. The resulting mixture was stirred at RT for 18 hours. DCM (50 mL) and water (25 mL) were added and the layers were separated. The DCM layer was passed through a phase separating cartridge and concentrated to give the crude product. The crude product was purified by flash column chromatography, elution gradient 0 to 25% EtOAc in heptane to afford the title compound (327 mg, contains solvents) as a pale yellow gum that was used in the next step without further purification; *m/z*: ES+ $[M+H]^+$ 577.4.

2-(3-(2-(3,5-Difluoro-4-((1*R*,3*R*)-2-(2-fluoro-2-methylpropyl)-3-methyl-2,3,4,9-tetrahydro-1*H*-pyrido[3,4-b]indol-1-yl)phenoxy)ethoxy)propoxy)acetic acid (10f)

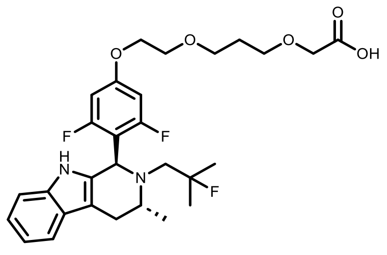

Lithium hydroxide hydrate (32.5 mg, 0.77 mmol) was added in one portion to ethyl 2-(3-(2-(3,5-difluoro-4-((1*R*,3*R*)-2-(2-fluoro-2-methylpropyl)-3-methyl-2,3,4,9-tetrahydro-1*H*-pyrido[3,4-b]indol-1-yl)phenoxy)ethoxy)propoxy)acetate (223mg, 0.39 mmol) in THF (1.5 mL) and water (0.5 mL) at 20 °C. The resulting solution was stirred for 30 minutes. The reaction mixture was diluted with water (10 mL) then was acidified with 2M aq. HCl and extracted into EtOAc (50 mL). The organic layer was washed with brine (15 mL) and evaporated to afford the title compound (212 mg, 100%) as a yellow gum; *m/z*: ES+ $[M+H]^+$ 549.3.

(2*S*,4*R*)-1-((*S*)-2-(2-(3-(2-(3,5-Difluoro-4-((1*R*,3*R*)-2-(2-fluoro-2-methylpropyl)-3-methyl-2,3,4,9-tetrahydro-1*H*-pyrido[3,4-b]indol-1-yl)phenoxy)ethoxy)propoxy)acetamido)-3,3-dimethylbutanoyl)-4-hydroxy-*N*-(4-(4-methylthiazol-5-yl)benzyl)pyrrolidine-2-carboxamide (10)

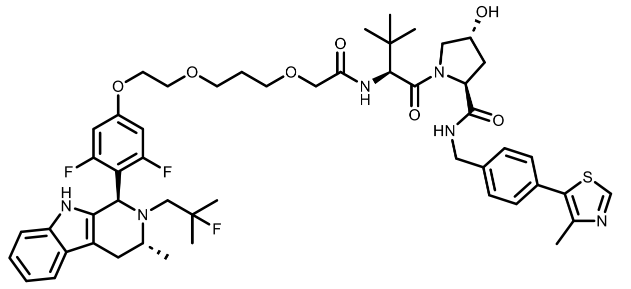

HATU (220 mg, 0.58 mmol) was added in one portion to (2*S*,4*R*)-1-((*S*)-2-amino-3,3-dimethylbutanoyl)-4-hydroxy-*N*-(4-(4-methylthiazol-5-yl)benzyl)pyrrolidine-2-carboxamide, HCl (180 mg, 0.39 mmol), 2-(3-(2-(3,5-difluoro-4-((1*R*,3*R*)-2-(2-fluoro-2-methylpropyl)-3-methyl-2,3,4,9-tetrahydro-1*H*-pyrido[3,4-b]indol-1-yl)phenoxy)ethoxy)propoxy)acetic acid (212mg, 0.39 mmol) and triethylamine (0.215 mL, 1.55 mmol) in DMF (14.200 mL) at 20 °C under nitrogen. The resulting mixture was stirred at 20 °C for 30 minutes. The reaction mixture was diluted with EtOAc (50 mL), and washed sequentially with water (50 mL) and saturated brine (25 mL). The organic layer was

dried with $MgSO_4$, filtered and evaporated to afford crude material. The crude product was purified by preparative HPLC to afford the title compound (151 mg, 41%) as a white solid; ^1H NMR (400 MHz, $CDCl_3$) 0.94 (9H, s), 1.09 (3H, d), 1.21 (6H, dd), 1.87 (2H, p), 2.04 - 2.13 (1H, m), 2.40 (1H, dd), 2.48 (3H, s), 2.51 - 2.65 (2H, m), 2.78 - 2.9 (2H, m), 3.07 (1H, dd), 3.55 (2H, t), 3.58 - 3.69 (5H, m), 3.76 (2H, m), 3.87 (1H, d), 3.96 - 4.11 (3H, m), 4.33 (1H, dd), 4.55 (3H, t), 4.72 (1H, t), 5.19 (1H, s), 6.34 - 6.42 (2H, m), 7.01 - 7.12 (2H, m), 7.17 (1H, d), 7.2 - 7.25 (2H, m), 7.3 - 7.41 (4H, m), 7.50 (1H, m), 8.49 (1H, s), 8.64 (1H, s); ^{13}C NMR (126 MHz, DMSO-d6, 27°C) 12.5, 15.6, 24.2, 24.6, 25.9, 26.4, 29.1, 35.5, 37.6, 41.4, 50.2 - 50.3, 55.4, 55.8, 56.3, 58.5, 67.0, 67.63 - 67.68 (2C), 68.1, 68.6, 69.2, 96.7, 98.4, 106.0, 109.0, 110.6, 117.1, 117.8, 120.0, 126.7, 127.2, 128.4, 129.4, 130.8, 132.3, 136.0, 139.1, 147.4, 151.1, 159.2, 161.9, 168.2, 168.9, 171.4; *m/z*: ES+ $[M+H]^+$; LCMS purity = 100%; 961.6; ESI-HRMS calculated for $C_{51}H_{64}F_3N_6O_7S$ $[M+H]^+$ = 961.4504, measured 961.4467.

cis-2-((2-((Tetrahydro-2H-pyran-2-yl)oxy)ethoxy)methyl)cyclopropyl)methanol (11a)

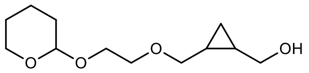

A solid suspension of 60% sodium hydride in mineral oil (0.244 g, 6.11 mmol) was added to (cis-cyclopropane-1,2-diyl)dimethanol (0.52 g, 5.09 mmol) in THF (10 mL) cooled to 0°C under nitrogen. The resulting suspension was stirred at 0 °C for 45 minutes. 2-(2-bromoethoxy)tetrahydro-2H-pyran (0.77 mL, 5.10 mmol) was added and the mixture stirred at 20 °C for 3 days. The reaction was incomplete so the temperature was increased to 65°C and the reaction mixture was stirred for a further 2 days. The reaction mixture was quenched with saturated NH4Cl (5 mL) and extracted with EtOAc (15 mL). The organic layer was washed sequentially with water (2x 5 mL), saturated brine (2 mL) and dried over $MgSO_4$, filtered and evaporated to afford a pale yellow oil. The crude product was purified by flash silica chromatography, elution gradient 20 to 80% EtOAc in heptane to the title compound (0.580 g, 49.5 %) as a colourless oil; ^1H NMR (400 MHz, CDCl3, 30°C) 0.20 (1H, qd), 0.80 (1H, tdd), 1.3 – 1.44 (2H, m), 1.46 – 1.68 (3H, m), 1.71 (1H, ddd), 1.85 (1H, tt), 3.1 – 3.34 (3H, m), 3.43 – 3.67 (4H, m), 3.67 – 3.79 (1H, m), 3.8 – 4.02 (4H, m), 4.63 (1H, q).

Ethyl 2-cis-2-((2-((tetrahydro-2H-pyran-2-yl)oxy)ethoxy)methyl)cyclopropyl)methoxy)acetate (11b)

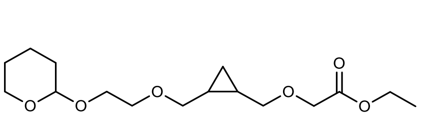

A solution of ethyl diazoacetate (0.775 mL, 6.30 mmol) in DCM (3 mL) was added dropwise to rhodium (II) acetate dimer (0.056 g, 0.13 mmol) and cis-2-((2-((tetrahydro-2H-pyran-2-yl)oxy)ethoxy)methyl)cyclopropyl)methanol (0.58 g, 2.52 mmol) in DCM (10 mL) cooled to 0°C over a period of 10 minutes under nitrogen. The resulting mixture was stirred at 20 °C for 30 minutes. The reaction was incomplete and further ethyl diazoacetate (0.775 mL, 6.30 mmol) was added dropwise at 20 °C and the mixture was stirred at 20 °C for a further 15 minutes. The reaction mixture was loaded directly on to a silica column and purified by flash silica chromatography, elution gradient 5 to 40% EtOAc in heptane. Pure fractions were evaporated to dryness to afford the title compound as a pale yellow oil; ^{1}H NMR (400 MHz, CDCl3, 30°C) 0.25 – 0.42 (1H, m), 0.87 (1H, td), 1.27 (7H, dt), 1.45 – 1.59 (3H, m), 1.72 (1H, ddt), 1.83 (1H, dq), 3.42 – 3.73 (7H, m), 3.86 (2H, ddd), 4.10 (2H, s), 4.22 (2H, q), 4.51 – 4.75 (1H, m); *m/z*: ES+ $[M+Na]^+$ 339.0.

Ethyl 2-((cis-2-((2-hydroxyethoxy)methyl)cyclopropyl)methoxy)acetate (11c)

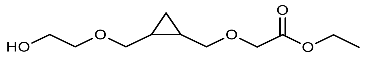

A solution of 2M aq. hydrogen chloride (1 mL, 2.00 mmol) was added to ethyl 2-cis-2-((2-((tetrahydro-2H-pyran-2-yl)oxy)ethoxy)methyl)cyclopropyl)methoxy)acetate (0.45 g, 1.42 mmol), in ethanol (5 mL) at 20°C under air. The resulting solution was stirred at 20 °C for 18 hours. The reaction mixture was evaporated to dryness. The crude product was purified by flash silica chromatography, elution gradient 20 to 100% EtOAc in heptane. Pure fractions were evaporated to dryness to afford the title compound (0.187 g, 56.6 %) as a colourless oil; ^{1}H NMR (400 MHz, CDCl3, 30°C) 0.24 (1H, q), 0.85 (1H, td), 1.21 – 1.37 (5H, m), 2.48 (1H, s), 3.49 – 3.56 (2H, m), 3.58 – 3.7 (4H, m), 3.7 – 3.75 (2H, m), 4.08 – 4.13 (2H, m), 4.22 (2H, q); *m/z*: ES+ $[M+H]^+$ 233.0

Ethyl 2-[[(cis)-2-[2-[3,5-difluoro-4-[(1R,3R)-2-(2-fluoro-2-methyl-propyl)-3-methyl-1,3,4,9-tetrahydropyrido[3,4-b]indol-1-yl]phenoxy]ethoxymethyl]cyclopropyl]methoxy]acetate (11d)

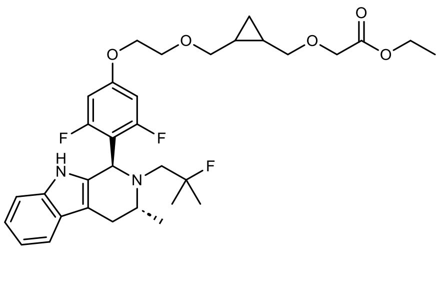

DIAD (0.229 mL, 1.16 mmol) was added to ethyl 2-((cis-2-((2-hydroxyethoxy)methyl)cyclopropyl)methoxy)acetate, 3,5-difluoro-4-((1R,3R)-2-(2-fluoro-2-methylpropyl)-3-methyl-2,3,4,9-tetrahydro-1H-pyrido[3,4-b]indol-1-yl)phenol (391 mg, 1.01 mmol) and triphenylphosphine (407 mg, 1.55 mmol) in DCM cooled to 0°C over a period of 1 minute under nitrogen. The resulting solution was stirred at 20 °C for 15 minutes. The reaction was incomplete and further triphenylphosphine (407 mg, 1.55 mmol) and DIAD (0.229 mL, 1.16 mmol) were added sequentially at 0°C and the solution was stirred at 20 °C for a further 15 minutes. The reaction mixture was evaporated to dryness. The crude product was purified by flash silica chromatography, elution gradient 0 to 40% EtOAc in heptane. Pure fractions were evaporated to dryness. The residue was dissolved in ethanol and purified by ion exchange chromatography, using an SCX column. The desired product was eluted from the column using .880 ammonia:EtOH (1:9) and pure fractions were evaporated to dryness to afford crude product. The crude product was purified by flash silica chromatography, elution gradient 5 to 40% EtOAc in heptane. Pure fractions were evaporated to dryness to afford the title compound (222 mg, 48 %) as a colourless oil; ^{1}H NMR (400 MHz, CDCl3, 30°C) 0.30 (1H, dt), 0.82 – 0.93 (1H, m), 1.10 (3H, d), 1.13 – 1.33 (12H, m), 2.35 (1H, s), 2.60 (1H, dd), 2.87 (1H, dd), 3.09 (1H, dd), 3.45 – 3.6 (3H, m), 3.66 (2H, ddt), 3.72 – 3.9 (2H, m), 4.05 – 4.11 (4H, m), 4.20 (2H, qd), 5.18 (1H, s), 6.43 (2H, d), 7.04 – 7.13 (2H, m), 7.15 – 7.17 (2H, m), 7.27 (1H, d), 7.49 – 7.53 (1H, m), 7.59 (1H, d); *m/z*: ES- $[M-H]^-$ 601.3.

2-[[(cis)-2-[2-[3,5-Difluoro-4-[(1R,3R)-2-(2-fluoro-2-methyl-propyl)-3-methyl-1,3,4,9-tetrahydropyrido[3,4-b]indol-1-yl]phenoxy]ethoxymethyl]cyclopropyl]methoxy]acetic acid (11e)

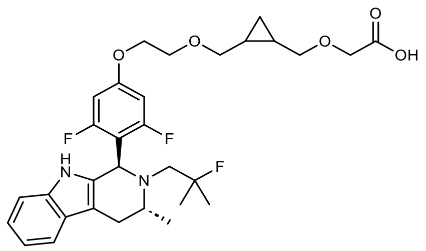

A solution of 2M aq. sodium hydroxide (0.365 mL, 0.73 mmol) was added to ethyl 2-[[(cis)-2-[2-[3,5-difluoro-4-[(1R,3R)-2-(2-fluoro-2-methyl-propyl)-3-methyl-1,3,4,9-tetrahydropyrido[3,4-b]indol-1-yl]phenoxy]ethoxymethyl]cyclopropyl]methoxy]acetate (0.22 g, 0.37 mmol) in ethanol (3 mL) at 20°C under air. The resulting solution was stirred at 20 °C for 1 hour. The reaction mixture was acidified with 2M HCl (1 mL), diluted with EtOAc (15 mL), and washed sequentially with water (2x 2 mL) and saturated brine (2 mL). The organic layer was dried with $MgSO_4$, filtered and evaporated to afford the title compound (0.158 g, 75 %) as a yellow solid that was used without further purification; ^{1}H NMR

(400 MHz, CDCl3, 30°C) 0.25 (1H, p), 0.83 – 0.96 (1H, m), 1.04 – 1.23 (7H, m), 1.3 – 1.43 (3H, m), 2.35 (1H, s), 2.61 (1H, d), 2.87 (1H, s), 3.08 (1H, s), 3.26 – 3.46 (2H, m), 3.63 – 3.75 (1H, m), 3.81 (2H, s), 3.86 – 3.96 (2H, m), 3.97 – 4.23 (5H, m), 5.19 (1H, s), 6.41 (2H, d), 7.09 (2H, s), 7.24 (1H, s), 7.43 – 7.58 (1H, m), 7.71 (1H, s). Assigned Hs: 36. Missing 1 proton; *m/z*: ES+ $[M+H]^+$ 575.0

(2S,4R)-1-[(2S)-2-[[2-[[(cis)-2-[2-[3,5-Difluoro-4-[(1R,3R)-2-(2-fluoro-2-methyl-propyl)-3-methyl-1,3,4,9-tetrahydropyrido[3,4-b]indol-1-yl]phenoxy]ethoxymethyl]cyclopropyl]methoxy]acetyl]amino]-3,3-dimethyl-butanoyl]-4-hydroxy-N-[[4-(4-methylthiazol-5-yl)phenyl]methyl]pyrrolidine-2-carboxamide (11f)

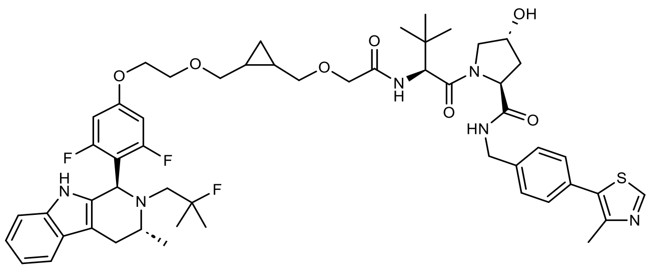

HATU (0.154 g, 0.40 mmol) was added to 2-[[(cis)-2-[2-[3,5-difluoro-4-[(1R,3R)-2-(2-fluoro-2-methyl-propyl)-3-methyl-1,3,4,9-tetrahydropyrido[3,4-b]indol-1-yl]phenoxy]ethoxymethyl]cyclopropyl]methoxy]acetic acid (0.155 g, 0.27 mmol), (2S,4R)-1-((S)-2-amino-3,3-dimethylbutanoyl)-4-hydroxy-N-(4-(4-methylthiazol-5-yl)benzyl)pyrrolidine-2-carboxamide, 2HCl (0.177 g, 0.35 mmol) and HATU (0.154 g, 0.40 mmol) in DMF (2 mL) at 20°C under air. The resulting suspension was stirred at 20 °C for 3 days. The reaction mixture was diluted with EtOAc (20 mL), and washed sequentially with water (4x 3 mL) and saturated brine (2x 1 mL). The organic layer was dried with MgSO4, filtered and evaporated to afford crude product. The crude product was purified by flash silica chromatography, elution gradient 0.5 to 8% MeOH in DCM. Pure fractions were evaporated to dryness to afford the title compound (0.201 g, 75 %) (0.201 g, 75 %) as a yellow solid; ^{1}H NMR (400 MHz, CDCl3, 30°C) 0.24 (q, J = 5.5 Hz, 1H), 0.36 (q, J = 5.5 Hz, 1H), 0.86 (ddd, J = 11.4, 8.3, 4.1 Hz, 1H), 0.95 (s, 9H), 1.09 (d, J = 6.5 Hz, 4H), 1.13 – 1.34 (m, 9H), 2.08 (dd, J = 12.8, 6.7 Hz, 1H), 2.40 (dd, J = 25.3, 15.6 Hz, 1H), 2.46 – 2.65 (m, 5H), 2.84 (dd, J = 19.2, 16.0 Hz, 2H), 3.07 (dd, J = 15.0, 4.8 Hz, 1H), 3.35 – 3.76 (m, 10H), 3.81 (ddd, J = 11.4, 5.7, 3.0 Hz, 1H), 3.90 (dd, J = 17.7, 15.5 Hz, 1H), 4.03 (tt, J = 9.5, 4.8 Hz, 3H), 4.33 (dd, J = 14.9, 5.2 Hz, 1H), 4.43 – 4.63 (m, 3H), 4.70 (t, J = 7.8 Hz, 1H), 5.19 (s, 1H), 6.38 (dd, J = 10.6, 2.3 Hz, 2H), 7.07 (ddd, J = 6.3, 2.8, 1.9 Hz, 2H), 7.21 (td, J = 4.8, 4.0, 2.2 Hz, 3H), 7.32 – 7.44 (m, 4H), 7.50 (dd, J = 5.5, 3.3 Hz, 1H), 8.52 (s, 0H), 8.60 (s, 0H), 8.62 – 8.72 (m, 1H). *m/z*: ES- $[M-H]^-$ 985.5.

(2S,4R)-1-[(2S)-2-[[2-[[(cis)-2-[2-[3,5-Difluoro-4-[(1R,3R)-2-(2-fluoro-2-methyl-propyl)-3-methyl-1,3,4,9-tetrahydropyrido[3,4-b]indol-1-yl]phenoxy]ethoxymethyl]cyclopropyl]methoxy]acetyl]amino]-3,3-dimethyl-butanoyl]-4-hydroxy-N-[[4-(4-methylthiazol-5-yl)phenyl]methyl]pyrrolidine-2-carboxamide, isomer 1 (11)

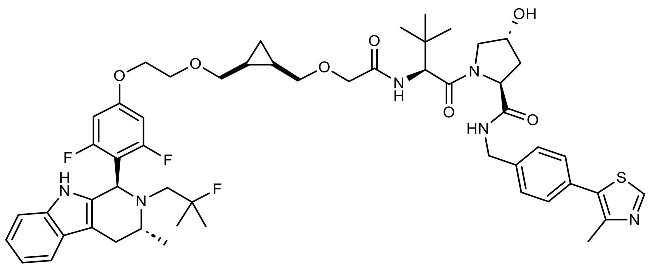

(2S,4R)-1-[(2S)-2-[[2-[[(cis)-2-[2-[3,5-Difluoro-4-[(1R,3R)-2-(2-fluoro-2-methyl-propyl)-3-methyl-1,3,4,9-tetrahydropyrido[3,4-b]indol-1-yl]phenoxy]ethoxymethyl]cyclopropyl]methoxy]acetyl]amino]-3,3-dimethyl-butanoyl]-4-hydroxy-N-[[4-(4-methylthiazol-5-yl)phenyl]methyl]pyrrolidine-2-carboxamide (200 mg, 0.2 mmol) was dissolved in MeOH. The resulting solution was purified using the SFC conditions detailed below: Column: YMC Amylose C, 20 x 250 mm, 5 micron; Mobile phase: 35 % EtOH = 0.1% NH3 / 65 % scCO2; Flow rate: 60 ml/min; BPR: 120 bar; Column temperature: 40 °C to afford the title compound that eluted first from the column (14 mg, 5 %) as a yellow solid; ^{1}H NMR (400 MHz, CDCl3, 30°C) 0.24 (1H, q), 0.72 (1H, t), 0.86 (4H, dq), 0.95 (9H, s), 1.09 (3H, d), 1.20 (6H, dd), 2.07 (1H, dd), 2.40 (1H, dd), 2.49 (3H, s), 2.58 (2H, ddd), 2.85 (1H, dd), 3.07 (1H, dd), 3.38 – 3.55 (3H, m), 3.58 – 3.76 (5H, m), 3.77 – 3.91 (2H, m), 3.97 – 4.12 (3H, m), 4.33 (1H, dd), 4.5 – 4.61 (3H, m), 4.72 (1H, t), 5.19 (1H, s), 6.39 (2H, d), 7.02 – 7.14 (2H, m), 7.19 – 7.25 (2H, m), 7.31 – 7.4 (4H, m), 7.46 – 7.53 (1H, m), 8.61 (1H, s), 8.66 (1H, s); *m/z*: ES- $[M-H]^-$ 985.5; LCMS purity = 100%.

(2S,4R)-1-[(2S)-2-[[2-[[(cis)-2-[2-[3,5-Difluoro-4-[(1R,3R)-2-(2-fluoro-2-methyl-propyl)-3-methyl-1,3,4,9-tetrahydropyrido[3,4-b]indol-1-yl]phenoxy]ethoxymethyl]cyclopropyl]methoxy]acetyl]amino]-3,3-dimethyl-butanoyl]-4-hydroxy-N-[[4-(4-methylthiazol-5-yl)phenyl]methyl]pyrrolidine-2-carboxamide, isomer 2 (12)

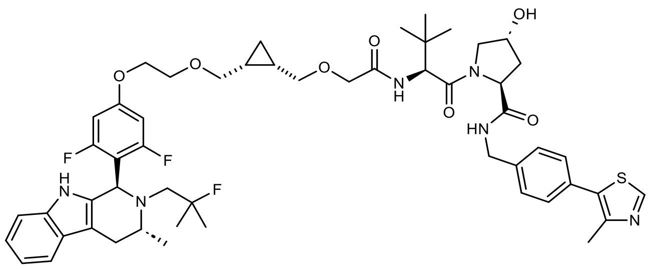

The title compound was isolated using the same methodology as **11** collecting the compound that eluted second from the column (37 mg, 14 %) as a yellow solid; ^{1}H NMR (400 MHz, CDCl3, 30°C) 0.37 (1H, q), 0.82 – 0.91 (2H, m), 0.95 (9H, s), 1.09 (3H, d), 1.20 (7H, dd), 1.28 – 1.31 (1H, m), 2.03 – 2.14 (1H, m), 2.40 (1H, dd), 2.50 (3H, s), 2.52 – 2.64 (2H, m), 2.84 (1H, dd), 3.07 (1H, dd), 3.42 (2H, ddd), 3.54 – 3.76 (6H, m), 3.78 – 3.87 (1H, m), 3.92 (1H, d), 3.95 – 4.13 (3H, m), 4.33 (1H, dd), 4.47 – 4.63 (3H, m), 4.71 (1H, t), 5.20 (1H, s), 6.38 (2H, d), 7 – 7.13 (2H, m), 7.17 – 7.26 (3H, m), 7.3 – 7.42 (4H, m), 7.50 (1H, dd), 8.53 (1H, s), 8.66 (1H, s); ^{13}C NMR (126 MHz, DMSO-d6, 27°C) 8.2, 12.4, 14.5, 14.7, 15.6, 24.2, 24.6, 25.9, 26.4, 35.5, 37.6, 41.4, 50.2 - 50.3, 55.4, 55.8, 56.0, 58.5, 67.6, 67.7, 68.6, 68.8, 69.8, 70.5, 96.7, 98.4, 106.1, 109.0, 110.6, 117.1, 117.8, 120.0, 126.7, 127.2, 128.4, 129.4, 130.8, 132.4, 135.9, 139.1, 147.5, 151.1, 159.2, 161.9, 168.3, 168.9, 171.4.; *m/z*: ES- $[M-H]^-$ 985.6; LCMS purity = 100%; ESI-HRMS calculated for $C_{53}H_{66}F_3N_6O_7S$ $[M+H]^+$: 987.4660, observed 987.4611.

Ethyl 1-(3-(3-(benzyloxy)propoxy)propoxy)cyclopropane-1-carboxylate (13)

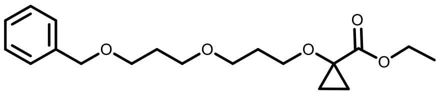

Trifluoromethanesulfonic anhydride (0.47 mL, 2.8 mmol) was added dropwise to a stirred solution of 3-(3-(benzyloxy)propoxy)propan-1-ol (575 mg, 2.56 mmol), and 2,6-dimethylpyridine (0.33 mL, 2.8 mmol) in DCM (6 mL) at -78°C, under nitrogen. The resulting solution was stirred at -78 °C for 1 hour. The reaction mixture was diluted with DCM (25 mL), and washed sequentially with saturated NH4Cl (20 mL) and water (25 mL). The organic layer was dried with a phase separating cartridge, filtered and evaporated to afford crude 3-(3-(benzyloxy)propoxy)propyl trifluoromethanesulfonate that was dissolved in THF (9.42 mL). Ethyl 1-hydroxycyclopropane-1-carboxylate (0.311 ml, 2.56 mmol) was added to the mixture and cooled to -78°C under nitrogen. A solution of 1 N lithium bis(trimethylsilyl)amide in THF (3.07 ml, 3.07 mmol) was added dropwise to the solution over 10 minutes. The resulting mixture was stirred at -78 °C for 40 minutes and warmed to 0°C. The reaction mixture was warmed to room temperature and stirred at 25°C for 2 days. The reaction mixture was diluted with saturated NH4Cl (30 mL), ethyl acetate (50 mL) and 5 mL of 2N HCl. The layers were

separated and the aqueous phase back extracted with ethyl acetate (3 x 30 mL). The combined organic phases were washed with brine (50 mL), dried over $MgSO_4$, filtered and concentrated to a crude oil. The crude product was purified by flash silica chromatography, elution gradient 0 to 20% EtOAc in heptane to afford the title compound (213 mg, 25 %) as a colourless oil; ^{1}H NMR (400 MHz, CDCl3, 30°C) 1.09 – 1.15 (2H, m), 1.24 – 1.32 (5H, m), 1.81 (2H, p), 1.88 (2H, q), 3.47 (2H, t), 3.51 (2H, t), 3.56 (2H, t), 3.66 (2H, t), 4.19 (2H, q), 4.50 (2H, s), 7.26 – 7.37 (5H, m).

Ethyl 1-(3-(3-hydroxypropoxy)propoxy)cyclopropane-1-carboxylate (13b)

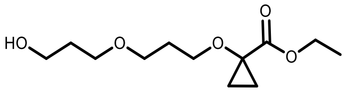

A suspension of ethyl 1-(3-(3-(benzyloxy)propoxy)propoxy)cyclopropane-1-carboxylate (213mg, 0.63 mmol) and 10% palladium on carbon (131 mg, 0.06 mmol) in ethanol (6 mL) was stirred under 1 atmosphere of hydrogen at 25 °C for 17 hours. The reaction mixture was filtered through celite, washing with EtOAc (3 x 20 mL), and the filtrate was evaporated to afford the title compound (162 mg) as a colourless oil that was used without further purification; ^{1}H NMR (400 MHz, CDCl3, 30°C) 1.06 – 1.17 (2H, m), 1.24 – 1.34 (5H, m), 1.74 – 1.92 (4H, m), 2.43 (1H, s), 3.53 (2H, t), 3.62 (2H, t), 3.67 (2H, t), 3.77 (2H, q), 4.19 (2H, q).

Ethyl 1-[3-(3-{3,5-difluoro-4-[(1*R*,3*R*)-2-(2-fluoro-2-methylpropyl)-3-methyl-2,3,4,9-tetrahydro-1*H*-pyrido[3,4-*b*]indol-1-yl]phenoxy}propoxy)propoxy]cyclopropane-1-carboxylate (13c)

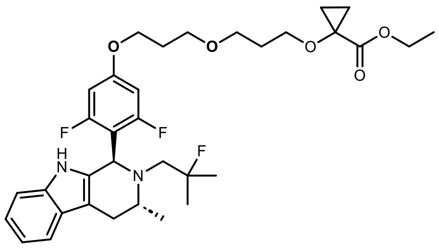

DIAD (0.233 mL, 1.18 mmol) was added dropwise to a stirred solution of 3,5-difluoro-4-((1R,3R)-2-(2-fluoro-2-methylpropyl)-3-methyl-2,3,4,9-tetrahydro-1H-pyrido[3,4-b]indol-1-yl)phenol (230 mg, 0.59 mmol), ethyl 1-(3-(3-hydroxypropoxy)propoxy)cyclopropane-1-carboxylate (160 mg, 0.65 mmol) and triphenylphosphane (311 mg, 1.18 mmol) in DCM (8.23 mL) at rt, under nitrogen. The resulting solution was stirred at rt for 17 hours. Further portions of 3,5-difluoro-4-((1R,3R)-2-(2-fluoro-2-methylpropyl)-3-methyl-2,3,4,9-tetrahydro-1H-pyrido[3,4-b]indol-1-yl)phenol (230 mg, 0.59 mmol),

triphenylphosphane (311 mg, 1.18 mmol) and DIAD (0.233 mL, 1.18 mmol) were added to the reaction mixture and stirred for a further 2 hours. The reaction mixture was evaporated and evaporated to afford crude product. The crude product was purified by flash silica chromatography, elution gradient 0 to 40% EtOAc in heptane. Pure fractions were evaporated to dryness to afford the title compound (73 mg, 20 %) as a colourless oil; ^{1}H NMR (400 MHz, CDCl3, 30°C) 1.02 – 1.14 (5H, m), 1.17 (3H, d), 1.2 – 1.3 (8H, m), 1.81 (2H, p), 2.01 (2H, p), 2.39 (1H, dd), 2.60 (1H, dd), 2.64 – 2.7 (1H, m), 2.86 (1H, dd), 3.09 (1H, dd), 3.50 (2H, t), 3.56 (2H, t), 3.65 (3H, dtt), 4.01 (2H, tq), 4.09 – 4.24 (2H, m), 5.19 (1H, s), 6.3 – 6.49 (2H, m), 7.02 – 7.14 (2H, m), 7.17 – 7.23 (1H, m), 7.43 – 7.58 (1H, m); *m/z*: ES+ $[M+H]^+$ 617.3.

(2S,4R)-1-((S)-2-(1-(3-(3-(3,5-Difluoro-4-((1R,3R)-2-(2-fluoro-2-methylpropyl)-3-methyl-2,3,4,9-tetrahydro-1H-pyrido[3,4-b]indol-1-yl)phenoxy)propoxy)propoxy)cyclopropane-1-carboxamido)-3,3-dimethylbutanoyl)-4-hydroxy-N-(4-(4-methylthiazol-5-yl)benzyl)pyrrolidine-2-carboxamide (13)

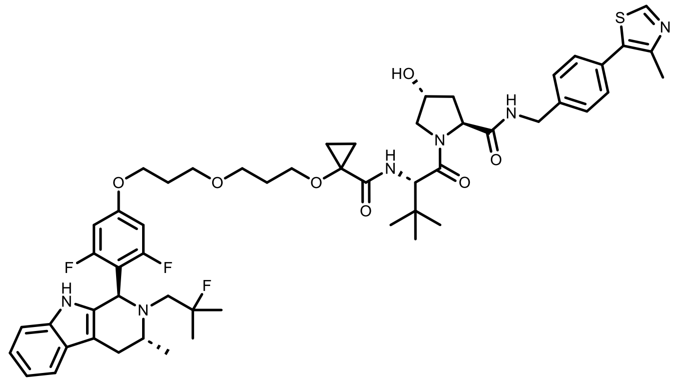

A solution of 1 M lithium hydroxide hydrate (0.24 ml, 0.24 mmol) was added to a stirred solution of ethyl 1-(3-(3-(3,5-difluoro-4-((1R,3R)-2-(2-fluoro-2-methylpropyl)-3-methyl-2,3,4,9-tetrahydro-1H-pyrido[3,4-b]indol-1-yl)phenoxy)propoxy)propoxy)cyclopropane-1-carboxylate (0.073 g, 0.12 mmol) in EtOH (1 ml). The resulting solution was stirred at rt for 1 hour. A further aliquot of 1M lithium hydroxide hydrate solution (0.24 mL, 0.24 mmol) was added to the reaction mixture and left to stir for a further hour. The reaction mixture was partially evaporated, the aqueous residue was adjusted to pH 5 with 2M HCl, diluted with water (15 mL) and extracted with EtOAc (2 x 25 mL). The organic extracts were combined, washed with saturated brine (25 mL) and evaporated to afford a colourless gum. The gum was dissolved in DMF (2.5 mL), (2S,4R)-1-((S)-2-amino-3,3-dimethylbutanoyl)-4-hydroxy-N-(4-(4-methylthiazol-5-yl)benzyl)pyrrolidine-2-carboxamide, 2HCl (0.043 g, 0.09 mmol) and triethylamine (0.048 mL, 0.34 mmol) added. HATU (0.049 g, 0.13 mmol) was added under nitrogen.

The resulting solution was stirred at RT for 17 hours. The reaction mixture was diluted with water (10 mL) and extracted with EtOAc (25 mL). The organic extract was washed with saturated brine (25 mL), dried with a phase separating cartridge, filtered and evaporated to afford crude product. The crude product was purified by preparative HPLC (Waters CSH C18 OBD column, 30 x 100 mm id, 5 micron particle size), using decreasingly polar mixtures of water (containing 0.1% NH3 aq) and MeCN as eluents to afford the impure compound. The sample (20 mg) was purified using the SFC conditions detailed: Column: Princeton Diol, 30 x 250 mm, 5 micron; Mobile phase: 25 to 45% MeOH + 0.1% NH3 / 75-55% scCO2 over 8 minutes; Flow rate: 100 ml/min; BPR: 120 bar; Column temperature: 40 °C and evaporated to dryness to afford the title compound (14 mg, 17 %) as a white solid. ^{1}H NMR (400 MHz, CDCl3, 30°C) 0.93 (1H, d), 0.96 (9H, s), 1.02 (1H, dd), 1.06 (1H, dd), 1.10 (3H, d), 1.20 (7H, dd), 1.76 – 1.89 (2H, m), 1.98 (2H, p), 2.07 (1H, dd), 2.41 (1H, dd), 2.48 (3H, s), 2.52 – 2.65 (2H, m), 2.77 – 2.9 (2H, m), 3.07 (1H, dd), 3.42 – 3.61 (7H, m), 3.61 – 3.72 (1H, m), 3.95 (2H, tt), 4.08 (1H, d), 4.32 (1H, dd), 4.47 (1H, d), 4.51 (1H, s), 4.57 (1H, dd), 4.73 (1H, t), 5.20 (1H, s), 6.31 – 6.37 (2H, m), 7.04 – 7.11 (2H, m), 7.18 – 7.25 (2H, m), 7.3 – 7.39 (5H, m), 7.48 – 7.53 (1H, m), 8.34 (1H, s), 8.63 (1H, s); ^{13}C NMR (126 MHz, DMSO-d6, 27°C) 13.4, 13.7, 15.3, 16.4, 25.0, 25.3, 26.7, 27.1, 29.3, 30.1, 36.6, 38.3, 42.2, 50.9 - 51.2, 56.5, 56.9, 57.1, 59.3, 62.5, 65.9, 66.9, 67.1, 67.4, 69.4, 97.4, 99.1, 106.8, 109.7, 111.4, 117.9, 118.6, 120.8, 127.4, 128.0, 129.2, 130.2, 131.6, 133.1, 136.7, 139.9, 148.2, 151.9, 160.0, 162.7, 169.7, 171.1, 172.1; *m/z*: ES- $[M-H]^-$ 999.1; LCMS purity = 100%; ESI-HRMS calculated for calculated for $C_{54}H_{68}F_3N_6O_7S$ $[M+H]^+$: 1001.4817, observed 1001.4745.

Methyl 3-(3-hydroxyprop-1-yn-1-yl)benzoate (14a)

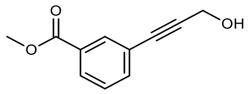

Bis(triphenylphosphine)palladium(II) dichloride (0.67 g, 0.95 mmol) was added in one portion to methyl 3-iodobenzoate (5 g, 19.08 mmol) and copper(I) iodide (0.18 g, 0.95 mmol) in degassed THF (90 mL) at 20 °C under nitrogen. The resulting mixture was stirred for 5 minutes then prop-2-yn-1-ol (2.22 mL, 38.16 mmol) and triethylamine (5.31 mL, 38.16 mmol) was added. The reaction was stirred at 20 °C for 18 hours. The reaction mixture was diluted with EtOAc (200 mL), and washed sequentially with water (2 x 100 mL) and saturated brine (50 mL). The organic layer was dried with $MgSO_4$, filtered and evaporated to afford crude product. The crude product was purified by flash silica chromatography, elution gradient 0 to 50% EtOAc in heptane to afford the title compound (3.10 g, 85 %) as a yellow solid; ^{1}H NMR (400 MHz, $CDCl_3$, 30°C) 1.69 (1H, t), 3.92 (3H, s), 4.51 (2H, d), 7.40 (1H, t), 7.61 (1H, dt), 7.99 (1H, dt), 8.11 (1H, d).

Methyl 3-(3-hydroxypropyl)benzoate (14b)

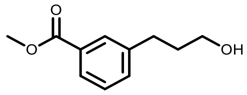

Methyl 3-(3-hydroxyprop-1-yn-1-yl)benzoate (3.1 g, 16.30 mmol) and 10% palladium on carbon (0.173 g, 1.63 mmol) in ethyl acetate (30 mL) were stirred under 1.5 bar of hydrogen at RT for 18 hours. Catalyst was filtered off and replaced with fresh catalyst and the reaction charged with hydrogen as before and stirred for a further 24 hours. The reaction mixture was filtered, washing with EtOAc then the solvent was evaporated to afford the title compound (3.08 g, 97 %) as a pale yellow oil, which was used in the next step without further purification; ^{1}H NMR (400 MHz, $CDCl_3$, 30°C) 1.86 – 1.98 (2H, m), 2.74 – 2.8 (2H, m), 3.68 (2H, t), 3.91 (3H, s), 7.37 (2H, dt), 7.83 – 7.94 (2H, m).

Methyl 3-(3-((tert-butyldimethylsilyl)oxy)propyl)benzoate (14c)

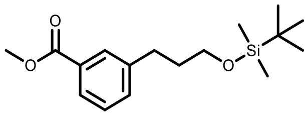

tert-Butylchlorodimethylsilane (1.69 g, 11.2 mmol) was added in one portion to methyl 3-(3-hydroxypropyl)benzoate (1.98 g, 10.2 mmol) and 1*H*-imidazole (0.76 g, 11.2 mmol) in DCM (16 mL) at 20 °C under nitrogen. The resulting white suspension was stirred for 2 hours. The reaction mixture was diluted with DCM (100 mL), and washed with water (50 mL) and saturated brine (100 mL). The organic layer was dried with $MgSO_4$, filtered and evaporated to afford crude product. The crude product was purified by flash silica chromatography, elution gradient 0 to 5% EtOAc in heptane to afford the title compound (1.28 g, 41 %) as a colourless oil; ^{1}H NMR (400 MHz, $CDCl_3$, 30°C) 0.05 (6H, s), 0.91 (9H, s), 1.8 – 1.89 (2H, m), 2.69 – 2.78 (2H, m), 3.63 (2H, t), 3.91 (3H, s), 7.3 – 7.43 (2H, m), 7.82 – 7.92 (2H, m); *m/z*: ES+ $[M+H]^+$ 309.3

(3-(3-((*tert*-Butyldimethylsilyl)oxy)propyl)phenyl)methanol (14d)

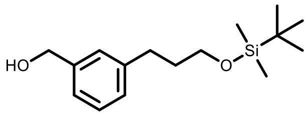

A solution of lithium aluminum hydride 1M in THF (5.39 mL, 5.39 mmol) was added dropwise to a solution of methyl 3-(3-((tert-butyldimethylsilyl)oxy)propyl)benzoate (1.28 g, 4.15 mmol) in THF (15 mL) at 0 °C over a period of 15 minutes under nitrogen. The resulting mixture was stirred at 20 °C for 2 hours. The reaction mixture was cooled to 0 °C and quenched with careful dropwise addition of water (0.18 mL), 2M NaOH solution (0.36 mL) and water (0.54 mL). The mixture was stirred for 5 minutes. The solids were removed by filtration and the filtrate was evaporated to dryness. The crude product was purified by flash silica chromatography, elution gradient 0 to 25% EtOAc in heptane to afford the title compound (0.788 g, 68 %) as a colourless liquid; ^{1}H NMR (400 MHz, $CDCl_3$, 30°C) 0.05 (6H, s), 0.91 (9H, s), 1.58 (1H, t), 1.79 – 1.9 (2H, m), 2.65 – 2.72 (2H, m), 3.63 (2H, t), 4.67 (2H, d), 7.13 (1H, d), 7.15 – 7.23 (2H, m), 7.28 (1H, d); *m/z*: ES+ $[M+H]^+$ 281.3.

(1*R*,3*R*)-1-(4-((3-(3-((*tert*-Butyldimethylsilyl)oxy)propyl)benzyl)oxy)-2,6-difluorophenyl)-2-(2-fluoro-2-methylpropyl)-3-methyl-2,3,4,9-tetrahydro-1*H*-pyrido[3,4-b]indole (14e)

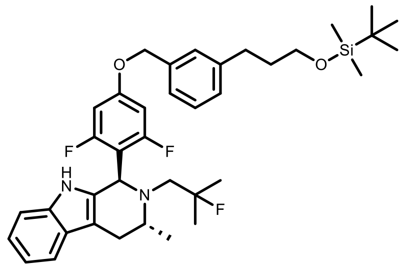

DIAD (0.253 mL, 1.29 mmol) was added dropwise over 15 minutes to a stirred mixture of 3,5-difluoro-4-((1R,3R)-2-(2-fluoro-2-methylpropyl)-3-methyl-2,3,4,9-tetrahydro-1H-pyrido[3,4-b]indol-1-yl)phenol (250mg, 0.64 mmol), (3-(3-((tert-butyldimethylsilyl)oxy)propyl)phenyl)methanol (361 mg, 1.29 mmol) and triphenylphosphine (338 mg, 1.29 mmol) in DCM (6.284 mL) at 0 °C. The resulting mixture was stirred at room termperature for 1 hour. DCM (50 mL) and water (25 mL) were added and the layers were separated. The DCM layer was passed through a phase separating cartridge and concentrated to give the crude product as an orange oil. The crude product was purified by flash silica chromatography, elution gradient 0 to 25% EtOAc in heptane. Product containing fractions were evaporated to dryness to afford the title compound (333 mg, 79 %) as a pale yellow gum; ^{1}H NMR (400 MHz, $CDCl_3$, 30°C) 0.05 (6H, s), 0.91 (9H, s), 1.10 (3H, d), 1.20 (6H, dd), 1.79 – 1.89 (2H, m), 2.39 (1H, dd), 2.60 (1H, dd), 2.66 – 2.74 (2H, m), 2.86 (1H, dd), 3.05 – 3.14 (1H, m), 3.6 – 3.73 (3H, m), 4.98 (2H, s), 5.19 (1H, s), 6.48 (2H, d), 7.05 – 7.14 (2H, m), 7.15 – 7.25 (4H, m), 7.30 (1H, t), 7.41 (1H, s), 7.48 – 7.55 (1H, m); *m/z*: ES+ $[M+H]^+$ 651.4.

3-(3-((3,5-Difluoro-4-((1*R*,3*R*)-2-(2-fluoro-2-methylpropyl)-3-methyl-2,3,4,9-tetrahydro-1*H*-pyrido[3,4-b]indol-1-yl)phenoxy)methyl)phenyl)propan-1-ol (14f)

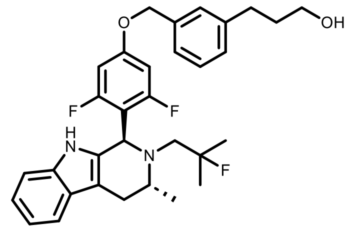

TBAF 1M in THF (0.77 mL, 0.77 mmol) was added in one portion to (1*R*,3*R*)-1-(4-((3-(3-((*tert*-butyldimethylsilyl)oxy)propyl)benzyl)oxy)-2,6-difluorophenyl)-2-(2-fluoro-2-methylpropyl)-3-methyl-2,3,4,9-tetrahydro-1*H*-pyrido[3,4-b]indole (333mg, 0.51 mmol) in THF (4 mL) at 20 °C. The resulting solution was stirred for 2 hours. The reaction mixture was diluted with EtOAc (50 mL), and washed sequentially with saturated NH_4Cl (20 mL), water (20 mL), and saturated brine (20 mL). The organic layer was dried with $MgSO_4$, filtered and evaporated to afford crude product. The crude product was purified by flash silica chromatography, elution gradient 0 to 50% EtOAc in heptane to afford the title compound (207 mg, 75 %) as a yellow gum; ^{1}H NMR (400 MHz, $CDCl_3$, 30°C) 1.10 (3H, d), 1.14 – 1.23 (6H, m), 1.86 – 1.95 (2H, m), 2.39 (1H, dd), 2.53 – 2.65 (1H, m), 2.7 – 2.77 (2H, m), 2.8 – 2.91 (1H, m), 3.09 (1H, d), 3.64 (3H, q), 5.01 (2H, s), 5.19 (1H, s), 6.47 (2H, d), 7.04 – 7.15 (2H, m), 7.14 – 7.25 (4H, m), 7.31 (1H, t), 7.47 – 7.57 (2H, m) (OH not observed in NMR); *m/z*: ES+ $[M+H]^+$ 537.4.

Ethyl 2-(3-(3-((3,5-difluoro-4-((1*R*,3*R*)-2-(2-fluoro-2-methylpropyl)-3-methyl-2,3,4,9-tetrahydro-1*H*-pyrido[3,4-b]indol-1-yl)phenoxy)methyl)phenyl)propoxy)acetate (14g)

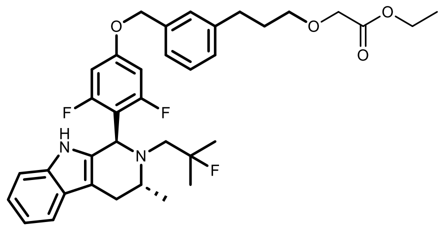

Ethyl 2-diazoacetate (0.061 mL, 0.58 mmol) in DCM (1.232 mL) was added slowly to 3-(3-((3,5-difluoro-4-((1R,3R)-2-(2-fluoro-2-methylpropyl)-3-methyl-2,3,4,9-tetrahydro-1H-pyrido[3,4-b]indol-1-yl)phenoxy)methyl)phenyl)propan-1-ol (207mg, 0.39 mmol) and rhodium acetate dimer (8.52 mg, 0.02 mmol) in DCM (3.202 mL) at 20°C over a period of 1.5 hours under nitrogen. The mixture was diluted with water (10 mL) and the DCM layer was separated and passed through phase separating filter and evaporated to dryness. The crude product was purified by flash silica chromatography, elution gradient 0 to 30% EtOAc in heptane. Pure fractions were evaporated to dryness to afford the

title compound (109 mg, 45 %) as a colourless gum; ^{1}H NMR (400 MHz, $CDCl_3$, 30°C) 1.10 (3H, d), 1.20 (6H, dd), 1.29 (3H, t), 1.89 – 2.01 (2H, m), 2.39 (1H, dd), 2.60 (1H, dd), 2.71 – 2.78 (2H, m), 2.86 (1H, dd), 3.09 (1H, d), 3.54 (2H, t), 3.69 (1H, s), 4.06 (2H, d), 4.22 (2H, q), 4.99 (2H, s), 5.19 (1H, s), 6.48 (2H, d), 7.05 – 7.15 (2H, m), 7.16 – 7.25 (4H, m), 7.31 (1H, t), 7.47 (1H, s), 7.49 – 7.55 (1H, m); *m/z*: ES+ $[M+H]^+$ 623.5.

2-(3-(3-((3,5-Difluoro-4-((1*R*,3*R*)-2-(2-fluoro-2-methylpropyl)-3-methyl-2,3,4,9-tetrahydro-1*H*-pyrido[3,4-b]indol-1-yl)phenoxy)methyl)phenyl)propoxy)acetic acid (14h)

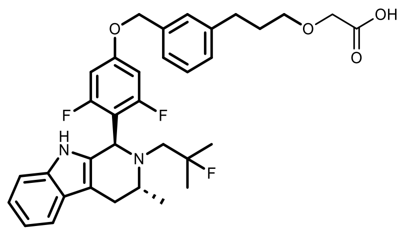

Lithium hydroxide hydrate (14.69 mg, 0.35 mmol) was added in one portion to ethyl 2-(3-(3-((3,5-difluoro-4-((1R,3R)-2-(2-fluoro-2-methylpropyl)-3-methyl-2,3,4,9-tetrahydro-1H-pyrido[3,4-b]indol-1-yl)phenoxy)methyl)phenyl)propoxy)acetate (109mg, 0.18 mmol) in THF (0.65 mL) and water (0.22 mL) at 20°C. The resulting solution was stirred at 20 °C for 30 minutes. The reaction mixture was diluted with water (10 mL) then was acidified with 2M HCl and extracted into EtOAc (50 mL). The organic layer was washed with brine (15 mL) and evaporated to afford the title compound (104 mg, 100 %) as a yellow gum which was used in the next step without further purification; *m/z*: ES+ $[M+H]^+$ 595.4.

(2*S*,4*R*)-1-((*S*)-2-(2-(3-(3-((3,5-Difluoro-4-((1*R*,3*R*)-2-(2-fluoro-2-methylpropyl)-3-methyl-2,3,4,9-tetrahydro-1*H*-pyrido[3,4-b]indol-1-yl)phenoxy)methyl)phenyl)propoxy)acetamido)-3,3-dimethylbutanoyl)-4-hydroxy-*N*-(4-(4-methylthiazol-5-yl)benzyl)pyrrolidine-2-carboxamide (14)

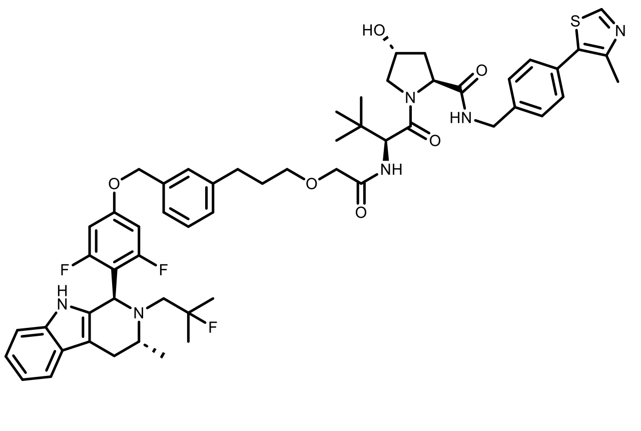

HATU (96 mg, 0.25 mmol) was added in one portion to 2-(3-(3-((3,5-difluoro-4-((1R,3R)-2-(2-fluoro-2-methylpropyl)-3-methyl-2,3,4,9-tetrahydro-1H-pyrido[3,4-b]indol-1-yl)phenoxy)methyl)phenyl)propoxy)acetic acid (100mg, 0.17 mmol), (2S,4R)-1-((S)-2-amino-3,3-dimethylbutanoyl)-4-hydroxy-N-(4-(4-methylthiazol-5-yl)benzyl)pyrrolidine-2-carboxamide, HCl (79 mg, 0.17 mmol) and triethylamine (94 µl, 0.67 mmol) in DMF (3269 µl) at 20°C under nitrogen. The resulting mixture was stirred at 20 °C for 30 minutes. The reaction mixture was diluted with EtOAc (50 mL), and washed sequentially with water (50 mL) and saturated brine (25 mL). The organic layer was dried with MgSO4, filtered and evaporated to afford crude product. The crude product was purified by preparative HPLC (Waters CSH C18 OBD column, 30 x 100 mm id, 5 micron particle size),using decreasingly polar mixtures of water (containing 1% by volume of NH4OH (28-30% in H2O)) and MeCN as eluents. Fractions containing the desired compound were evaporated to dryness to afford the title compound (80 mg, 47 %) as a white solid; ^{1}H NMR (400 MHz, $CDCl_3$, 30°C) 0.94 (9H, s), 1.10 (3H, d), 1.20 (6H, dd), 1.85 – 2 (2H, m), 2.01 – 2.12 (1H, m), 2.40 (1H, dd), 2.48 (3H, s), 2.52 – 2.68 (3H, m), 2.73 (2H, t), 2.84 (1H, dd), 3.07 (1H, dd), 3.42 – 3.53 (3H, m), 3.55 – 3.72 (2H, m), 3.76 – 3.97 (2H, m), 4.09 (1H, d), 4.31 (1H, dd), 4.45 – 4.62 (3H, m), 4.71 (1H, t), 4.92 – 5.02 (2H, m), 5.21 (1H, s), 6.36 – 6.53 (2H, m), 7.03 – 7.13 (2H, m), 7.12 – 7.24 (5H, m), 7.27 – 7.39 (6H, m), 7.46 – 7.57 (1H, m), 8.08 (1H, s), 8.64 (1H, s); ^{13}C NMR (126 MHz, DMSO-d6, 27°C) 12.5, 15.6, 24.2, 24.5, 25.9, 26.3, 30.5, 31.2, 35.5, 37.6, 41.4, 50.0 - 50.5, 55.4, 55.8, 56.3, 58.5, 68.6, 69.1, 69.7 - 69.8 , 96.7, 98.8, 106.1, 109.2, 110.6, 117.1, 117.8, 120.0, 125.1, 126.7, 127.2, 127.5, 127.8, 128.2, 128.4, 129.4, 130.8, 132.3, 135.9, 135.9, 139.1, 141.5, 147.4, 151.1, 159.1, 161.9, 168.2, 168.9, 171.4.*; m/z*: ES+ $[M+H]^+$ 1007.4; LCMS purity = 100%; ESI-HRMS calculated for $C_{56}H_{66}F_3N_6O_6S$ $[M+H]^+$: 1007.4711, observed 1007.4688.

Ethyl 2-((3-(3-((*tert*-butyldimethylsilyl)oxy)propyl)benzyl)oxy)acetate (15a)

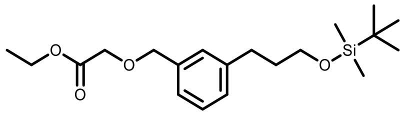

Ethyl 2-diazoacetate (0.222 mL, 2.11 mmol) in DCM (1.232 mL) was added slowly to (3-(3-((tert-butyldimethylsilyl)oxy)propyl)phenyl)methanol (394mg, 1.40 mmol) and rhodium acetate dimer (31 mg, 0.07 mmol) in DCM (3.2 mL) at 20°C over a period of 1 hour under nitrogen. The resulting solution which had darkened in colour was stirred at 20 °C for 18 hours. The mixture was diluted with water (10 mL) and the DCM layer was separated, washed with more water (20 mL) then passed through phase separating filter and evaporated to dryness. The crude product was purified by flash silica chromatography, elution gradient 0 to 20% EtOAc in heptane. Pure fractions were evaporated

to dryness to the title compound (349 mg, 68 %) as a colourless gum; ^{1}H NMR (400 MHz, $CDCl_3$, 30°C) 0.05 (6H, s), 0.91 (9H, s), 1.29 (3H, t), 1.78 – 1.89 (2H, m), 2.63 – 2.72 (2H, m), 3.63 (2H, t), 4.08 (2H, s), 4.23 (2H, q), 4.61 (2H, s), 7.13 (1H, d), 7.18 (2H, d), 7.26 (1H, s); *m/z*: ES+ $[M+H]^+$ 367.3.

Ethyl 2-((3-(3-hydroxypropyl)benzyl)oxy)acetate (15b)

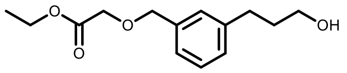

A solution of 1M TBAF in THF (1.43 mL, 1.43 mmol) was added in one portion to ethyl 2-((3-(3-((tert-butyldimethylsilyl)oxy)propyl)benzyl)oxy)acetate (349 mg, 0.95 mmol) in THF (8 mL) at 20 °C. The resulting solution was stirred for 2 hours. The reaction mixture was diluted with EtOAc (50 mL), and washed sequentially with saturated NH_4Cl (20 mL), water (20 mL), and saturated brine (20 mL). The organic layer was dried with $MgSO_4$, filtered and evaporated to afford crude product. The crude product was purified by flash silica chromatography, elution gradient 0 to 50% EtOAc in heptane to afford the title compound (144 mg, 60 %) as a yellow gum; ^{1}H NMR (400 MHz, $CDCl_3$, 30°C) 1.23 – 1.33 (4H, m), 1.86 – 1.95 (2H, m), 2.68 – 2.75 (2H, m), 3.67 (2H, q), 4.09 (2H, s), 4.23 (2H, q), 4.61 (2H, s), 7.15 (1H, d), 7.17 – 7.23 (2H, m), 7.28 (1H, d).

Ethyl 2-((3-(3-(3,5-difluoro-4-((1*R*,3*R*)-2-(2-fluoro-2-methylpropyl)-3-methyl-2,3,4,9-tetrahydro-1*H*-pyrido[3,4-b]indol-1-yl)phenoxy)propyl)benzyl)oxy)acetate (15c)

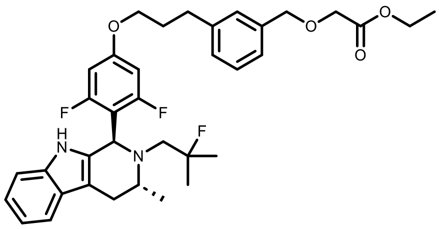

DIAD (0.11 mL, 0.57 mmol) was added dropwise to a stirred solution of 3,5-difluoro-4-((1R,3R)-2-(2-fluoro-2-methylpropyl)-3-methyl-2,3,4,9-tetrahydro-1H-pyrido[3,4-b]indol-1-yl)phenol (111 mg, 0.29 mmol), ethyl 2-((3-(3-hydroxypropyl)benzyl)oxy)acetate (144mg, 0.57 mmol) and triphenylphosphine (150 mg, 0.57 mmol) in DCM (5 ml) at 20 °C. The resulting mixture was stirred at 20 °C for 30 minutes. DCM (15 mL) and water (25 mL) were added and the layers were separated and concentrated to give the crude product. The crude product was purified by flash silica chromatography, elution gradient 0 to 30% EtOAc in heptane. Pure fractions were evaporated to dryness to afford the title compound (130 mg, 73 %) as a yellow gum; ^{1}H NMR (400 MHz, $CDCl_3$,

30°C) 1.10 (3H, d), 1.14 – 1.32 (9H, m), 2.08 (2H, dq), 2.39 (1H, dd), 2.60 (1H, dd), 2.75 – 2.94 (3H, m), 3.09 (1H, dd), 3.68 (1H, d), 3.90 (2H, t), 4.08 (2H, s), 4.22 (2H, q), 4.60 (2H, s), 5.19 (1H, s), 6.38 (2H, d), 7.04 – 7.17 (3H, m), 7.17 – 7.24 (3H, m), 7.29 (1H, d), 7.46 – 7.55 (2H, m); *m/z*: ES+ $[M+H]^+$ 623.3.

2-((3-(3-(3,5-Difluoro-4-((1*R*,3*R*)-2-(2-fluoro-2-methylpropyl)-3-methyl-2,3,4,9-tetrahydro-1*H*-pyrido[3,4-b]indol-1-yl)phenoxy)propyl)benzyl)oxy)acetic acid (15d)

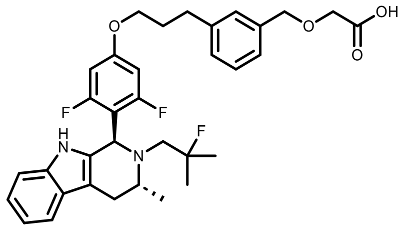

Lithium hydroxide hydrate (18 mg, 0.42 mmol) was added in one portion to ethyl 2-((3-(3-(3,5-difluoro-4-((1R,3R)-2-(2-fluoro-2-methylpropyl)-3-methyl-2,3,4,9-tetrahydro-1H-pyrido[3,4-b]indol-1-yl)phenoxy)propyl)benzyl)oxy)acetate (130mg, 0.21 mmol) in THF (0.8 mL) and water (0.2 mL) at 20°C. The resulting solution was stirred at 20 °C for 30 minutes. The reaction mixture was diluted with water (10 mL) then was acidified with 2M HCl and extracted into EtOAc (50 mL). The organic layer was washed with brine (15 mL) and evaporated to afford the title compound (124 mg, 100 %) as a yellow gum which was used in the next step without futher purification; *m/z*: ES+ $[M+H]^+$ 595.3.

(2*S*,4*R*)-1-((*S*)-2-(2-((3-(3-(3,5-Difluoro-4-((1*R*,3*R*)-2-(2-fluoro-2-methylpropyl)-3-methyl-2,3,4,9-tetrahydro-1*H*-pyrido[3,4-b]indol-1-yl)phenoxy)propyl)benzyl)oxy)acetamido)-3,3-dimethylbutanoyl)-4-hydroxy-*N*-(4-(4-methylthiazol-5-yl)benzyl)pyrrolidine-2-carboxamide (15)

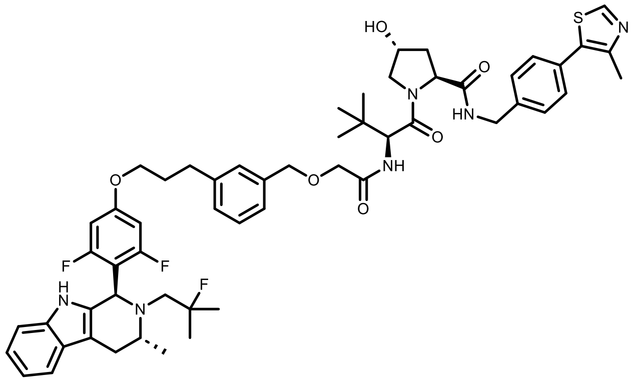

HATU (119 mg, 0.31 mmol) was added in one portion to 2-((3-(3-(3,5-difluoro-4-((1R,3R)-2-(2-fluoro-2-methylpropyl)-3-methyl-2,3,4,9-tetrahydro-1H-pyrido[3,4-b]indol-1-yl)phenoxy)propyl)benzyl)oxy)acetic acid (124mg, 0.21 mmol), (2S,4R)-1-((S)-2-amino-3,3-dimethylbutanoyl)-4-hydroxy-N-(4-(4-methylthiazol-5-yl)benzyl)pyrrolidine-2-carboxamide, HCl (97 mg, 0.21 mmol) and triethylamine (0.12 mL, 0.83 mmol) in DMF (4 mL) at 20°C under nitrogen. The resulting mixture was stirred at 20 °C for 30 minutes. The reaction mixture was diluted with EtOAc (50 mL), and washed sequentially with water (50 mL) and saturated brine (25 mL). The organic layer was dried with MgSO4, filtered and evaporated to afford crude product. The crude product was purified by preparative HPLC (Waters CSH C18 OBD column, 30 x 100 mm id, 5 micron particle size),using decreasingly polar mixtures of water (containing 1% by volume of NH4OH (28%-30% in H2O)) and MeCN as eluents. Fractions containing the desired compound were evaporated to dryness to afford the title compound (124 mg, 59 %) as a pale yellow solid; ^{1}H NMR (400 MHz, $CDCl_3$, 27°C) 0.94 (9H, s), 1.10 (3H, d), 1.21 (6H, dd), 2.07 (3H, p), 2.41 (1H, dd), 2.49 (3H, s), 2.59 (3H, dq), 2.74 – 2.92 (3H, m), 3.07 (1H, dd), 3.62 (2H, ddd), 3.8 – 3.97 (4H, m), 4.06 (1H, d), 4.31 (1H, dd), 4.47 – 4.62 (5H, m), 4.73 (1H, t), 5.20 (1H, s), 6.31 (2H, d), 7.03 – 7.19 (5H, m), 7.2 – 7.25 (2H, m), 7.27 – 7.31 (2H, m), 7.32 – 7.39 (4H, m), 7.47 – 7.55 (1H, m), 8.26 (1H, s), 8.64 (1H, s); ^{13}C NMR (126 MHz, DMSO-d6, 27°C) 12.5, 15.6, 24.2, 24.6, 25.9, 26.3, 29.7, 31.0, 35.5, 37.6, 41.4, 50.3, 55.4, 55.8, 56.3, 58.5, 67.4, 68.6, 72.2, 96.7, 98.4, 106.0, 108.9, 110.6, 117.1, 117.8, 120.0, 125.0, 126.7, 127.2, 127.4, 127.5, 128.1, 128.4, 129.4, 130.8, 132.4, 135.9, 137.4, 139.1, 141.1, 147.5, 151.1, 159.3, 162.0, 168.0, 168.9, 171.4; *m/z*: ES+ $[M+H]^+$ 1007.4, LCMS purity = 100%; ESI-HRMS calculated for $C_{56}H_{66}F_3N_6O_6S$ $[M+H]^+$: 1007.4711, observed 1007.4688

iv. LCMS chromatograms of final compounds

<u>(2*S*,4*R*)-1-((*S*)-2-(2-(2-(3,5-Difluoro-4-((1*R*,3*R*)-2-(2-fluoro-2-methylpropyl)-3-methyl-2,3,4,9-tetrahydro-1*H*-pyrido[3,4-*b*]indol-1-yl)phenoxy)ethoxy)acetamido)-3,3-dimethylbutanoyl)-4-hydroxy-*N*-(4-(4-methylthiazol-5-yl)benzyl)pyrrolidine-2-carboxamide (1)</u>

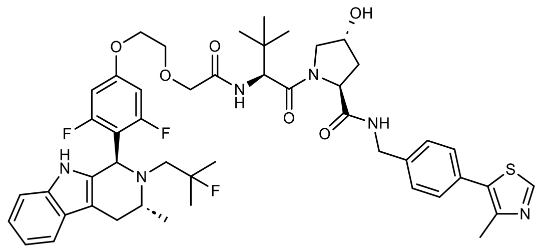

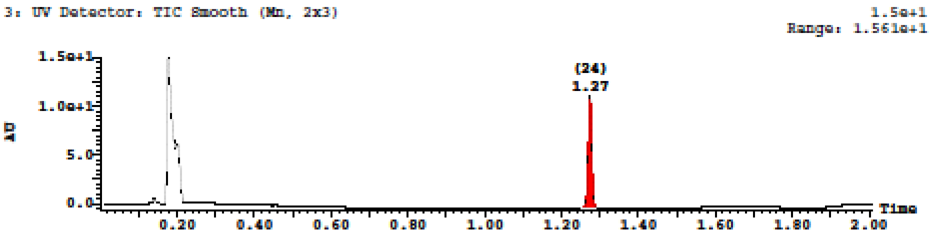

Peak Number	Compound	Time	AreaAbs	Area %Total	Mass Found
24		1.27	1e+005	100.00	

<u>(2*S*,4*R*)-1-((*S*)-2-(2-(2-(2-(3,5-Difluoro-4-((1*R*,3*R*)-2-(2-fluoro-2-methylpropyl)-3-methyl-2,3,4,9-tetrahydro-1H-pyrido[3,4-*b*]indol-1-yl)phenoxy)ethoxy)ethoxy)acetamido)-3,3-dimethylbutanoyl)-4-hydroxy-*N*-(4-(4-methylthiazol-5-yl)benzyl)pyrrolidine-2-carboxamide (2)</u>

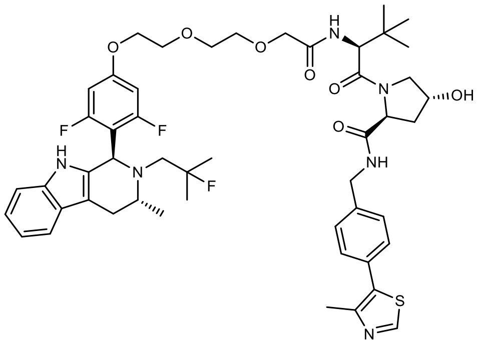

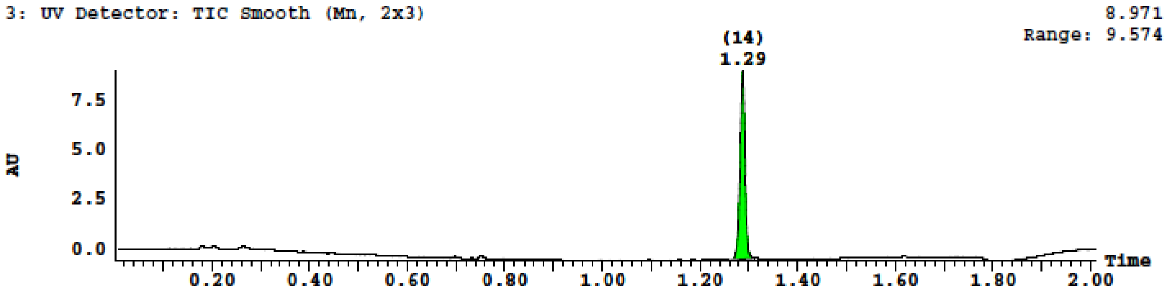

Peak Number	Compound	Time	AreaAbs	Area %Total	Mass Found
14	Found	1.29	1e+005	100.00	947.00

<u>(2*S*,4*R*)-1-((*S*)-2-(*tert*-Butyl)-14-(3,5-difluoro-4-((1*R*,3*R*)-2-(2-fluoro-2-methylpropyl)-3-methyl-2,3,4,9-tetrahydro-1*H*-pyrido[3,4-*b*]indol-1-yl)phenoxy)-4-oxo-6,9,12-trioxa-3-azatetradecanoyl)-4-hydroxy-*N*-(4-(4-methylthiazol-5-yl)benzyl)pyrrolidine-2-carboxamide (3)</u>

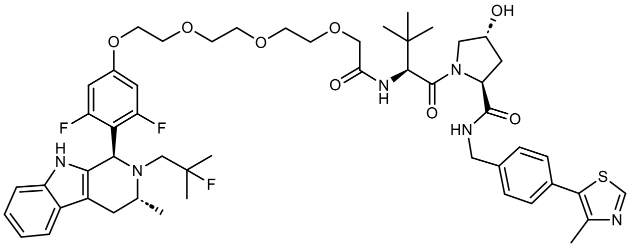

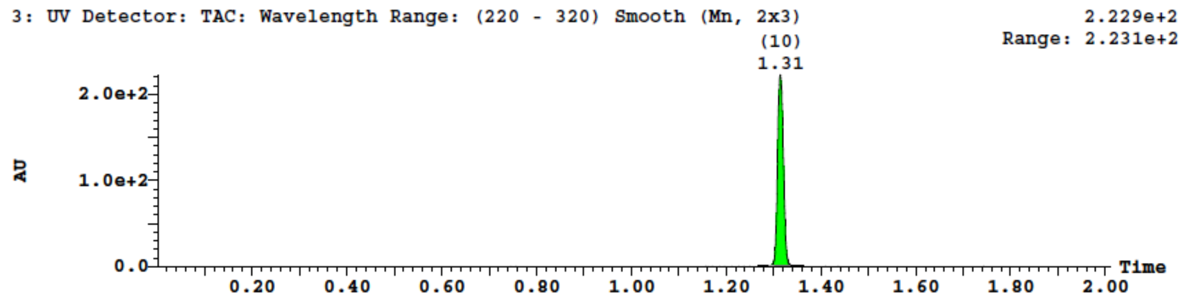

Peak Number	Compound	Time	AreaAbs	Area %Total	Mass Found	TIC
10	Found	1.31	3e+006	100.00	ınd, 1018.5000	)9509

<u>(2*S*,4*R*)-1-((*S*)-2-(*tert*-Butyl)-17-(3,5-difluoro-4-((1*R*,3*R*)-2-(2-fluoro-2-methylpropyl)-3-methyl-2,3,4,9-tetrahydro-1*H*-pyrido[3,4-*b*]indol-1-yl)phenoxy)-4-oxo-6,9,12,15-tetraoxa-3-azaheptadecanoyl)-4-hydroxy-*N*-(4-(4-methylthiazol-5-yl)benzyl)pyrrolidine-2-carboxamide (4)</u>

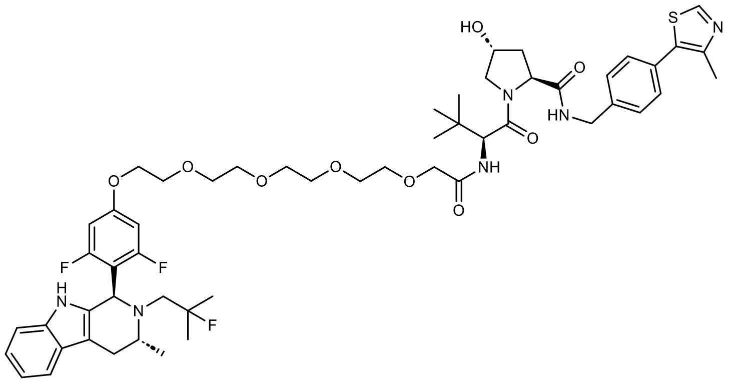

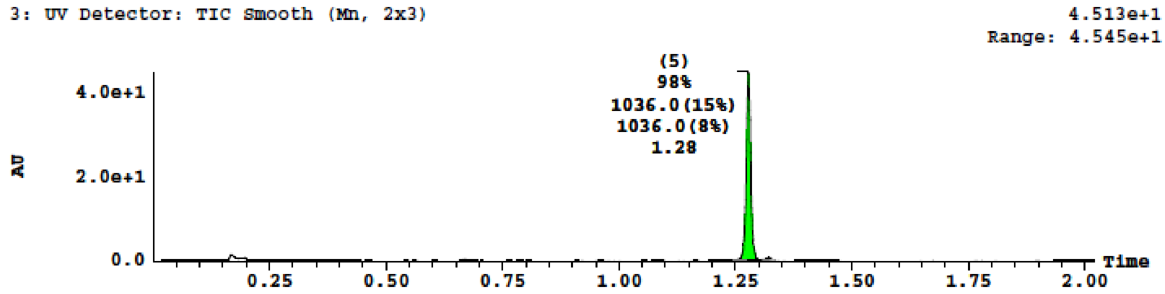

Peak Number	Compound	Time	AreaAbs	Area %Total	Mass Found
5	Found	1.28	5e+005	98.16	36.00, 1036.00
6		1.32	1e+004	1.84	

<u>(2*S*,4*R*)-1-((*S*)-2-(2-(3-(3-(3,5-Difluoro-4-((1*R*,3*R*)-2-(2-fluoro-2-methylpropyl)-3-methyl-2,3,4,9-tetrahydro-1*H*-pyrido[3,4-b]indol-1-yl)phenoxy)propoxy)propoxy)acetamido)-3,3-dimethylbutanoyl)-4-hydroxy-*N*-(4-(4-methylthiazol-5-yl)benzyl)pyrrolidine-2-carboxamide (5)</u>

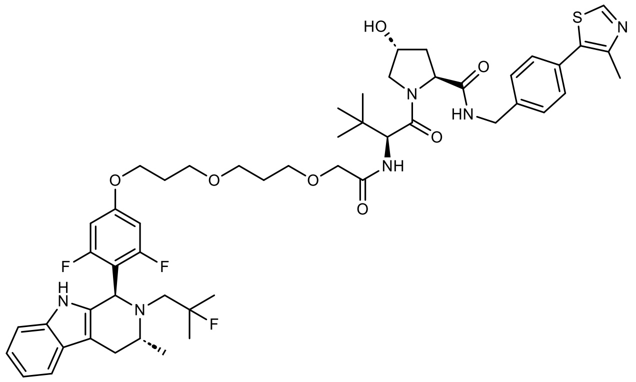

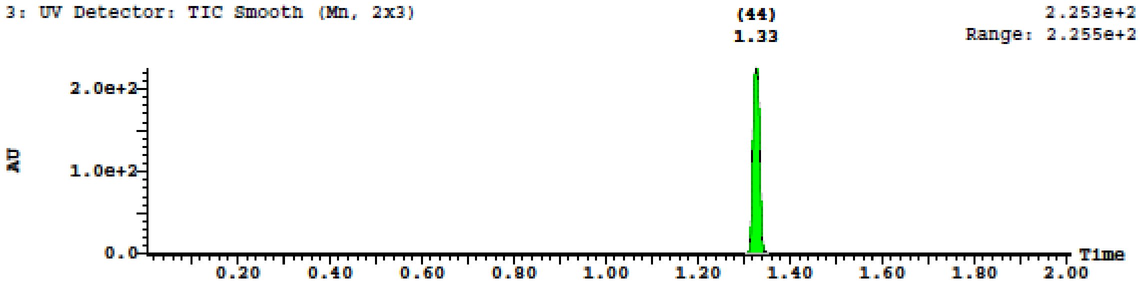

Peak Number	Compound	Time	AreaAbs	Area %Total	Mass Found
44	Found	1.33	4e+006	100.00	974.50, 974.50

(2S,4R)-1-((S)-2-(2-(2-(4-(2-(3,5-Difluoro-4-((1R,3R)-2-(2-fluoro-2-methylpropyl)-3-methyl-2,3,4,9-tetrahydro-1H-pyrido[3,4-b]indol-1-yl)phenoxy)ethyl)piperazin-1-yl)ethoxy)acetamido)-3,3-dimethylbutanoyl)-4-hydroxy-N-(4-(4-methylthiazol-5-yl)benzyl)pyrrolidine-2 (6)

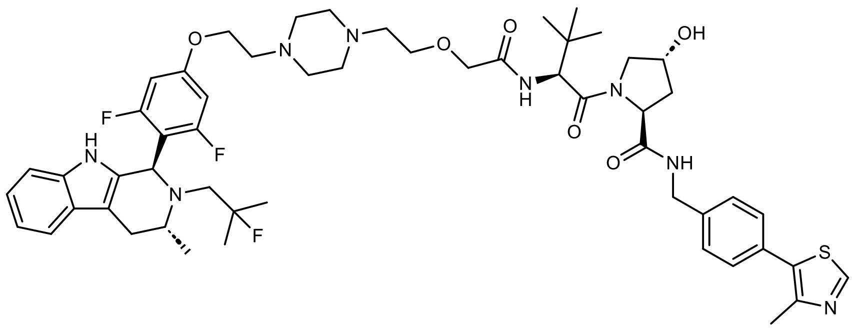

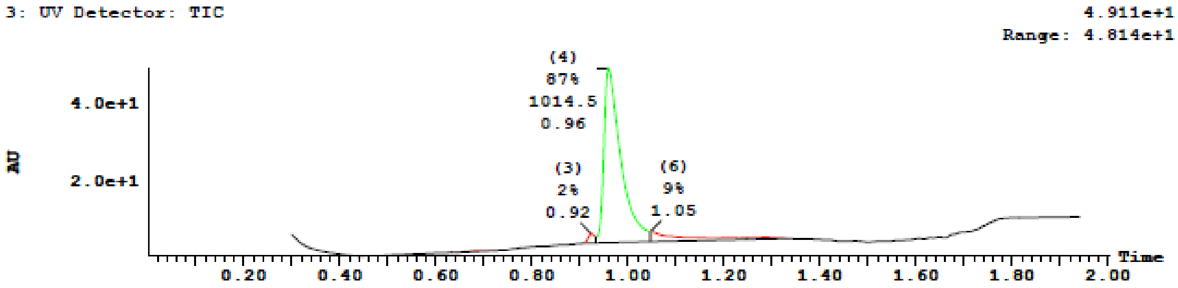

Peak Number	Compound	Time	AreaAbs	Area %Total	Width	Height	Mass Found
2		0.69	1e+004	0.59	0	4e+005	
3		0.92	4e+004	1.81	0	2e+006	
4	Found	0.96	2e+006	87.32	0	4e+007	1014.50
6		1.05	2e+005	8.85	0	3e+006	
7		1.29	3e+004	1.44	0	5e+005	

(2*S*,4*S*)-1-((*S*)-2-(2-(3-(3-(3,5-Difluoro-4-((1*R*,3*R*)-2-(2-fluoro-2-methylpropyl)-3-methyl-2,3,4,9-tetrahydro-1*H*-pyrido[3,4-*b*]indol-1-yl)phenoxy)propoxy)propoxy)acetamido)-3,3-dimethylbutanoyl)-4-hydroxy-*N*-(4-(4-methylthiazol-5-yl)benzyl)pyrrolidine-2-carboxamide (7)

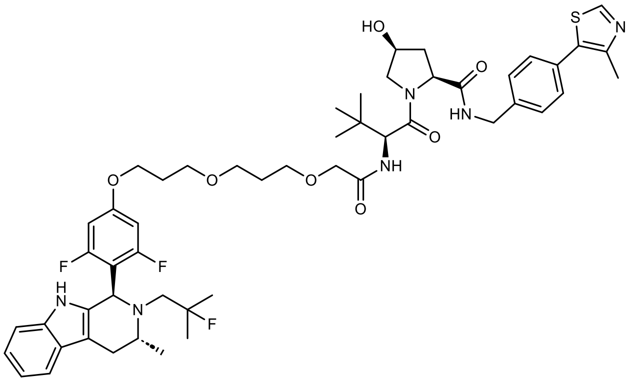

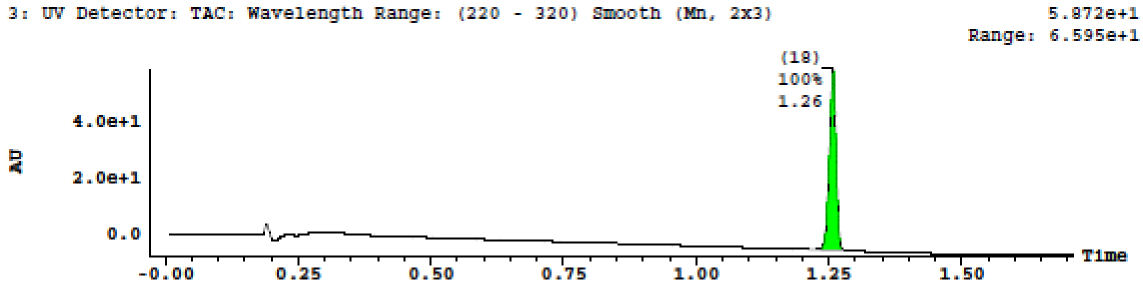

Peak Number	Compound	Time	AreaAbs	Area %Total	Mass Found	TIC
18	Found	1.26	9e+005	100.00	000, 975.0000	0628

3-(3,5-Difluoro-4-((1*R*,3*R*)-2-(2-fluoro-2-methylpropyl)-3-methyl-2,3,4,9-tetrahydro-1*H*-pyrido[3,4-*b*]indol-1-yl)phenoxy)propanoic acid (8)

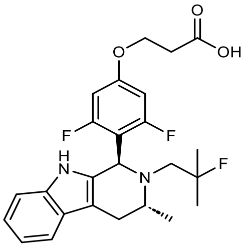

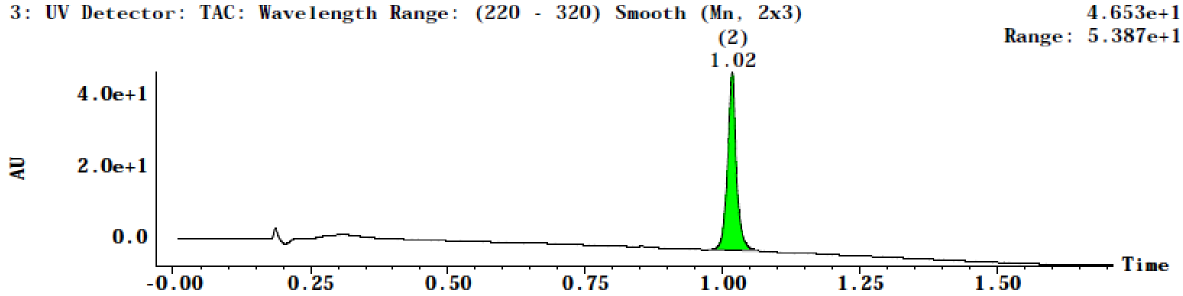

Peak Number	Compound	Time	AreaAbs	Area %Total	Mass Found	TIC
2	Tentative	1.02	916048	100.00	und, 461.0000	4600

(2S,4R)-1-((S)-2-(2-(2-(4-(3,5-Difluoro-4-((1R,3R)-2-(2-fluoro-2-methylpropyl)-3-methyl-2,3,4,9-tetrahydro-1H-pyrido[3,4-b]indol-1-yl)phenoxy)butoxy)ethoxy)acetamido)-3,3-dimethylbutanoyl)-4-hydroxy-N-(4-(4-methylthiazol-5-yl)benzyl)pyrrolidine-2-carboxamide (9)

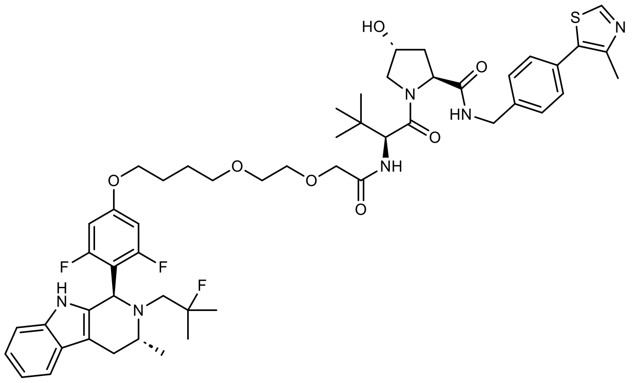

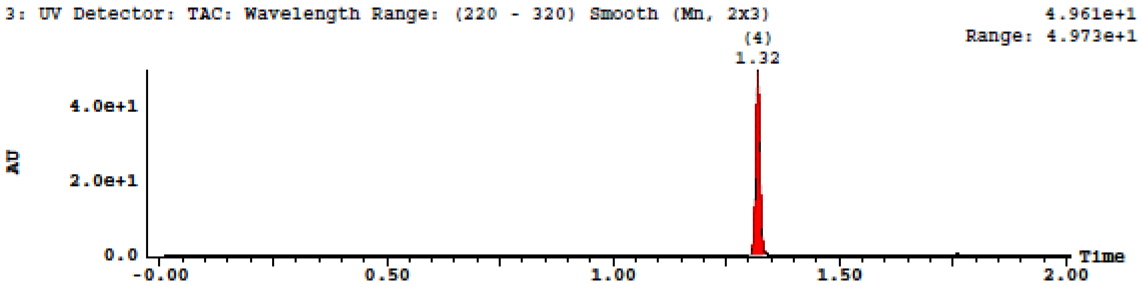

Peak Number	Compound	Time	AreaAbs	Area %Total	Mass Found	TIC
4		1.32	6e+005	100.00	Not Found	31996

(2*S*,4*R*)-1-((*S*)-2-(2-(3-(2-(3,5-Difluoro-4-((1*R*,3*R*)-2-(2-fluoro-2-methylpropyl)-3-methyl-2,3,4,9-tetrahydro-1*H*-pyrido[3,4-b]indol-1-yl)phenoxy)ethoxy)propoxy)acetamido)-3,3-dimethylbutanoyl)-4-hydroxy-*N*-(4-(4-methylthiazol-5-yl)benzyl)pyrrolidine-2-carboxamide (10)

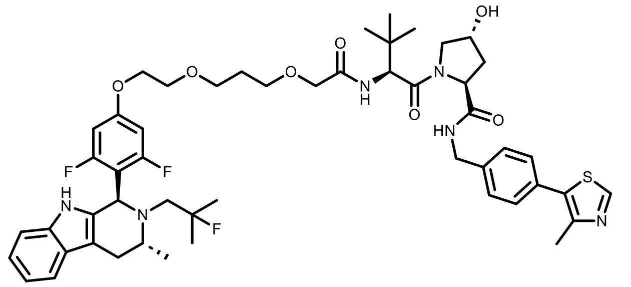

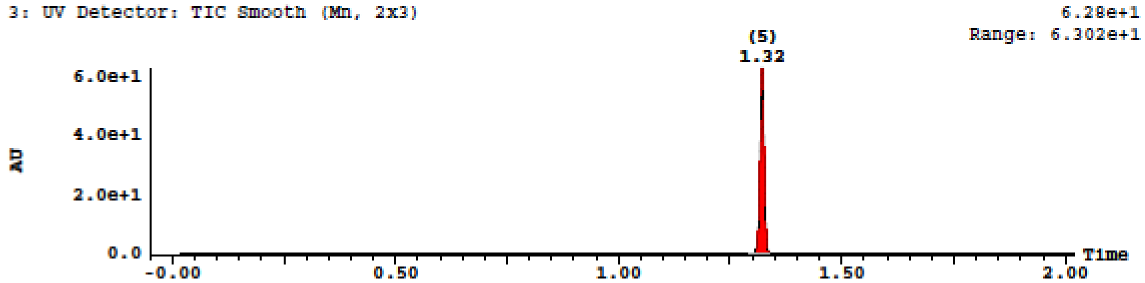

Peak Number	Compound	Time	AreaAbs	Area %Total	Mass Found
5		1.32	7e+005	100.00	

(2S,4R)-1-[(2S)-2-[[2-[[(cis)-2-[2-[3,5-Difluoro-4-[(1R,3R)-2-(2-fluoro-2-methyl-propyl)-3-methyl-1,3,4,9-tetrahydropyrido[3,4-b]indol-1-yl]phenoxy]ethoxymethyl]cyclopropyl]methoxy]acetyl]amino]-3,3-dimethyl-butanoyl]-4-hydroxy-N-[[4-(4-methylthiazol-5-yl)phenyl]methyl]pyrrolidine-2-carboxamide, isomer 1 (11)

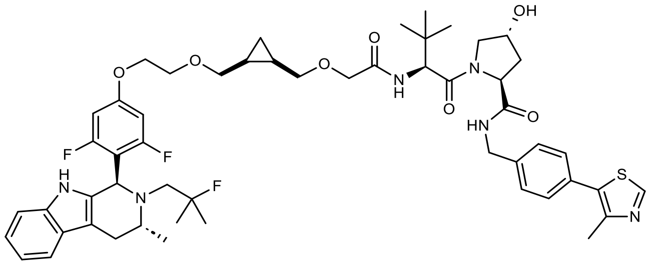

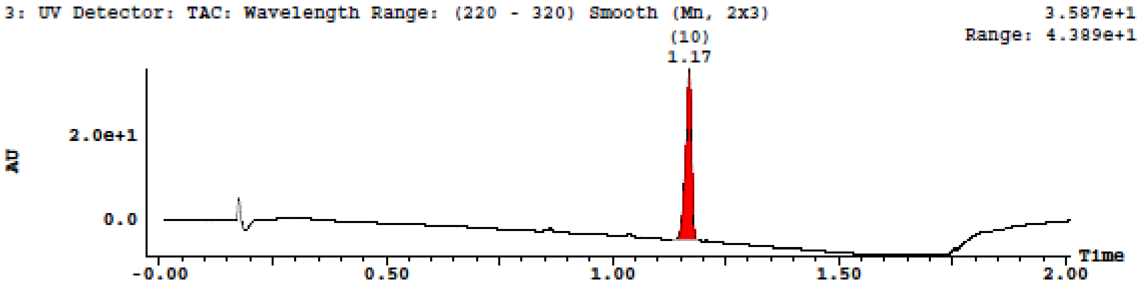

Peak Number	Compound	Time	AreaAbs	Area %Total	Mass Found	TIC
10		1.17	6e+005	100.00	Not Found	15532

(2S,4R)-1-[(2S)-2-[[2-[[(cis)-2-[2-[3,5-Difluoro-4-[(1R,3R)-2-(2-fluoro-2-methyl-propyl)-3-methyl-1,3,4,9-tetrahydropyrido[3,4-b]indol-1-yl]phenoxy]ethoxymethyl]cyclopropyl]methoxy]acetyl]amino]-3,3-dimethyl-butanoyl]-4-hydroxy-N-[[4-(4-methylthiazol-5-yl)phenyl]methyl]pyrrolidine-2-carboxamide, isomer 2 (12)

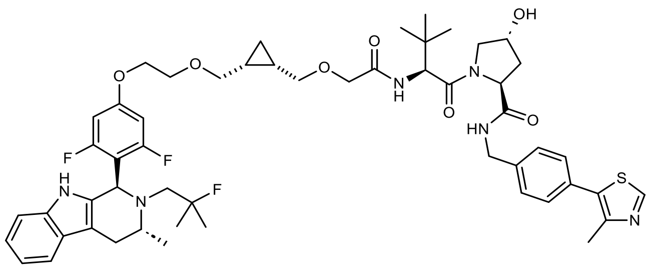

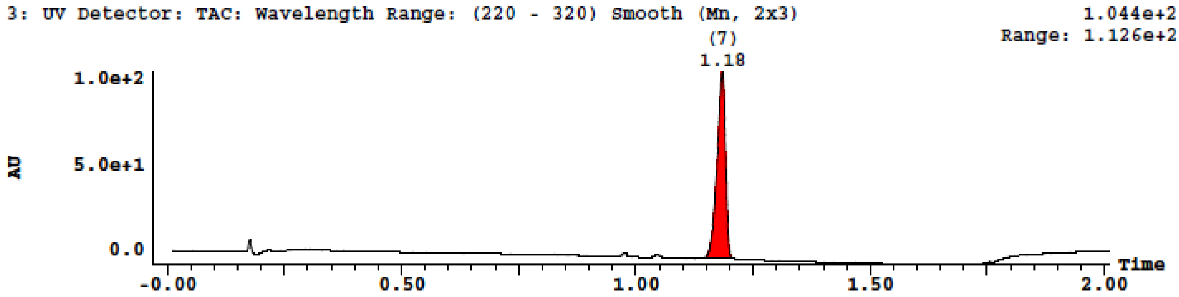

Peak Number	Compound	Time	AreaAbs	Area %Total	Mass Found	TIC
7		1.18	2e+006	100.00	Not Found	)2576

(2S,4R)-1-((S)-2-(1-(3-(3-(3,5-Difluoro-4-((1R,3R)-2-(2-fluoro-2-methylpropyl)-3-methyl-2,3,4,9-tetrahydro-1H-pyrido[3,4-b]indol-1-yl)phenoxy)propoxy)propoxy)cyclopropane-1-carboxamido)-3,3-dimethylbutanoyl)-4-hydroxy-N-(4-(4-methylthiazol-5-yl)benzyl)pyrrolidine-2-carboxamide (13)

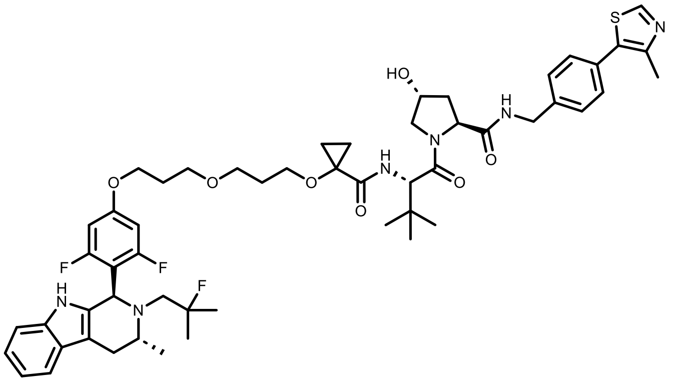

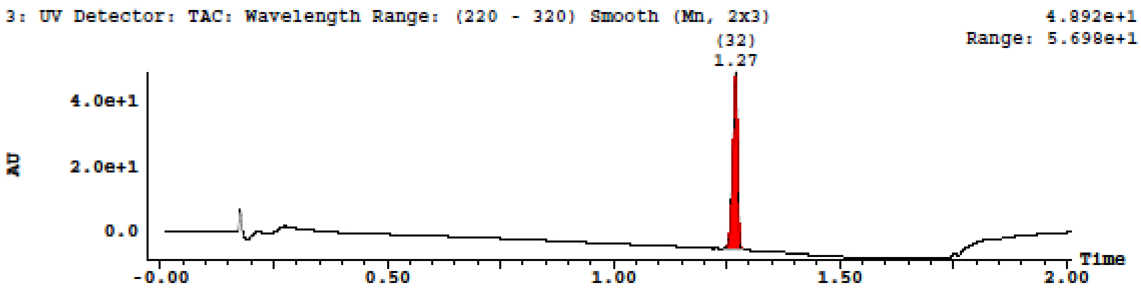

Peak Number	Compound	Time	AreaAbs	Area %Total	Mass Found	TIC
32		1.27	7e+005	100.00	Not Found	;0369

(2*S*,4*R*)-1-((*S*)-2-(2-(3-(3-((3,5-Difluoro-4-((1*R*,3*R*)-2-(2-fluoro-2-methylpropyl)-3-methyl-2,3,4,9-tetrahydro-1*H*-pyrido[3,4-b]indol-1-yl)phenoxy)methyl)phenyl)propoxy)acetamido)-3,3-dimethylbutanoyl)-4-hydroxy-*N*-(4-(4-methylthiazol-5-yl)benzyl)pyrrolidine-2-carboxamide (14)

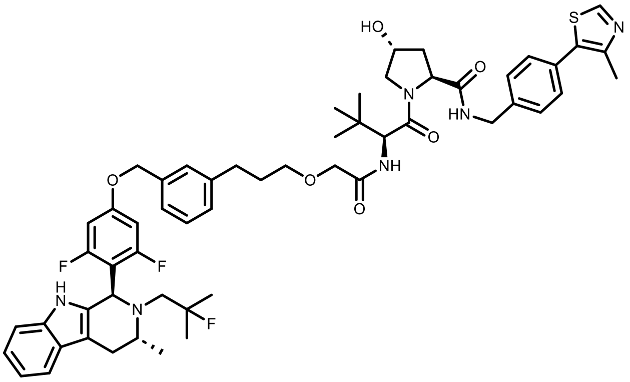

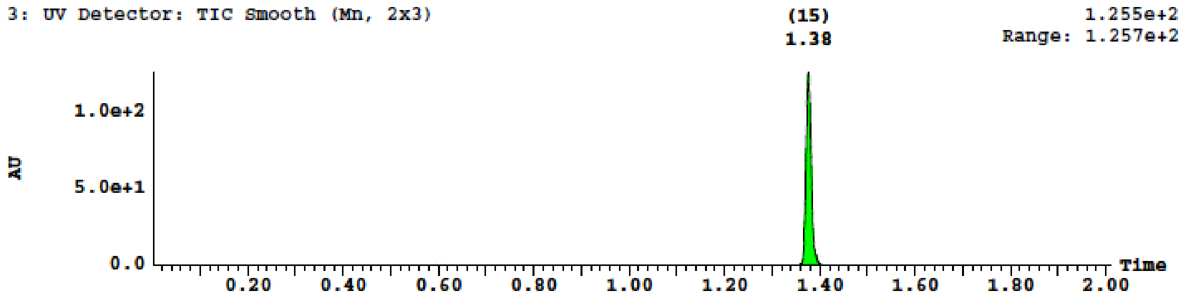

Peak Number	Compound	Time	AreaAbs	Area %Total	Mass Found
15	Found	1.38	2e+006	100.00	06.50, 1006.50

<u>(2*S*,4*R*)-1-((*S*)-2-(2-((3-(3-(3,5-Difluoro-4-((1*R*,3*R*)-2-(2-fluoro-2-methylpropyl)-3-methyl-2,3,4,9-tetrahydro-1*H*-pyrido[3,4-b]indol-1-yl)phenoxy)propyl)benzyl)oxy)acetamido)-3,3-dimethylbutanoyl)-4-hydroxy-*N*-(4-(4-methylthiazol-5-yl)benzyl)pyrrolidine-2-carboxamide (15)</u>

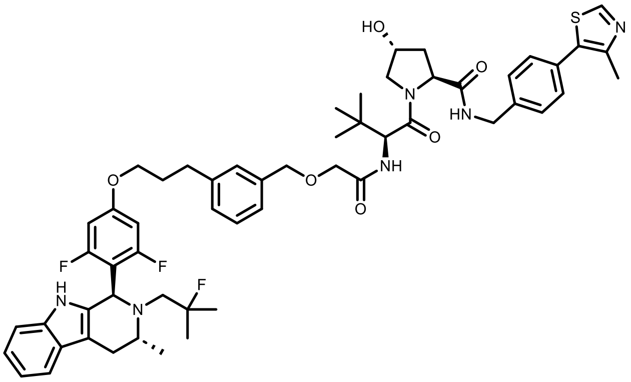

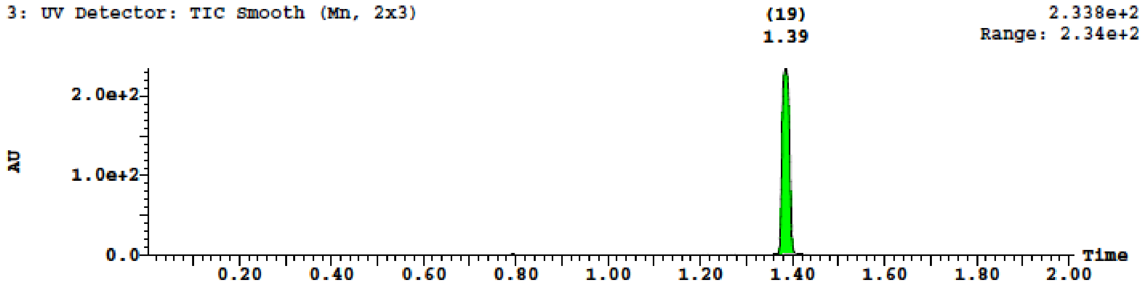

Peak Number	Compound	Time	AreaAbs	Area %Total	Mass Found
19	Found	1.39	4e+006	100.00	06.50, 1006.50

4. Table S4: Binding, degradation and mouse hepatocyte data for SAR exploration.

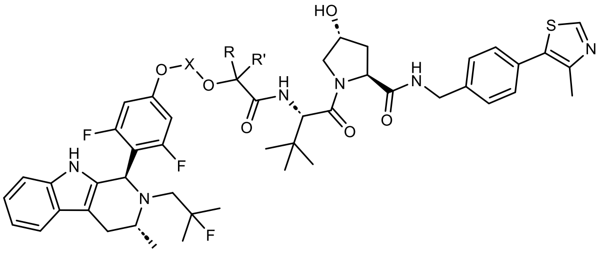

Compound Number	Linker (X)	R, R'	ERα binding IC_{50} (nM)	ERα degradation DC_{50} (nM)	ERα Dmax (%)#	muHeps (mL/min/10^6 cells)
**9**	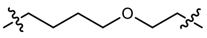	H	0.6	0.5 ± 0.1	100 ± 1	14 ± 0
**10**	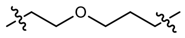	H	0.5 ± 0.1	0.4 ± 0.0	100 ± 1	20 ± 1
**11**	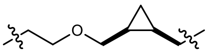	H	1.0 ± 0.2	0.5	96	ND
**12**	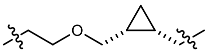	H	1.0 ± 0.3	0.7 ± 0.2	91 ± 0.1	ND
**13**	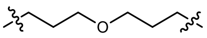	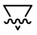	1.0 ± 0.3	2	96	ND
**14**	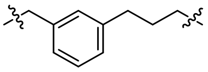	H	1.0 ± 0.0	4.9	103	ND
**15**	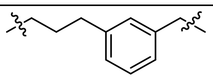	H	0.8 ± 0.0	14	106	ND

Table S4. Binding, degradation and mouse hepatocyte data for SAR exploration. # Maximum level of degradation observed for the compound where 0% is vehicle control and 100% is maximal concentration observed for fulvestrant. ND – not determined

5. Figure S9: Uncropped Western blotting images

Figure 2a

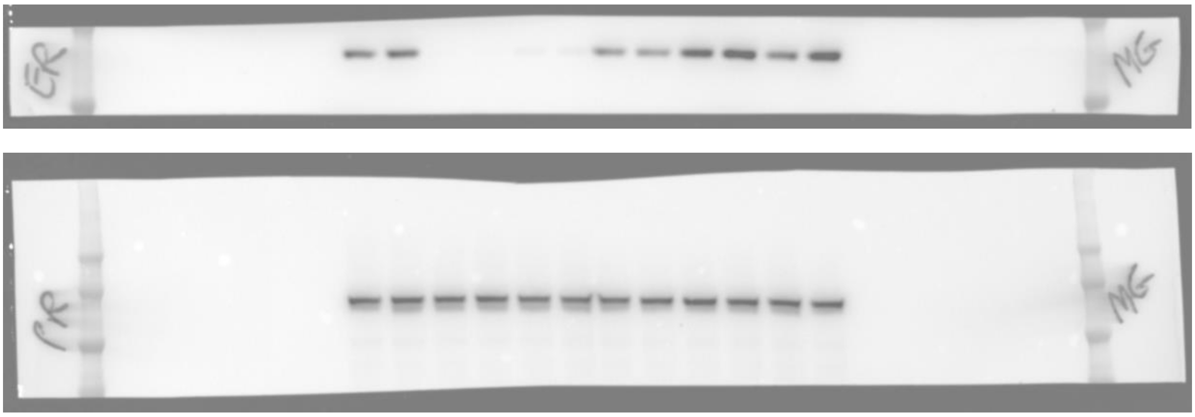

Figure 2b

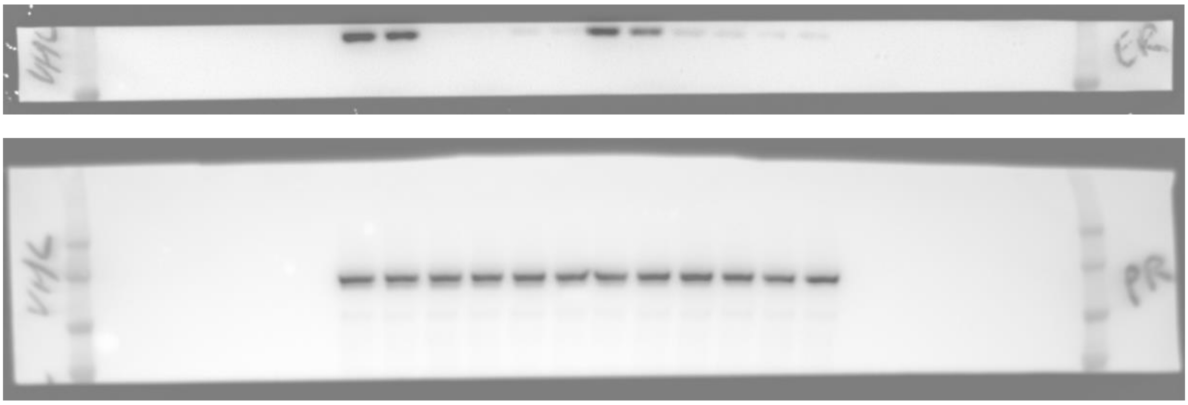

Figure 3a

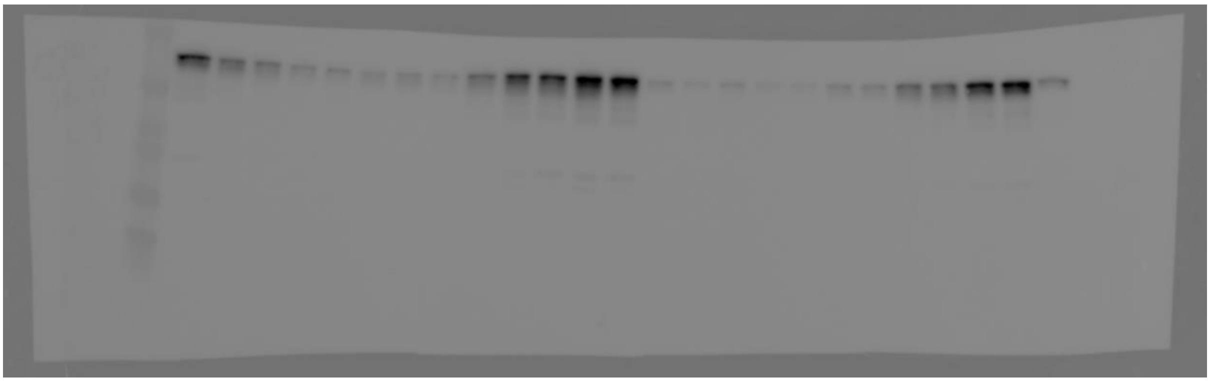

Figure 6a

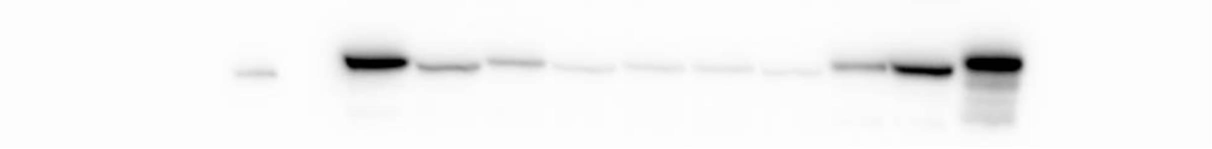

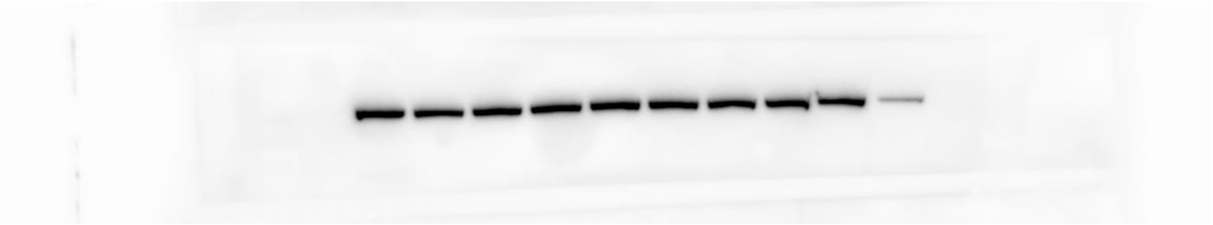

Figure 6g

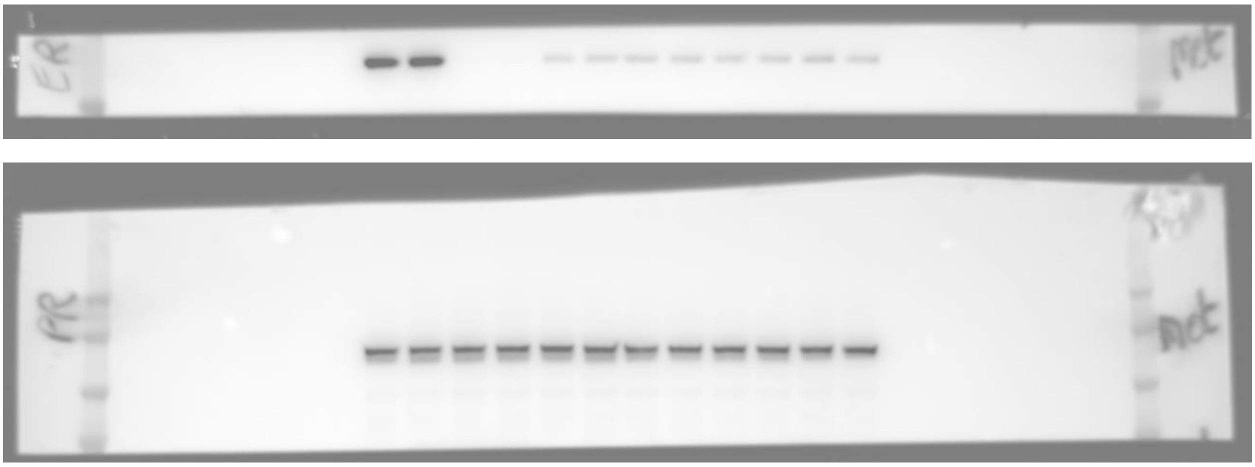

Figure 7a

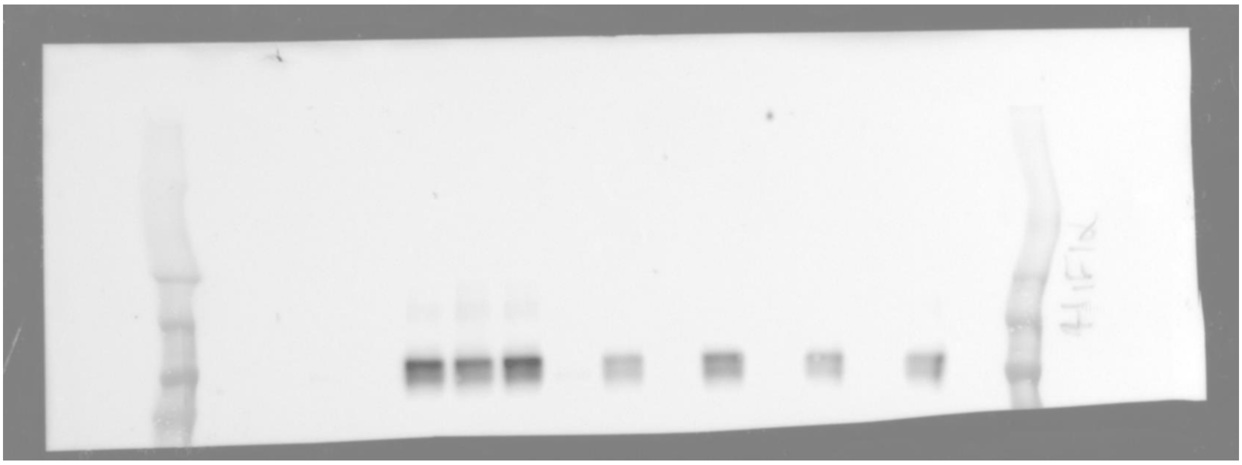

Figure 7b

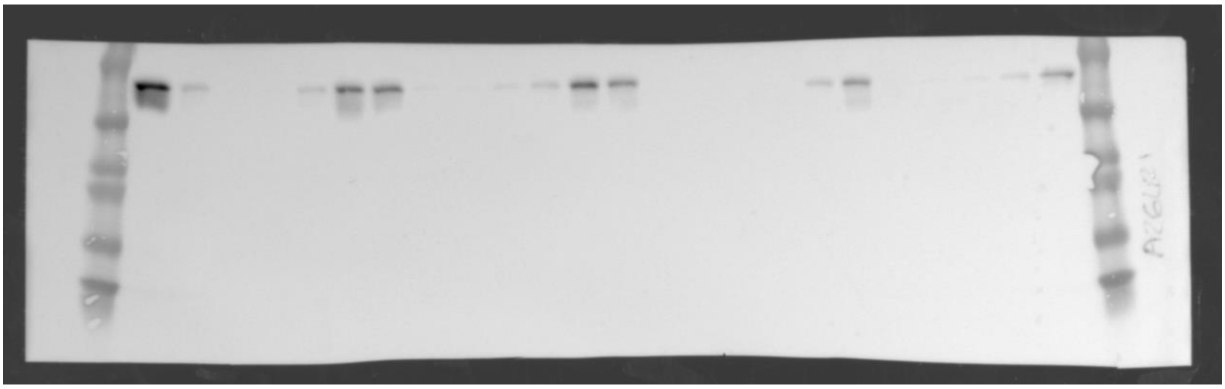

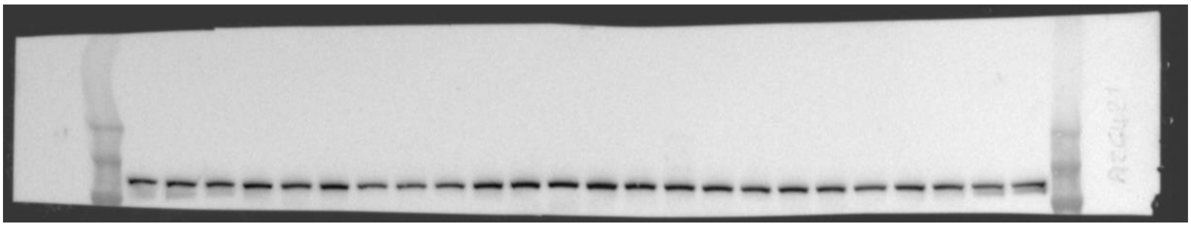

6. Supplementary References

1 Atilaw, Y. *et al.* Solution Conformations Shed Light on PROTAC Cell Permeability. *ACS Med Chem Lett* **12**, 107-114, doi:10.1021/acsmedchemlett.0c00556 (2021).

2 Abraham, M. H. *et al.* An NMR method for the quantitative assessment of intramolecular hydrogen bonding; application to physicochemical, environmental, and biochemical properties. *J Org Chem* **79**, 11075-11083, doi:10.1021/jo502080p (2014).

3 Garcia-Viloca, M., Gelabert, R., González-Lafont, À., Moreno, M. & Lluch, J. M. Temperature Dependence of Proton NMR Chemical Shift As a Criterion To Identify Low-Barrier Hydrogen Bonds. *Journal of the American Chemical Society* **120**, 10203-10209, doi:10.1021/ja9742141 (1998).

4 Drummond, M. L., Henry, A., Li, H. & Williams, C. I. Improved Accuracy for Modeling PROTAC-Mediated Ternary Complex Formation and Targeted Protein Degradation via New In Silico Methodologies. *J Chem Inf Model* **60**, 5234-5254, doi:10.1021/acs.jcim.0c00897 (2020).

5 Dragovich, P. S. *et al.* Antibody-Mediated Delivery of Chimeric BRD4 Degraders. Part 2: Improvement of In Vitro Antiproliferation Activity and In Vivo Antitumor Efficacy. *J Med Chem* **64**, 2576-2607, doi:10.1021/acs.jmedchem.0c01846 (2021).

6 Gadd, M. S. *et al.* Structural basis of PROTAC cooperative recognition for selective protein degradation. *Nat Chem Biol* **13**, 514-521, doi:10.1038/nchembio.2329 (2017).

7 Testa, A., Hughes, S. J., Lucas, X., Wright, J. E. & Ciulli, A. Structure-Based Design of a Macrocyclic PROTAC. *Angew Chem Int Ed Engl* **59**, 1727-1734, doi:10.1002/anie.201914396 (2020).

8 De Savi, C. *et al.* Optimization of a Novel Binding Motif to (E)-3-(3,5-Difluoro-4-((1R,3R)-2-(2-fluoro-2-methylpropyl)-3-methyl-2,3,4,9-tetra hydro-1H-pyrido[3,4-b]indol-1-yl)phenyl)acrylic Acid (AZD9496), a Potent and Orally Bioavailable Selective Estrogen Receptor Downregulator and Antagonist. *J Med Chem* **58**, 8128-8140, doi:10.1021/acs.jmedchem.5b00984 (2015).

9 Castanar, L., Sauri, J., Williamson, R. T., Virgili, A. & Parella, T. Pure in-phase heteronuclear correlation NMR experiments. *Angew Chem Int Ed Engl* **53**, 8379-8382, doi:10.1002/anie.201404136 (2014).

10 Hu, H. & Krishnamurthy, K. Revisiting the initial rate approximation in kinetic NOE measurements. *J Magn Reson* **182**, 173-177, doi:10.1016/j.jmr.2006.06.009 (2006).

11 Macur, S., Farmer, B. T. & Brown, L. R. An improved method for the determination of cross-relaxation rates from NOE data. *Journal of Magnetic Resonance (1969)* **70**, 493-499, doi:10.1016/0022-2364(86)90143-5 (1986).

12 Butts, C. P. *et al.* Interproton distance determinations by NOE--surprising accuracy and precision in a rigid organic molecule. *Org Biomol Chem* **9**, 177-184, doi:10.1039/c0ob00479k (2011).

13 Balazs, A. Y. S. *et al.* Free Ligand 1D NMR Conformational Signatures To Enhance Structure Based Drug Design of a Mcl-1 Inhibitor (AZD5991) and Other Synthetic Macrocycles. *J Med Chem* **62**, 9418-9437, doi:10.1021/acs.jmedchem.9b00716 (2019).

14 Crew, A. P. *et al.* Identification and Characterization of Von Hippel-Lindau-Recruiting Proteolysis Targeting Chimeras (PROTACs) of TANK-Binding Kinase 1. *J Med Chem* **61**, 583-598, doi:10.1021/acs.jmedchem.7b00635 (2018).

15 Zha, Z. *et al.* Multidentate (18)F-polypegylated styrylpyridines as imaging agents for Abeta plaques in cerebral amyloid angiopathy (CAA). *J Med Chem* **54**, 8085-8098, doi:10.1021/jm2009106 (2011).

16 Shan, Y. *et al.* Discovery of novel anti-angiogenesis agents. Part 11: Development of PROTACs based on active molecules with potency of promoting vascular normalization. *Eur J Med Chem* **205**, 112654, doi:10.1016/j.ejmech.2020.112654 (2020).

17 Williamson, B., Harlfinger, S. & McGinnity, D. F. Evaluation of the Disconnect between Hepatocyte and Microsome Intrinsic Clearance and In Vitro In Vivo Extrapolation Performance. *Drug Metab Dispos* **48**, 1137-1146, doi:10.1124/dmd.120.000131 (2020).
