# Supplementary material for: Metabolism-driven in vitro/in vivo disconnect of an oral ERɑ VHL-PROTAC
Source: Commun Biol. 2024 May 13;7:563. doi: 10.1038/s42003-024-06238-x (PMC11091220; doi:10.1038/s42003-024-06238-x)
Supplement: Supplementary file 3 — Description of Additional Supplementary Files [file 42003_2024_6238_MOESM3_ESM.pdf]

Description of Additional Supplementary Files

File name: Supplementary Data
Description: The source data behind the graphs and tables in the paper.
