# Supplementary material for: Metabolism-driven in vitro/in vivo disconnect of an oral ERɑ VHL-PROTAC
Source: Commun Biol. 2024 May 13;7:563. doi: 10.1038/s42003-024-06238-x (PMC11091220; doi:10.1038/s42003-024-06238-x)
Supplement: Supplementary file 5 — Reporting Summary [file 42003_2024_6238_MOESM5_ESM.pdf]

nature portfolio

Corresponding author(s): Thomas G Hayhow

Last updated by author(s): Mar 21, 2024

Reporting Summary

Nature Portfolio wishes to improve the reproducibility of the work that we publish. This form provides structure for consistency and transparency in reporting. For further information on Nature Portfolio policies, see our Editorial Policies and the Editorial Policy Checklist.

Statistics

For all statistical analyses, confirm that the following items are present in the figure legend, table legend, main text, or Methods section.

n/a	Confirmed	
☐	☒	The exact sample size (*n*) for each experimental group/condition, given as a discrete number and unit of measurement
☐	☒	A statement on whether measurements were taken from distinct samples or whether the same sample was measured repeatedly
☐	☒	The statistical test(s) used AND whether they are one- or two-sided<br>*Only common tests should be described solely by name; describe more complex techniques in the Methods section.*
☐	☒	A description of all covariates tested
☐	☒	A description of any assumptions or corrections, such as tests of normality and adjustment for multiple comparisons
☐	☒	A full description of the statistical parameters including central tendency (e.g. means) or other basic estimates (e.g. regression coefficient) AND variation (e.g. standard deviation) or associated estimates of uncertainty (e.g. confidence intervals)
☐	☒	For null hypothesis testing, the test statistic (e.g. *F*, *t*, *r*) with confidence intervals, effect sizes, degrees of freedom and *P* value noted<br>*Give P values as exact values whenever suitable.*
☒	☐	For Bayesian analysis, information on the choice of priors and Markov chain Monte Carlo settings
☒	☐	For hierarchical and complex designs, identification of the appropriate level for tests and full reporting of outcomes
☒	☐	Estimates of effect sizes (e.g. Cohen's *d*, Pearson's *r*), indicating how they were calculated

Our web collection on statistics for biologists contains articles on many of the points above.

Software and code

Policy information about availability of computer code

Data collection	NMR data were collected using TopSpin 4.0 (Bruker Biospin GmbH)
Data analysis	Cell proliferation data were plotted and non linear regression curve fitting analysis performed using GraphPad Prism 8.<br>ER degradation imaging assay data and ER binding data were plotted and IC50/DC50s calculated using Genedata Screener (Genedata AG multiple versions)<br>MD calculations were performed using desmond within Maestro (Schrödinger 2020, https://www.schrodinger.com/products/desmond)<br>QM was performed using MOE (Chemical Computing Group: Molecular 189 Operating Environment (MOE), version 2019.01)

For manuscripts utilizing custom algorithms or software that are central to the research but not yet described in published literature, software must be made available to editors and reviewers. We strongly encourage code deposition in a community repository (e.g. GitHub). See the Nature Portfolio guidelines for submitting code & software for further information.

Data

Policy information about availability of data

All manuscripts must include a data availability statement. This statement should provide the following information, where applicable:

- Accession codes, unique identifiers, or web links for publicly available datasets
- A description of any restrictions on data availability
- For clinical datasets or third party data, please ensure that the statement adheres to our policy

All data generated or analysed during this study are included in this published article (and its supplementary information files). Associated raw data for our Figures are available from the corresponding author on reasonable request.

Research involving human participants, their data, or biological material

Policy information about studies with human participants or human data. See also policy information about sex, gender (identity/presentation), and sexual orientation and race, ethnicity and racism.

Reporting on sex and gender	Not applicable
Reporting on race, ethnicity, or other socially relevant groupings	Not applicable
Population characteristics	Not applicable
Recruitment	Not applicable
Ethics oversight	Not applicable

Note that full information on the approval of the study protocol must also be provided in the manuscript.

Field-specific reporting

Please select the one below that is the best fit for your research. If you are not sure, read the appropriate sections before making your selection.

☒ Life sciences ☐ Behavioural & social sciences ☐ Ecological, evolutionary & environmental sciences

For a reference copy of the document with all sections, see nature.com/documents/nr-reporting-summary-flat.pdf

Life sciences study design

All studies must disclose on these points even when the disclosure is negative.

Sample size	For in vivo studies, group sizes were determined using power calculation based on historical growth data in the group, for the models. For the in vitro studies, sample sizes were selected based on what is generally accepted and published for each individual assay.
Data exclusions	No data was excluded
Replication	The majority of experiments were repeated at least 2-3 times on separate occasions (indicated in the methods/figure legend) and results were in concordance with each other
Randomization	For in vivo experiments, animals were randomised at selection for the live phase. For the PD analysis, samples were also randomised across gels for the western blotting analysis. In vitro studies were not randomised, but appropriate controls were run.
Blinding	Blinding was neither possible nor relevant for in vitro or in vivo experiments due to experimental setup to ensure correct sample labeling.

Reporting for specific materials, systems and methods

We require information from authors about some types of materials, experimental systems and methods used in many studies. Here, indicate whether each material, system or method listed is relevant to your study. If you are not sure if a list item applies to your research, read the appropriate section before selecting a response.

Materials & experimental systems

n/a	Involved in the study
☐	☒ Antibodies
☐	☒ Eukaryotic cell lines
☒	☐ Palaeontology and archaeology
☐	☒ Animals and other organisms
☒	☐ Clinical data
☒	☐ Dual use research of concern
☒	☐ Plants

Methods

n/a	Involved in the study
☒	☐ ChIP-seq
☒	☐ Flow cytometry
☒	☐ MRI-based neuroimaging

Antibodies

Antibodies used

GST: Thermo-Fisher #PV3550
ERα: Epredia #RM-9101-S, clone SP1 (Imaging assay)
ERα: Thermoscientific #RM-9101-S, clone SP1 (Western blotting)
PR: Dako #M356901-2, clone PgR 636
AlexaFluor 594 goat anti-rabbit IgG: Molecular Probes #A11012
AlexaFluor 488 goat anti-mouse IgG: Molecular Probes #A11001
HIF1α, BD bioscience #610958
Vinculin, Sigma #V9131
beta-actin, CST #4970

Validation

Antibody against GST was validated by the manufacturer with the LanthaScreen® TR-FRET LXR alpha Coactivator Assay (ThermoFisher PV4655) using a titration of the agonist T0901317 (Cayman Chemicals 71810).
Antibody against ERα was validated by the manufacturer by the presence of staining of ERα-expressing formalin-fixed and paraffin embedded normal or neoplastic tissues (e.g. breast, uterine, cervical) and absence of staining in tissues not expressing ERα (e.g. heart, thyroid, cerebellum).
Antibody against PR was validated to react against both PR-A and PR-B by Western blotting against cell lysates from T47D human PR-expressing breast cancer cells, and the absence of corresponding bands in MDA-231 PR-negative breast cancer cells (Press et al, 2002).
Other antibodies were obtained from reputable vendors. Refer to their websites for validation data and relevant citations for the species and application used in this study.

Eukaryotic cell lines

Policy information about cell lines and Sex and Gender in Research

Cell line source(s)

All cell lines were obtained from American Type Culture Collection (ATCC)

Authentication

All cell lines were authenticated by short tandem repeat analysis at the time of banking and used for a maximum of 15 cell passages.

Mycoplasma contamination

All cell lines tested negative for Mycoplasma at the time of banking and are used for a maximum of 15 cell passages

Commonly misidentified lines
(See ICLAC register)

None

Animals and other research organisms

Policy information about studies involving animals; ARRIVE guidelines recommended for reporting animal research, and Sex and Gender in Research

Laboratory animals

Female NSG (NOD.Cg-Prkdscid Il12rgtm1WjI/SzJ) obtained from Charles River UK, arriving at age 7 - 11 weeks.
Initial mouse PK studies were performed in male CD-1 strain. Male SCID mice were used for the enabling formulations (EF) studies.

Wild animals

Not applicable

Reporting on sex

Mouse strains chosen based on the strain particular tumour models were established in.

Field-collected samples

Not applicable

Ethics oversight

All work was done under project license 70/8839, 70/8894 and P0EC1FFDF granted by the Home Office of the United Kingdom.

Note that full information on the approval of the study protocol must also be provided in the manuscript.
